# A versatile route to fabricate single atom catalysts with high chemoselectivity and regioselectivity in hydrogenation

**DOI:** 10.1038/s41467-019-11619-6

**Published:** 2019-08-14

**Authors:** Xiaohui He, Qian He, Yuchen Deng, Mi Peng, Hongyu Chen, Ying Zhang, Siyu Yao, Mengtao Zhang, Dequan Xiao, Ding Ma, Binghui Ge, Hongbing Ji

**Affiliations:** 1 Fine Chemical Industry Research Institute, School of Chemistry, Sun Yat-sen University, Guangzhou, 510275 China; 2 Beijing National Laboratory for Molecular Sciences, College of Chemistry and Molecular Engineering and College of Engineering, and BIC-ESAT, Peking University, Beijing, 100871 China; 3 Center for Integrative Materials Discovery, Department of Chemistry and Chemical Engineering, University of New Haven, 300 Boston Post Road, West Haven, CT 06516 USA; 4 Beijing National Laboratory for Condensed Matter Physics, Institute of Physics, Chinese Academy of Sciences, Beijing, 100190 China; 5 Institute of Physical Science and Information Technology, Anhui University, Hefei, 230601 China; 6 School of Chemical Engineering, Guangdong University of Petrochemical Technology, Maoming, 525000 China

**Keywords:** Catalyst synthesis, Heterogeneous catalysis, Chemical engineering

## Abstract

Preparation of single atom catalysts (SACs) is of broad interest to materials scientists and chemists but remains a formidable challenge. Herein, we develop an efficient approach to synthesize SACs via a precursor-dilution strategy, in which metalloporphyrin (MTPP) with target metals are co-polymerized with diluents (tetraphenylporphyrin, TPP), followed by pyrolysis to N-doped porous carbon supported SACs (M_1_/N-C). Twenty-four different SACs, including noble metals and non-noble metals, are successfully prepared. In addition, the synthesis of a series of catalysts with different surface atom densities, bi-metallic sites, and metal aggregation states are achieved. This approach shows remarkable adjustability and generality, providing sufficient freedom to design catalysts at atomic-scale and explore the unique catalytic properties of SACs. As an example, we show that the prepared Pt_1_/N-C exhibits superior chemoselectivity and regioselectivity in hydrogenation. It only converts terminal alkynes to alkenes while keeping other reducible functional groups such as alkenyl, nitro group, and even internal alkyne intact.

## Abstract (web summary)

The general synthesis of single atom catalysts (SACs) is of broad interest to chemists but remains a formidable challenge. Here, with the precursor-dilution strategy, the authors successfully prepare 24 different SACs and the Pt SACs exhibit superior chemo- and regio-selectivity in hydrogenation.

## Introduction

Single atom catalysts (SACs), with maximum atom-utilization and unique electronic and geometric properties^1^, are becoming a thriving research field because of their enhanced catalytic performance in a wide scope of industrially important reactions, e.g., selective hydrogenation of nitroarenes, alkenes and carbonyl compounds^2–4^, catalytic transformation of methane^5,6^, aqueous reforming of methanol^7^, hydroformylation of olefins^8^, olefin metathesis^9^, and oxygen reduction^10,11^. Various approaches have been utilized to prepare SACs, including the methods of impregnation/ion-exchange/co-precipitation^6,12,13^, defect engineering^14^, iced-photochemistry^15^, atomic layer deposition^16,17^, galvanic replacement^18^, high-temperature migration^19^, and high-temperature pyrolysis^20,21^. However, developing general protocols that can be used to easily synthesize of a wide variety of SACs is still highly desirable. For example, by Jung et al., theoretical calculations were conducted to predict universal principles for the electro-catalytic performance of SACs bearing various metal sites^22^. But the difficulty arises on verifying such predictions in experiments, as there are no general routes to prepare SACs with different center metals but similar supports and coordination environment. In addition, as predicted by Beller et al., the preparation of bi-/multi-metallic SACs is regarded as a next breakthrough because of their significant importance in the domino and tandem reactions^23^, but there are few reports for their synthesis, mainly due to the huge obstacle to keep various metallic elements with obviously different physical/chemical properties coexisting in atomically dispersed states. Furthermore, comparative studies on the catalysis of different aggregation states, e.g., single atoms (SAs), nanoclusters (NCs), and nanoparticles (NPs), like the work by Zhang et al. on the Ru catalysts for CO_2_ methanation^24^, received extensive attention. But, most of these studies rely on tuning the aggregation states by changing the metal loadings^25,26^, which did not conform to the single-factor-variable research method. Thus, a facile approach to regulate the aggregation states of metal species other than altering metal loading is desired.

Inspired by our previous work on the porous porphyrin polymers^27^ and the work of Jiang et al. on SACs derived from metal-organic frameworks^21^, we report here a precursor-dilution strategy to prepare N-doped porous carbon supported SACs. In brief, tetraphenylporphyrin (TPP) with chelated metal cations, acting as the metal precursor, is co-polymerized with excess amount of free TPP as the diluent. By the dilution, the mean distance between metal atoms dispersed on the as-prepared polymer matrix becomes sufficiently large, preventing their aggregation during the subsequent high-temperature pyrolysis. Thus, SACs are obtained. Specially, the high chelating ability of TPP to various metal cations^28–30^ (Supplementary Fig. 1) empowers this method to be applicable for fabricating a wide variety of SACs. Using this strategy, we have successfully conducted the synthesis of 24 types of SACs (i.e., M_1_/N–C, M = Ti, V, Cr, Mn, Fe, Co, Ni, Cu, Ga, Zr, Mo, Ru, Rh, Pd, Ag, Cd, In, Sn, Er, W, Ir, Pt, Au, and Bi), including noble metals and non-noble metals. Furthermore, by varying the preparation conditions, e.g., precursor concentrations, metal precursors, and pyrolysis temperatures, we can obtain various materials: SACs with different surface atom densities (0.002–0.034 Pt·nm^–2^), bi-metallic SACs (Pt_1_–Sn_1_/N–C), and Pt catalysts with different aggregation states (Pt SAs, Pt NCs, and Pt NPs), respectively.

## Results

### Synthesis of Pt SACs with the precursor-dilution strategy

**Fig. 1:**
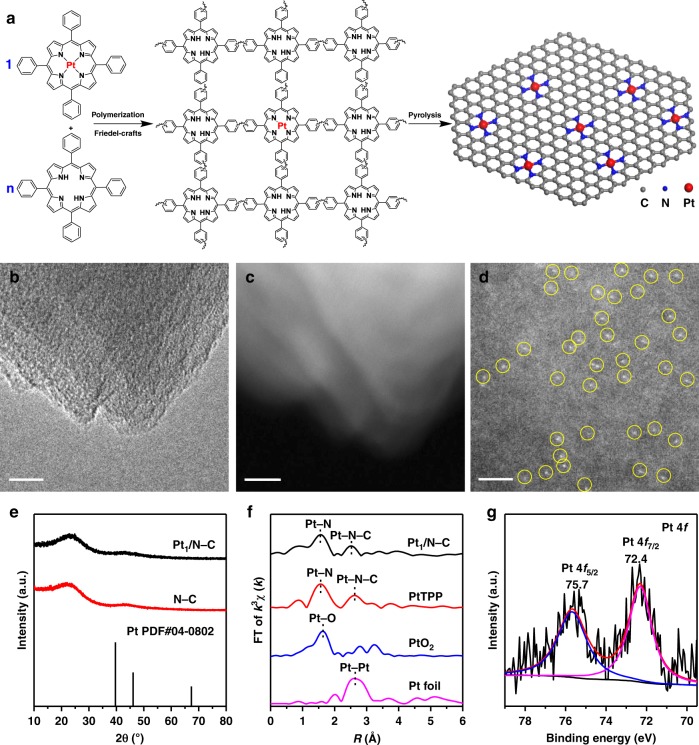
Preparation and structural characterization of Pt_1_/N–C. **a** Schematic illustration of the preparation of Pt_1_/N–C. The molar ratio of PtTPP:TPP is denoted as 1:n. **b** TEM image of Pt_1_/N–C. Scale bar, 10 nm. **c** STEM image of Pt_1_/N–C. Scale bar, 10 nm. **d** AC HAADF-STEM image of Pt_1_/N–C. SAs were highlighted by yellow circles. Scale bar, 2 nm. **e** XRD pattern of Pt_1_/N–C and N–C. **f** The k^3^-weighted R-space FT spectra of EXAFS for Pt_1_/N–C, PtTPP, PtO_2_, and Pt foil. **g** The XPS patterns of Pt 4*f* for Pt_1_/N–C

In this work, we use Pt_1_/N–C as an example to show the precursor-dilution strategy for fabricating SACs (Fig. 1a). First, a mixture of tetraphenylporphyrin platinum (PtTPP) and free TPP (PtTPP:TPP = 1:40, mol:mol) was dissolved in dichloromethane, and then co-polymerized by the addition of anhydrous AlCl_3_ (Friedel-Crafts alkylation reactions)^31^. The as-obtained polymers were treated at 600 °C under flowing N_2_ gas, and nitrogen-doped-carbon supported Pt SACs (i.e., Pt_1_/N–C) were obtained.

The transmission electron microscopy (TEM) image (Fig. 1b) and high-angle annular dark-field scanning transmission electron microscopy (STEM) image (Fig. 1c) revealed that there were no observable Pt NPs in the prepared SACs. The image taken by aberration corrected high-angle annular dark-field scanning transmission electron microscopy (AC HAADF-STEM) showed that individual Pt atoms highlighted by yellow circles in Fig. 1d were clearly visible (no bright dots can be observed in the underlying support of nitrogen-doped carbon without metal loading (i.e., N–C), Supplementary Fig. 2), resulting from the large difference in Z contrasts of the image for Pt and N/C. Thus, this image proved the presence of atomically dispersed Pt species. The X-ray diffraction (XRD) pattern of Pt_1_/N–C exhibited no peaks at 39.8°, 46.2°, and 68.5° (Fig. 1e, PDF#04-0802). This pattern resembled that for N–C and indicated the highly dispersed state of Pt species. The aggregation state of Pt species was also probed by extended X-ray absorption fine structure spectrometry (EXAFS, Fig. 1f). There were two notable peaks at 1.7 and 2.5 Å, similar to those in the spectrum of PtTPP, which can be ascribed to the Pt–N and Pt–N–C contributions^32,33^, respectively. It should be noted that the peak at 2.5 Å cannot be ascribed to the Pt–Pt bond (2.7 Å for Pt foil), which was further confirmed by the EXAFS fitting results of Pt_1_/N–C (Supplementary Fig. 3, Supplementary Table 1). These fitting results were in good agreement with the original curves, and the coordination number of the Pt with surrounding N atoms was 3.4, indicating that the Pt atoms were connected with three or four N atoms^34,35^. These results again corroborated the dominant presence of atomically dispersed Pt species evidenced by AC HAADF-STEM. As shown from the X-ray absorption near edge structure (XANES) spectra (Supplementary Fig. 4), the energy absorption threshold of Pt_1_/N–C located between Pt foil and PtO_2_, implying the presence of positively charged Pt^δ+^ stabilized by adjacent N atoms in Pt_1_/N–C. The oxidation state of Pt species was characterized by X-ray photoelectron spectroscopy (XPS, see Fig. 1g). The Pt 4*f* peaks located at 72.4 and 75.7 eV can be tentatively ascribed to Pt^2+^ with the presence of Pt–N bonds^36^. The inductively coupled plasma optical emission spectrometry (ICP-OES) analysis revealed that the actual Pt loading was 0.43 wt% (Supplementary Table 2), slightly lower than the nominal loading (0.73 wt%) estimated by the molar ratio of PtTPP:TPP (1:40). This may be caused by metal loss in the preparation process. The result of elemental analysis (EA) revealed 5.17 wt% nitrogen content of in Pt_1_/N–C (Supplementary Table 2). High BET area (595 m^2 ^g^–1^) was found for Pt_1_/N–C (Supplementary Table 2), and it was reported that high-surface-area structures could facilitate the atomic dispersion of metal species^37^.

All the characterization results above lead to a conclusion that atomically dispersed Pt species were successfully synthesized on the support of N-doped carbon, by the precursor-dilution strategy. On the contrary, when PtTPP was used to make polymers without the diluent of free TPP, e.g., under the conditions of Pt–NPs/N–C(1:0), 100% PtTPP-based polymers and 3.31 wt% Pt loading (Supplementary Table 3), Pt NPs with 3.9 nm in diameter were formed using the same synthetic scheme (Supplementary Fig. 5). Thus, the diluent is indispensable for successful fabrication of SACs.

### The versatility of the precursor-dilution strategy

The precursor-dilution strategy is of significant flexibility and generality for SAC fabrication, as demonstrated below. All of the synthesized catalysts were characterized fully by TEM, STEM, XRD, ICP-OES, EA, and BET (see Supplementary Figs. 6–28, 30–33 and Supplementary Tables 4–30). Among them, TEM/STEM images and XRD patterns were used to preliminarily identify the aggregation states of metal species on the supports. ICP-OES was used to reveal the content of metal species, and the EA and BET measurements were used to probe catalysts’ texture.

**Fig. 2:**
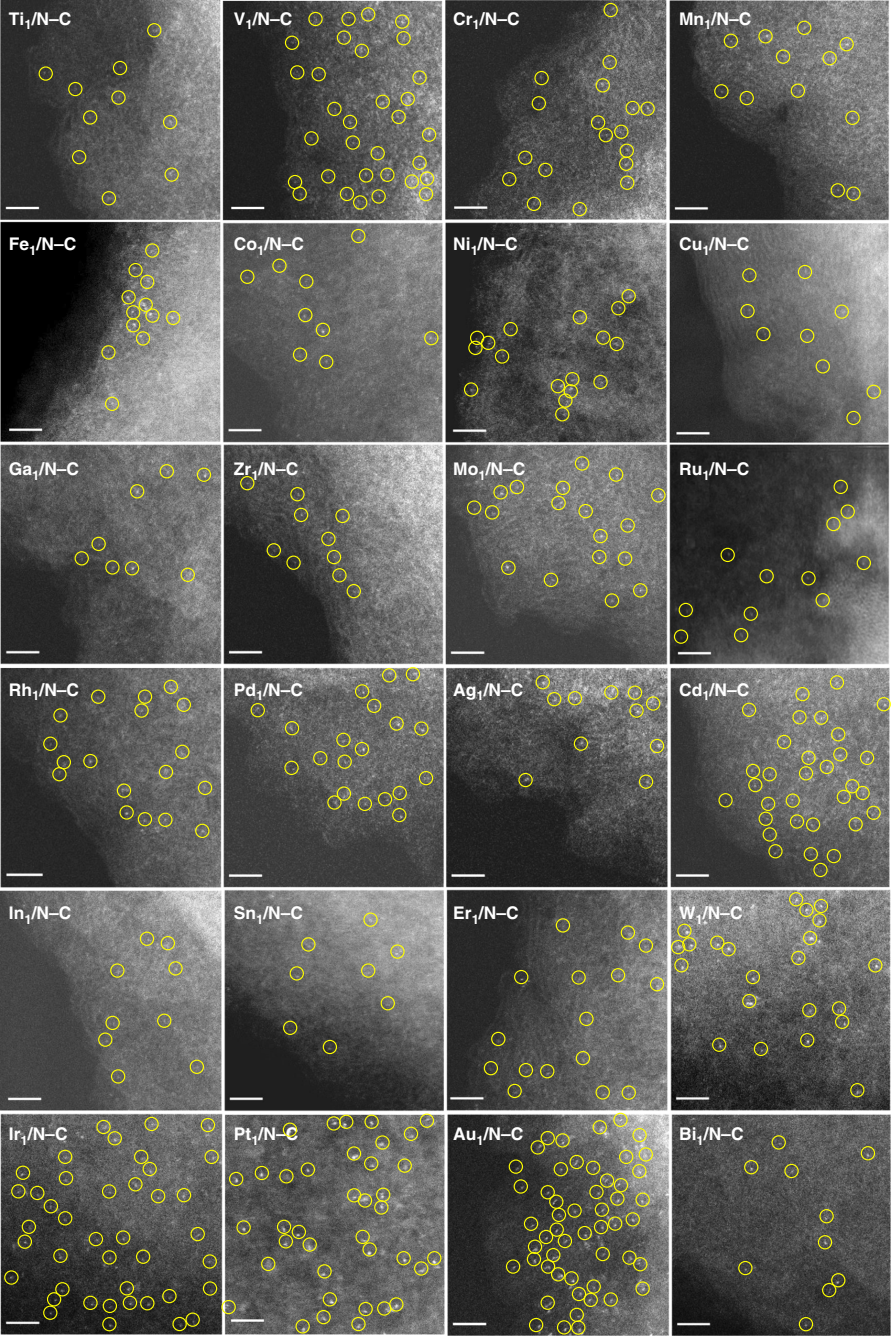
AC HAADF-STEM images of M_1_/N–C. M = Ti, V, Cr, Mn, Fe, Co, Ni, Cu, Ga, Zr, Mo, Ru, Rh, Pd, Ag, Cd, In, Sn, W, Ir, Pt, Au, and Bi. SAs were highlighted by yellow circles. Scale bar, 2 nm

First, we could extend the precursor-dilution strategy to fabricate a variety of SACs using MTPP with different metals (M = Ti, V, Cr, Mn, Fe, Co, Ni, Cu, Ga, Zr, Mo, Ru, Rh, Pd, Ag, Cd, In, Sn, Er, W, Ir, Au, and Bi) as the precursors and free TPP as the diluent. For most of the metals, the ratio of MTPP:TPP of 1:40 was used during the catalyst synthesis. But, there were exceptions. Some MTPPs (e.g., MnTPP and FeTPP) were found to easily leach in the polymerization process (under 80 °C and in AlCl_3_). Thus, the molar ratios of MTPP:TPP were increased to obtain SACs with meaningful metal loadings (>0.05 wt%). The samples with high content of Rh or Au tended to form NPs, so the molar ratios of RhTPP:TPP and AuTPP:TPP were decreased to 1:80 and 1:160, respectively, in order to obtain atomically dispersed metal species. Details of all the synthesis were provided in Supplementary Methods. AC HAADF-STEM images (Fig. 2) showed that all of the 24 SACs featured with atomically dispersed species on the supports, which were further confirmed by the corresponding EXAFS results with the absence of metal–metal bond (Supplementary Fig. 29). Among them, SACs of Cd, Bi, and Er have never been reported before, which may underpin the exploration of intriguing applications^38^. The EA and BET results revealed some similarity of material texture among all of the catalysts, i.e., with >420 m^2^ g^−1^ BET areas and ~5.0 wt% nitrogen content, due to the utilization of the similar preparation protocols.

**Fig. 3:**
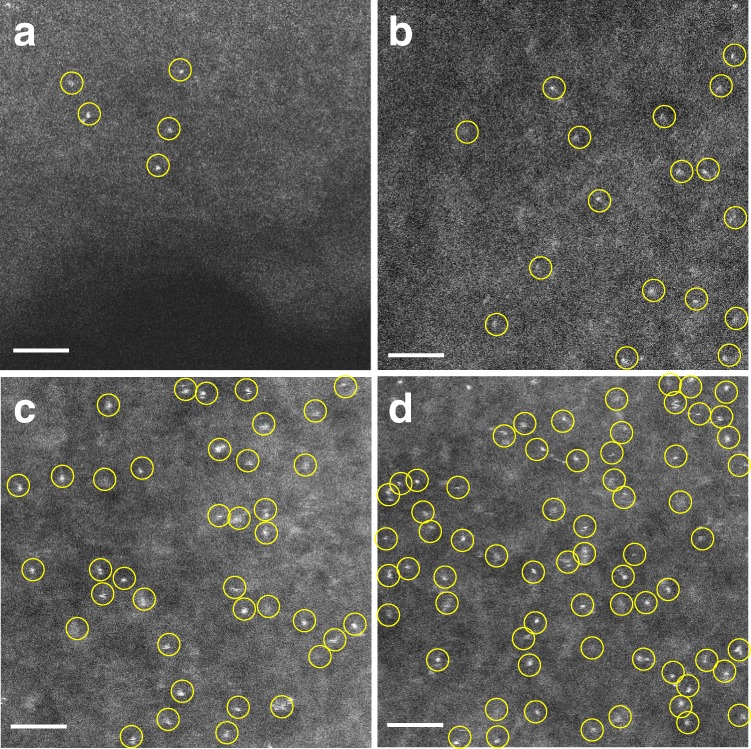
AC HAADF-STEM images of Pt SACs with different atom densities. AC HAADF-STEM images of **a** Pt_1_/N–C(1:320); **b** Pt_1_/N–C(1:80); **c** Pt_1_/N–C(1:40); and **d** Pt_1_/N–C(1: 20). SAs are highlighted by yellow circles. Scale bar, 2 nm

Second, we could tune the surface Pt atom density in Pt_1_/N–C by changing the precursor concentrations (i.e., the molar ratios of PtTPP:TPP). Using different molar ratios of PtTPP:TPP (i.e., 1:320, 1:80, 1:40, and 1:20), we prepared a set of SACs with different Pt contents (0.06, 0.21, 0.43, and 0.73 wt%, respectively) and similar BET areas (~600 m^2^ g^–1^, see Supplementary Tables 2 and 27–29), and thus different Pt surface densities (0.002, 0.010, 0.022, and 0.034 Pt·nm^–2^, respectively)^39,40^, in line with the trend observed by AC HAADF-STEM (Fig. 3).

**Fig. 4:**
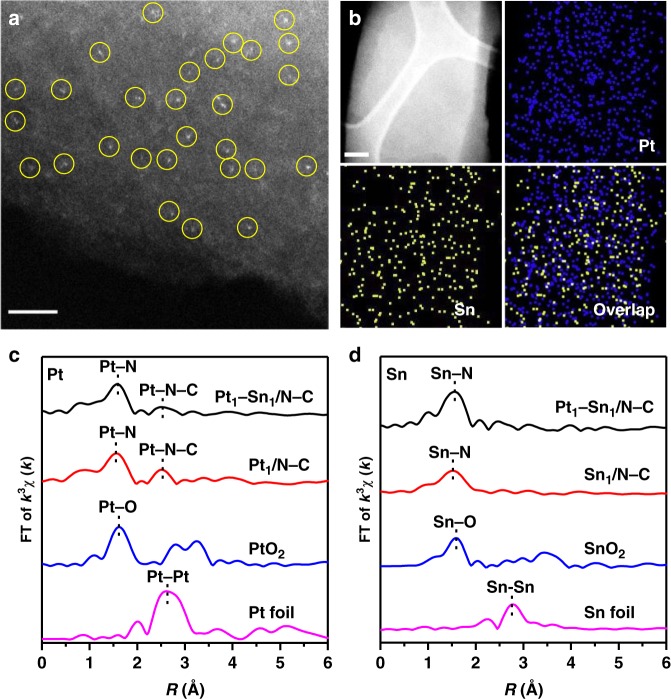
Structural characterization of Pt_1_–Sn_1_/N–C. **a** AC HAADF-STEM image of Pt_1_–Sn_1_/N–C. SAs were highlighted by yellow circles. Scale bar, 2 nm. **b** element mapping of Pt_1_–Sn_1_/N–C. Scale bar, 100 nm. **c** The Pt k^3^-weighted R-space FT spectra of EXAFS for Pt_1_–Sn_1_/N–C, Pt_1_/N–C, PtO_2_, and Pt foil. **d** The Sn k^2^-weighted R-space FT spectra of EXAFS for Pt_1_–Sn_1_/N–C, Sn_1_/N–C, SnO_2_, and Sn foil

Third, fabricating bi-metallic SACs (e.g., Pt_1_–Sn_1_/N–C) was also achieved with the same synthesis procedure of Pt_1_/N–C and the precursor molar ratio of PtTPP:SnTPP:TPP (1:1:40). The N-doped porous carbon-based materials with 0.48 wt% Pt loading and 0.35 wt% Sn loading were obtained (Supplementary Table 30). This ratio of Pt loading and Sn loading (1.4:1) was in good agreement with the nominal ratio (1.6:1), based on the molar ratio of PtTPP:SnTPP (1:1) and atomic weight ratio of Pt:Sn (195.1:118.7). The AC HAADF-STEM image for Pt_1_–Sn_1_/N–C revealed the metal species atomically dispersed on the porous carbon supports (Fig. 4a). Corresponding element mapping analysis of Pt_1_–Sn_1_/N–C revealed that both Pt and Sn species were homogeneously distributed (Fig. 4b). The results of EXAFS (no Pt–Pt bond and Sn–Sn bond, Fig. 4c, d) were also indicative of the dominant presence of isolated Pt and Sn atoms deposited on the carbon matrix. These mutually authenticated results provided compelling evidence for the preparation of Pt_1_–Sn_1_/N–C.

**Fig. 5:**
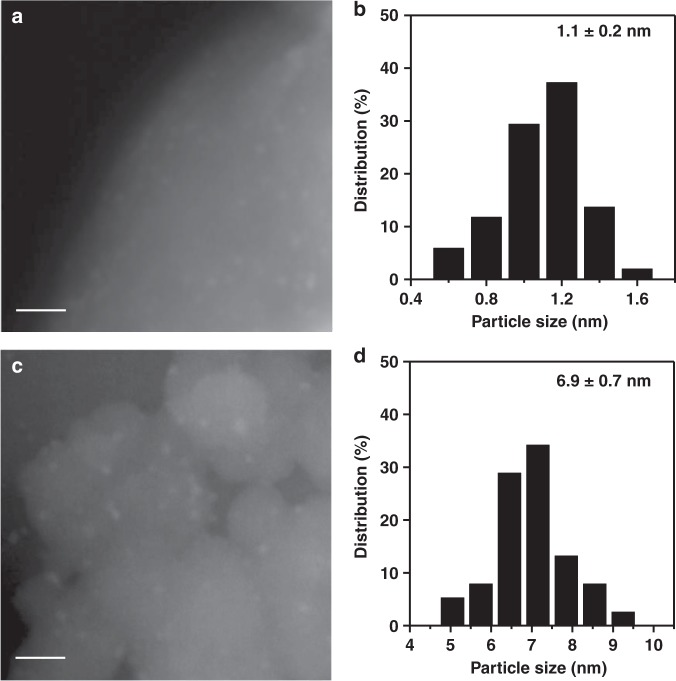
Structural characterization of Pt–NCs/N–C and Pt–NPs/N–C. **a** STEM image of Pt–NCs/N–C. Scale bar, 10 nm. **b** Particle size distribution of Pt–NCs/N–C. **c** STEM image of Pt–NPs/N–C. Scale bar, 50 nm. **d** Particle size distribution of Pt–NPs/N–C

Forth, we found that the pyrolysis temperature during the materials fabrication could influence the aggregation states of dispersed metal atoms. When the samples with the same molar ratio of PtTPP:TPP (1:40) were treated in different pyrolysis temperatures (i.e., 600, 700, and 800 °C), the Pt contents and BET surface areas of them were close (~0.5 wt% and ~600 m^2^ g^–1^, respectively, see Supplementary Tables 2 and 31–32), while the aggregation states of Pt species were changed from SAs (Pt_1_/N–C) to NCs (Pt–NCs/N–C, 1.1 nm) and NPs (Pt–NPs/N–C, 6.9 nm) (Fig. 1d, Fig. 5 and Supplementary Figs. 34–35).

### The chemo-/regio-selectivity of Pt SACs in hydrogenation

**Fig. 6:**
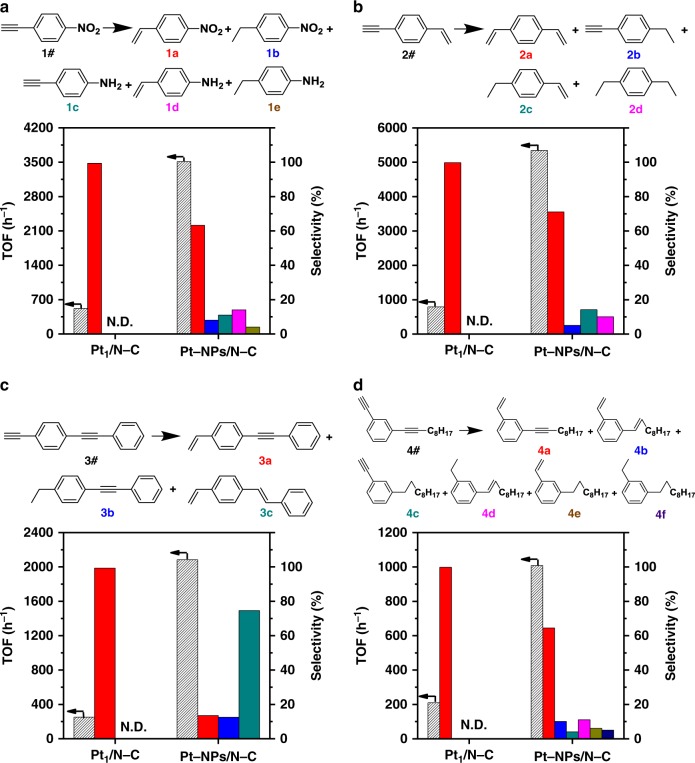
Catalytic performance of Pt_1_/N–C and Pt–NPs/N–C. Reaction results for the hydrogenation of **a** 1-nitro-4-ethynylbenzene, **b** 1-ethynyl-4-vinylbenzene, **c** 1-ethynyl-4-(phenylethynyl)benzene, **d** 1-(dec-1-yn-1-yl)-3-ethynylbenzene on Pt_1_/N–C and Pt–NPs/N–C. Reaction condition: substrate (0.5 mmol), catalyst (Pt:substrate = 1:1200, mol:mol), methanol (2.0 mL), H_2_ (1.0 MPa), 50 °C (**a**, **b**) or 80 °C (**c**, **d**). All the conversions were maintained at ~20%. TOF was calculated based on Pt dispersion (Pt_1_/N–C: 100%; Pt–NPs/N–C: 14.5%, estimated by particle size (6.9 nm) according to *D* *=* 1/*d*_Pt_)

After illustrating the facile synthetic routes of SACs with great versatility, we show here the unique catalytic properties of SACs (Pt_1_/N–C, with 0.43 wt% Pt loading) compared with NPs (Pt–NPs/N–C, with Pt–NPs of 6.9 nm in diameter and 0.52 wt% Pt loading) in hydrogenation reactions, which were previously illustrated as a promising solution in practical applications of SACs^1^. To our great delight, Pt_1_/N–C showed excellent chemoselectivity in the hydrogenation of 1-nitro-4-ethynylbenzene (with –C≡CH and –NO_2_) and 1-ethynyl-4-vinylbenzene (with –C≡CH and –C=CH_2_), as it only transformed alkyne groups to alkenyl groups and kept –NO_2_ and –C=CH_2_ intact (99% selectivity to 1-nitro-4-vinylbenzene and 99% selectivity to 1,4-divinylbenzene at ~20% conversion level, and 98% selectivity to 1-nitro-4-vinylbenzene and 97% selectivity to 1,4-divinylbenzene at ~100% conversion level, respectively, Fig. 6a, b and Supplementary Fig. 36a, b). In contrast, similar catalysis on Pt–NPs/N–C induced the formation of multiple products, resulting from the co-hydrogenation of –C≡CH and –NO_2_, and –C≡CH and –C=C, respectively. The Pt_1_/N–C catalyst permits the distinction between –C≡CH and –NO_2_/–C=C in hydrogenation mainly because of the good match between the relatively low catalytic activity of Pt SACs and high reactivity of terminal alkynes^41^.

More inspiringly, our Pt_1_/N–C showed rarely reported regioselectivity in the hydrogenation of 1-ethynyl-4-(phenylethynyl)benzene and 1-(dec-1-yn-1-yl)-3-ethynylbenzene (with –C≡CH and –C≡C–), as it only converted terminal alkyne to alkenyl while kept internal alkyne intact: 99% selectivity to 1-(phenylethynyl)-4-vinylbenzene and 99% selectivity to 1-(dec-1-yn-1-yl)-3-vinylbenzene at ~20% conversion level, and 98% selectivity to 1-(phenylethynyl)-4-vinylbenzene and 97% selectivity to 1-(dec-1-yn-1-yl)-3-vinylbenzene at ~100% conversion level, respectively (Fig. 6c, d, Supplementary Figs. 36c and 36d). However, similar hydrogenation reactions catalyzed by Pt–NPs/N–C were not selective, i.e., both terminal and internal alkynes were hydrogenated. For example, the hydrogenation of 1-ethynyl-4-(phenylethynyl)benzene catalyzed by Pt–NPs/N–C showed 13%, 12%, and 75% selectivities to 1-(phenylethynyl)-4-vinylbenzene, 1-ethyl-4-(phenylethynyl)benzene, and 1-styryl-4-vinylbenzene, respectively (Fig. 6c).

It is generally accepted that there are two activation pathways for semi-hydrogenation of alkyne: (i) the terminal of the –C≡CH group interacts with metal surfaces leading to deprotonation, and then the –C≡C group becomes activated; (ii) the entire –C≡C group interacts with the metal surfaces and becomes activated^42^. In our system, because of possible steric hindrance effect (1.2 Å for C≡C bond length vs. 0.8 Å for Pt^2+^ radius^43^) of Pt_1_/N–C, the first pathway is more probable. Apparently, due to its absence of terminal hydrogen, internal alkyne cannot be activated and then hydrogenated on Pt_1_/N–C. On the contrary, Pt–NPs/N–C with much larger diameters than that of Pt_1_/N–C are able to interact with substrates with less steric hindrance effect^44^ and then catalyze the hydrogenation of both terminal and internal alkynes. To verify our assumption, Pt_1_/N–C, Pt–NCs/N–C (1.1 nm), and Pt–NPs/N–C (6.9 nm) were employed under the same reaction conditions (Supplementary Table 33). As expected, the catalytic activities (i.e., turnover frequency, TOF, based on the metal dispersion^13^) for the hydrogenation of internal alkynes on Pt–NCs/N–C fell between those on Pt_1_/N–C and Pt–NPs/N–C: 1-phenyl-1-propyne (0, 132, and 2946 h^−1^), 1-phenyl-1-pentyne (0, 93, and 2556 h^−1^), and 5-decyne (0, 2860, and 13300 h^−1^) on SACs, NCs, and NPs, respectively. The observation that the activities for the hydrogenation of internal alkynes increased with the increasing size of Pt species coincided quite well with our speculation that the unique group discrimination of terminal alkynes from internal ones on Pt_1_/N–C can be attributed to the geometric effect (see Supplementary Fig. 37).

In addition, the stability of the Pt_1_/N–C catalysts in the hydrogenation of the four substrates, i.e., 1-nitro-4-ethynylbenzene, 1-ethynyl-4-vinylbenzene, 1-ethynyl-4-(phenylethynyl)benzene, and 1-(dec-1-yn-1-yl)-3-ethynylbenzene, respectively, was evaluated. As shown in Supplementary Fig. 38, recycling Pt_1_/N–C catalysts for five runs exhibited no essential decrease in catalytic activities and selectivities (~98%). Furthermore, no Pt nanoparticles or nanoclusters were found in TEM and STEM images (Supplementary Fig. 39), and the corresponding AC HAADF-STEM images revealed that the Pt species maintained the atomically dispersed states after five catalytic runs. These results above suggested that the Pt_1_/N–C catalysts exhibited excellent recyclability under the aforementioned reaction conditions.

## Discussion

In summary, a precursor-dilution strategy was developed to synthesize a series of SACs on N-doped porous carbon supports. This strategy is facile and versatile, and thus meets the requirements of the in-depth research nowadays. The Pt_1_/N–C SACs prepared with this strategy showed extremely high chemo- and regioselectivity towards terminal alkynes in hydrogenation. These findings are of significant importance in broadening the application of SACs, with the implication that SACs are able to achieve superior selectivity comparable to homogeneous catalysts and enzyme catalysts, for the catalysis of complex molecules.

## Methods

### Catalyst preparation

Take Pt_1_/N–C as example. Under a nitrogen atmosphere, 100 mL stainless batch tank reactor was charged with a solution of PtTPP (0.038 mmol), TPP (1.500 mmol), and anhydrous AlCl_3_ (24 mmol) in 30 mL of dichloromethane (PtTPP:TPP = 1:40, mol:mol). The reaction mixture was stirred at 80 °C for 24 h and then cooled to room temperature. The as-obtained precipitate was filtered and washed with methanol, dichloromethane, tetrahydrofuran, N,N-dimethylformamide, and acetone, respectively. Subsequently, the resulted polymer was further purified by Soxhlet extractions for 24 h with methanol and dichloromethane, respectively. After dried at 80 °C in vacuum for 24 h, the polymer was placed in a tube furnace, heated to 600 °C for 3 h at the heating rate of 5 °C min^−1^ under flowing nitrogen gas and then naturally cooled to room temperature to obtain Pt_1_/N–C. Detailed preparation conditions for other samples were described in Supplementary Methods.

### Characterization

EA and ICP-OES were performed on Vario EL cube instrument and PerkinElmer OPTIMA 8000DV, respectively. BET surface areas measurements were performed on a Micromeritic ASAP2020M analyzer at liquid nitrogen temperature. Before the measurement, samples were evacuated at 200 °C for 6 h. Specific surface areas were calculated based on the BET equation. The Pt surface density was calculated by the equation: Pt surface density = [Pt loading] × *N*_A_/(195.08 × [surface area]), where *N*_A_ is the Avogadro’s number, and Pt loading and the surface area were obtained by the measurement of ICP-OES and BET, respectively. XRD patterns were obtained on a Bruker D8 Advanced diffractometer in the 2*θ* range 10–80°. XPS measurements were performed on an ESCALab250 XPS system with Al Kα source and a charge neutralizer, and the binding energies were referenced to the contaminated C 1*s* (284.8 eV). TEM images and STEM images were obtained on FEI Tecnai G2 F30 operated at 300 kV. AC HAADF-STEM images were obtained on a JEM-ARM200F transmission electron microscopy operated at 200 kV, which incorporated with double spherical aberration correctors. X-ray absorption spectroscopy (XAS) measurements were conducted on BL14W beamline at the Shanghai Synchrotron Radiation Facility (SSRF) and 1W1B beamline at the Beijing Synchrotron Radiation Facility (BSRF). The sample of SACs were measured in fluorescence mode using Lytle detector or 32-element Ge solid state detector and the corresponding metal foils and metal oxides were used as reference samples and measured in the transmission mode. ^1^H and ^13^C nuclear magnetic resonance (NMR) spectrums were obtained on Bruker Avance III 500 HD. High-resolution mass spectral (HRMS) data were obtained on Thermo Fisher Scientific Tribrid Mass Spectrometer (Orbitap Fusion Lumos).

### Catalytic performance test

Catalytic hydrogenation reactions of various substrates, including 1-phenyl-1-propyne, 1-phenyl-1-pentyne, 5-decyne, 1-nitro-4-ethynylbenzene, 1-ethynyl-4-vinylbenzene, 1-ethynyl-4-(phenylethynyl)benzene, and 1-(dec-1-yn-1-yl)-3-ethynylbenzene, were carried out in 10 mL stainless autoclave. Take hydrogenation of 1-nitro-4-ethynylbenzene on Pt_1_/N–C as example. The typical reaction conditions were 0.5 mmol 1-nitro-4-ethynylbenzene, 20 mg Pt_1_/N–C catalyst (0.43 wt%, Pt:1-nitro-4-ethynylbenzene = 1:1200, mol:mol), 2.0 mL methanol as solvent, 1.0 MPa H_2_ and 50 °C. After cooled and filtered, the reaction products were analyzed by GC (Shimadzu 2010 GC Plus) and GC-MS (Shimadzu GCMS-QP2010 Ultra). Selectivities were reported on a carbon basis, and TOF as molar substrate conversion rates per mole of surface Pt atoms per hour (h^−1^). For Pt_1_/N–C, the Pt dispersion was estimated to be 100%; for Pt–NCs and Pt–NPs, the dispersion (*D*) was estimated by the metal particle size (*d*) according to *D* = 1/*d*_pt_, respectively. Detailed reaction conditions were given in the footnotes of Fig. 6 and Supplementary Table 33. Corresponding ^1^H and ^13^C NMR spectrums of some uncommon substrates and products, including 1-ethynyl-4-(phenylethynyl)benzene (3#), 1-(phenylethynyl)-4-vinylbenzene (3a), 1-ethyl-4-(phenylethynyl)benzene (3b), 1-styryl-4-vinylbenzene (3c), 1-(dec-1-yn-1-yl)-3-ethynylbenzene (4#), 1-(dec-1-yn-1-yl)-3-vinylbenzene (4a), 1-(dec-1-en-1-yl)-3-vinylbenzene (4b), 1-decyl-3-ethynylbenzene (4c), 1-(dec-1-en-1-yl)-3-ethylbenzene (4d), 1-decyl-3-vinylbenzene (4e), and 1-decyl-3-ethylbenzene (4f) were showed in Supplementary Figs. 40–61, respectively.

## Supplementary information

Supplementary Information

Peer Review

Source Data

## Data Availability

The data underlying Figs. 1 and 4–6, Supplementary Figs. 3–36 and 38 are provided as a Source Data file. The other data that support the findings of this study are available from the corresponding author upon request.
