# Supplementary material for: A versatile route to fabricate single atom catalysts with high chemoselectivity and regioselectivity in hydrogenation
Source: Nat Commun. 2019 Aug 14;10:3663. doi: 10.1038/s41467-019-11619-6 (PMC6694111; doi:10.1038/s41467-019-11619-6)
Supplement: Supplementary file 1 — Supplementary Information [file 41467_2019_11619_MOESM1_ESM.pdf]

Supplementary Information for

A versatile route to fabricate single atom catalysts with high chemoselectivity and regioselectivity in hydrogenation

He et al.

Supplementary Methods

Materials. All the solvents and chemicals were available from suppliers and used as received unless specially stated.

Dichloromethane (Greagent, 99.5%), hexane (Greagent, 97%), methanol (Greagent, 99.5%), propionic acid (Aladdin, 99.5%), benzaldehyde (Aladdin, 99.5%), *N*,*N*-dimethylformamide (Aladdin, 99.5%), toluene (ACS reagent, 99.5%), acetic acid (Aladdin, 99.5%), 1,2,4-trichlorobenzene (Aladdin, 99.5%), tetrahydrofuran (Aladdin, 99.5%), triethylamine (Aladdin, 99.5%), n-butyllithium solution (Aladdin, 2.5 M in hexanes), benzonitrile (Aladdin, 99%), phenol (Aladdin, 99.5%), decahydronaphthalene (Aladdin, 98%), diphenyl ether (Aladdin, 99.5%), 1-phenyl-1-propyne (Aladdin, 98%), 1-phenyl-1-pentyne (TCI, 97%), 5-decyne (Aladdin, 98%), 1-nitro-4-ethynylbenzene (TCI, 98%), 1-ethynyl-4-vinylbenzene (Chengdu Novel Biochemical Co. Ltd, 98%), 1-ethynyl-4-(phenylethynyl)benzene (Chengdu Novel Biochemical Co. Ltd, 98%), 1-(dec-1-yn-1-yl)-3-ethynylbenzene (Chengdu Novel Biochemical Co. Ltd, 97%). Pyrrole (Aladdin, 99%) was distilled under a N_2 atmosphere before use.

Sodium acetate anhydrous (CH_3COONa, Aladdin, 99%), titanium(IV) chloride solution ($TiCl_4$, J&K, 1.0 M solution in toluene), vanadyl sulfate hydrate ($VOSO_4 \cdot xH_2O$, Sigma-Aldrich, 97%), chromium chloride ($CrCl_2$, Aladdin, 99%), manganese chloride tetrahydrate ($MnCl_2 \cdot 4H_2O$, Aladdin, 99%), iron(II) chloride ($FeCl_2$, Aladdin, 99.5%), cobalt(II) acetate tetrahydrate ($Co(CH_3COO)_2 \cdot 4H_2O$, Aladdin, 99.5%), nickel(II) acetate tetrahydrate ($Ni(CH_3COO)_2 \cdot 4H_2O$, Aladdin, 99%), copper(II) acetate monohydrate ($Cu(CH_3COO)_2 \cdot H_2O$, Aladdin, 99%), gallium(III) chloride anhydrous ($GaCl_3$, Aladdin, 99.99%), zirconyl chloride octahydrate ($ZrOCl_2 \cdot 8H_2O$, Aladdin, 99%), molybdenum

trioxide (MoO_3, Aladdin, 99.5%), triruthenium dodecacarbonyl ($Ru_3C_{12}O_{12}$, Aladdin, 99%), di-μ-chloro-tetracarbonyldirhodium(I) ($[Rh(CO)_2Cl]_2$, Aladdin, 97%), palladium chloride ($PdCl_2$, Aladdin , Pd 59–60%), silver nitrate ($AgNO_3$, Aladdin, 99.8%), cadmium acetate dihydrate ($Cd(CH_3COO)_2 \cdot 2H_2O$, Aladdin, 99.5%), indium chloride tetrahydrate ($InCl_3 \cdot 4H_2O$, Aladdin, 99.9%), tin chloride ($SnCl_2$, Aladdin, 99%), erbium(III) acetylacetonate hydrate ($Er(C_5H_7O_2)_3 \cdot xH_2O$, Sigma, 97%), tungsten chloride (WCl_6, Aladdin, 99.9%), chloro(1,5-cyclooctadiene)iridium(I) dimer ($C_{16}H_{24} \cdot Ir_2Cl_2$, Aladdin, 97%), platinum chloride ($PtCl_2$, Aladdin, Pt≥73%), gold chloride trihydrate ($HAuCl_4 \cdot 3H_2O$, Aladdin, 99.9%), bismuth nitrate pentahydrate ($Bi(NO_3)_3 \cdot 5H_2O$, Aladdin, 98%), silica gel (Qingdao Haiyang Chemical Plant, 200–300 mesh), aluminum oxide (Aladdin, 200–300 mesh).

Synthesis of TPP and MTPP. TPP[1], TiTPP[2], VTPP[3], CrTPP[4], MnTPP[1], FeTPP[1], CoTPP[1], NiTPP[5], CuTPP[6], GaTPP[4], ZrTPP[7], MoTPP[8], RuTPP[9], RhTPP[10], PdTPP[11], AgTPP[12], CdTPP[13], InTPP[14], SnTPP[15], ErTPP[16], WTPP[17], IrTPP[18], PtTPP[19], AuTPP[20], and BiTPP[21] were synthesized according to the reports, respectively. Details were given in the following:

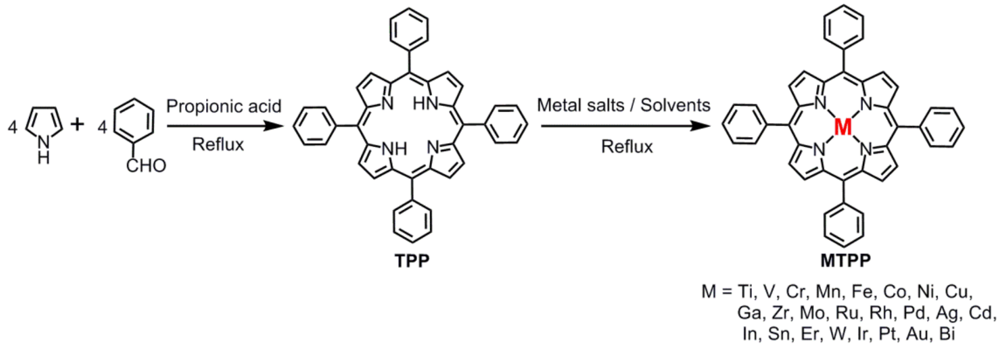

Supplementary Fig. 1 Schematic illustration of the preparation of TPP and MTPP.

TPP: Typically, benzaldehyde (100 mmol) was added to a two-necked round-bottomed flask (500 mL) containing 250 mL propionic acid, and heated to 140 ℃, followed by addition of freshly distilled pyrrole (100 mmol). After refluxing for 3 h, the solution was cooled to room temperature naturally and added 250 mL absolute ethanol. Subsequently, the as-obtained precipitate was filtered, washed with methanol and dried in the air. The precipitate was further purified with silica gel column chromatography using CH_2Cl_2 as eluent. After removal of eluent by rotary evaporation, the product was dried at 80 ^{o}C in vacuum for 24 h and gave the purple powder with the yield of 23%. ^{1}H NMR (500 MHz, $CDCl_3$): δ 8.84 (s, 8H), 8.23–8.21 (d, J = 8.22 Hz, 8H), 7.80–7.73 (m, 12H), -2.78 (s, 2H).

TiTPP: Under the N_2 atmosphere, the as-synthesized TPP (1.0 mmol) was dissolved in 100 mL dry toluene in a 250 mL Schlenk flask and carefully syringed 5 mL n-butyllithium solution (2.5 M in hexanes), followed by stirred for 30 min at room temperature. Then 25 mL titanium(IV) chloride solution ($TiCl_4$, 1.0 M solution in toluene) was added by syringe, and stirred for 3 h at 100 ℃. The solution was cooled to room temperature naturally, then exposed to air and stirred for 3 h. After removal of solvent by rotary evaporation, the material was further purified by silica gel column chromatography with CH_2Cl_2 as eluent. The eluent was removed by rotary evaporation and the product was dried at 80 ^{o}C in vacuum for 24 h.

VTPP: Under the N_2 atmosphere, the as-synthesized TPP (1.0 mmol) and vanadyl sulfate hydrate (10.0 mmol) were dissolved in 100 mL N,N-dimethylformamide (DMF) in a 250 mL three-necked round-bottomed flask, and refluxed for 3 h at 150 ℃, then the solution was cooled to room temperature naturally. After removal of DMF solvent by

rotary evaporation, the material was purified by silica gel column chromatography with CH_2Cl_2/hexanes/MeOH as eluents. The eluents were removed by rotary evaporation and the product was dried at 80 ^oC in vacuum for 24 h.

Similarly, MTPP (M = Cr, Mn, Fe, Co, Ni, Cu, Ga, Pd, Ag, Cd, In, Sn, Er, and Bi) were also synthesized with the same procedure of VTPP and different metal salts (chromium chloride, manganese chloride tetrahydrate, iron(II) chloride, cobalt(II) acetate tetrahydrate, nickel(II) acetate tetrahydrate, copper(II) acetate monohydrate, gallium(III) chloride anhydrous, palladium chloride, silver nitrate, cadmium acetate dehydrate, indium chloride tetrahydrate, tin chloride, erbium(III) acetylacetonate hydrate, bismuth nitrate pentahydrate), respectively.

Besides, MTPP (M = Zr, Mo, Ru, Rh, W, Ir, and Pt) were also synthesized with the same procedure of VTPP and different metal salts and solvents, respectively. Details were given in the following:

For ZrTPP: zirconyl chloride octahydrate, benzonitrile.

For MoTPP: molybdenum trioxide, phenol.

For RuTPP: molybdenum trioxide, decahydronaphthalene.

For RhTPP: di-μ-chloro-tetracarbonyldirhodium(I), dry toluene.

For WTPP: tungsten chloride, phenol.

For IrTPP: chloro(1,5-cyclooctadiene)iridium(I) dimer, 1,2,4-trichlorobenzene.

For PtTPP: platinum chloride, diphenyl ether.

AuTPP: Under the N_2 atmosphere, the as-synthesized TPP (1.0 mmol), gold chloride trihydrate (10.0 mmol) and sodium acetate anhydrous were dissolved in 100 mL acetic acid in a 250 mL three-necked round-bottomed flask, and refluxed for 12 h at 120 ℃, then the

solution was cooled to room temperature naturally. The solvent was removed by rotary evaporation and CH_2Cl_2 was added. After washing with 10% aqurous Na_2CO_3 and water, the solution was dried by $MgSO_4$ and removed the solvent by rotary evaporation. Subsequently, the as-obtained material was further purified by silica gel column chromatography with CH_2Cl_2/hexanes/MeOH as eluents. The eluents was removed by rotary evaporation and the product was dried at 80 ^{o}C in vacuum for 24 h.

Synthesis of M_1/N-C, Pt-NPs/N-C(1:0), N-C, and Pt_1-Sn_1/N-C. A series of M_1/N-C, Pt-NPs/N-C(1:0), N-C, and Pt_1-Sn_1/N-C catalysts were prepared with similar procedure of Pt_1/N-C, and the detailed precursors ratios (MTPP:TPP, mol:mol) were also given in the following.

For M_1/N-C (M = Mn, Fe, Co, In, Er), MTPP:TPP = 1:0;

For M_1/N-C (M = Ni, Ga, Zr), MTPP:TPP = 1:10;

For M_1/N-C (M = Ag, Bi), MTPP:TPP = 1:20;

For M_1/N-C (M = Ti, V, Cr, Cu, Mo, Ru, Pd, Cd, Sn, W, Ir, Pt), MTPP:TPP = 1:40;

For M_1/N-C (M = Rh), MTPP:TPP = 1:80;

For M_1/N-C (M = Au), MTPP:TPP = 1:160;

For Pt_1/N-C (1:320), PtTPP:TPP = 1:320;

For Pt_1/N-C (1:80), PtTPP:TPP = 1:80;

For Pt_1/N-C (1:20), PtTPP:TPP = 1:20;

For Pt-NPs/N-C (1:0), PtTPP:TPP = 1:0;

For N-C, MTPP:TPP = 0:1;

For Pt_1-Sn_1/N-C, PtTPP:SnTPP:TPP = 1:1:40.

Synthesis of Pt-NCs/N-C and Pt-NPs/N-C. The preparation processes for Pt-NCs/N-C and Pt-NPs/N-C were the same as that for Pt_1/N-C except the pyrolysis temperatures that Pt-NCs/N-C and Pt-NPs/N-C were obtained by treating the as-prepared polymers under flowing nitrogen gas at 700 ℃ and 800 ℃ for 3 h, respectively.

1-ethynyl-4-(phenylethynyl)benzene 3# (Supplementary Figs. 40–41): ^{1}H NMR (500 MHz, $CDCl_3$): δ 7.55–7.52 (m, 2H), 7.50–7.46 (m, 4H), 7.38–7.35 (m, 3H), 3.18 (s, 1H); ^{13}C NMR (125 MHz, $CDCl_3$): δ 132.2, 131.8, 131.6, 128.7, 128.5, 123.9, 123.1, 122.0, 91.5, 89.0, 83.4, 79.0; HRMS (m/z): $[M+H]^+$ calcd. for $C_{16}H_{11}$, 203.0855; found, 203.0854.

1-(phenylethynyl)-4-vinylbenzene 3a (Supplementary Figs. 42–43): ^{1}H NMR (500 MHz, $CDCl_3$): δ 7.55–7.52 (m, 2H), 7.51–7.49 (d, J = 7.49 Hz, 2H), 7.40–7.39 (d, J = 7.40 Hz, 2H), 7.37–7.33 (m, 3H), 6.75–6.69 (m, 1H), 5.81–5.77 (d, J = 5.79 Hz, 1H), 5.32–5.29 (d, J = 5.30 Hz, 1H); ^{13}C NMR (125 MHz, $CDCl_3$): δ 137.6, 136.4, 131.9, 131.7, 128.5, 128.4, 126.3, 123.4, 122.7, 114.9, 90.2, 89.6; HRMS (m/z): $[M+H]^+$ calcd. for $C_{16}H_{13}$, 205.1011; found, 205.1011.

1-ethyl-4-(phenylethynyl)benzene 3b (Supplementary Figs. 44–45): ^{1}H NMR (500 MHz, $CDCl_3$): δ 7.58–7.56 (m, 2H), 7.51–7.49 (d, J = 7.50 Hz, 2H), 7.39–7.33 (m, 3H), 7.22–7.21 (d, J = 7.21 Hz, 2H), 2.72–2.67 (m, 2H), 1.30–1.26 (m, 3H); ^{13}C NMR (125 MHz, $CDCl_3$): δ 144.8, 131.7, 131.7, 128.4, 128.2, 128.0, 123.6, 120.6, 89.7, 88.9, 29.0, 15.5; HRMS (m/z): $[M+H]^+$ calcd. for $C_{16}H_{15}$, 207.1168; found, 207.1168.

1-styryl-4-vinylbenzene 3c (Supplementary Figs. 46–47): ^{1}H NMR (500 MHz, $CDCl_3$): δ 7.54–7.52 (d, J = 7.53 Hz, 2H), 7.50–7.48 (d, J = 7.49 Hz, 2H), 7.43–7.41 (d, J = 7.42 Hz, 2H), 7.39–7.36 (m, 2H), 7.29–7.27 (m, 1H), 7.15–7.08 (m, 2H), 6.76–6.71 (m,

1H), 5.80–5.76 (d, J = 5.78 Hz, 1H), 5.28–5.26 (d, J = 5.27 Hz, 1H); ^{13}C NMR (125 MHz, $CDCl_3$): δ 137.4, 137.0, 137.0, 136.6, 128.8, 128.8, 128.4, 127.8, 126.8, 126.7, 126.6, 113.9; HRMS (m/z): $[M+H]^+$ calcd. for $C_{16}H_{15}$, 207.1168; found, 207.1169.

1-(dec-1-yn-1-yl)-3-ethynylbenzene 4# (Supplementary Figs. 48–49): ^{1}H NMR (500 MHz, $CDCl_3$): δ 7.53 (s, 1H), 7.39–7.36 (m, 2H), 7.25–7.22 (m, 1H), 3.06 (s, 1H), 2.41–2.38 (m, 2H), 1.63–1.57 (m, 2H), 1.46–1.43 (m, 2H), 1.32–1.30 (m, 8H), 0.91–0.89 (m, 3H); ^{13}C NMR (125 MHz, $CDCl_3$): δ 135.2, 132.0, 131.2, 128.4, 124.6, 122.4, 91.5, 83.1, 79.8, 77.6, 32.0, 29.4, 29.3, 29.1, 28.8, 22.8, 19.5, 14.3; HRMS (m/z): $[M+H]^+$ calcd. for $C_{18}H_{23}$, 239.1794; found, 239.1793.

1-(dec-1-yn-1-yl)-3-vinylbenzene 4a (Supplementary Figs. 50–51): ^{1}H NMR (500 MHz, $CDCl_3$): δ 7.44 (s, 1H), 7.31–7.27 (m, 2H), 7.25–7.22 (m, 1H), 6.69–6.63 (m, 1H), 5.76–5.73 (d, J = 5.75 Hz, 1H), 5.27–5.24 (d, J = 5.25 Hz, 1H), 2.42–2.39 (m, 2H), 1.64–1.58 (m, 2H), 1.48–1.42 (m, 2H), 1.34–1.26 (m, 8H), 0.90–0.87 (m, 3H); ^{13}C NMR (125 MHz, $CDCl_3$): δ 137.7, 136.4, 131.0, 129.5, 128.5, 125.5, 124.5, 114.5, 90.7, 80.5, 32.0, 29.4, 29.3, 29.1, 28.9, 22.8, 19.6, 14.3; HRMS (m/z): $[M+H]^+$ calcd. for $C_{18}H_{25}$, 241.1951; found, 241.1949.

1-(dec-1-en-1-yl)-3-vinylbenzene 4b (Supplementary Figs. 52–53): ^{1}H NMR (500 MHz, $CDCl_3$): δ 7.43 (s, 1H), 7.37–7.30 (m, 3H), 6.80–6.73 (m, 1H), 6.45–6.41 (d, J = 6.43 Hz, 1H), 6.33–6.26 (m, 1H), 5.83–5.79 (d, J = 5.81 Hz, 1H), 5.31–5.28 (d, J = 5.29 Hz, 1H), 2.29–2.24 (m, 2H), 1.56–1.49 (m, 2H), 1.39–1.35 (m, 10H), 0.97–0.94 (m, 3H); ^{13}C NMR (125 MHz, $CDCl_3$): δ 138.3, 137.8, 137.0, 131.7, 129.6, 128.8, 125.5, 124.7, 124.1, 113.9, 33.2, 32.1, 29.7, 29.5, 29.5, 29.4, 22.8, 14.3; HRMS (m/z): $[M+H]^+$ calcd. for $C_{18}H_{27}$, 243.2107; found, 243.2106.

1-decyl-3-ethynylbenzene 4c (Supplementary Figs. 54–55): ^{1}H NMR (500 MHz, $CDCl_3$): δ 7.32–7.30 (m, 2H), 7.25–7.21 (m, 1H), 7.18–7.15 (m, 1H), 3.05 (s, 1H), 2.61–2.56 (m, 2H), 1.63–1.57 (m, 2H), 1.39–1.26 (m, 14H), 0.94–0.86 (m, 3H); ^{13}C NMR (125 MHz, $CDCl_3$): δ 143.2, 132.2, 129.6, 129.2, 128.3, 122.0, 84.1, 76.8, 35.8, 32.1, 31.4, 29.9, 29.7, 29.6, 29.5, 29.4, 22.8, 14.3; HRMS (m/z): $[M+H]^+$ calcd. for $C_{18}H_{27}$, 243.2107; found, 243.2106.

1-(dec-1-en-1-yl)-3-ethylbenzene 4d (Supplementary Figs. 56–57): ^{1}H NMR (500 MHz, $CDCl_3$): δ 7.23–7.16 (m, 3H), 7.05–7.03 (d, J = 7.04 Hz, 1H), 6.38–6.34 (d, J = 6.36 Hz, 1H), 6.26–6.18 (m, 1H), 2.66–2.60 (m, 2H), 2.23–2.17 (m, 2H), 1.50–1.43 (m, 2H), 1.35–1.22 (m, 13H), 0.90–0.87 (m, 3H); ^{13}C NMR (125 MHz, $CDCl_3$): δ 144.5, 138.1, 131.2, 130.0, 128.6, 126.5, 125.6, 123.4, 33.2, 32.1, 29.7, 29.6, 29.5, 29.4, 29.0, 22.8, 15.8, 14.3; HRMS (m/z): $[M+H]^+$ calcd. for $C_{18}H_{29}$, 245.2264; found, 245.2267.

1-decyl-3-vinylbenzene 4e (Supplementary Figs. 58–59): ^{1}H NMR (500 MHz, $CDCl_3$): δ 7.08–7.07 (m, J = 7.08 Hz, 3H), 6.93–6.91 (m, 1H), 6.58–6.51 (m, 1H), 5.61–6.56 (d, J = 5.58 Hz, 1H), 5.08–5.05 (d, J = 5.07 Hz, 1H), 2.46–2.42 (m, 2H), 1.50–1.42 (m, 2H), 1.16–1.11 (m, 14H), 0.75–0.71 (m, 3H); ^{13}C NMR (125 MHz, $CDCl_3$): δ 143.3, 137.6, 137.2, 128.5, 128.1, 126.5, 123.7, 113.6, 36.1, 32.1, 31.7, 29.8, 29.8, 29.7, 29.5, 29.5, 22.9, 14.3; HRMS (m/z): $[M+H]^+$ calcd. for $C_{18}H_{29}$, 245.2264; found, 245.2261.

1-decyl-3-ethylbenzene 4f (Supplementary Figs. 60–61): ^{1}H NMR (500 MHz, $CDCl_3$): δ 7.25–7.22 (m, 1H), 7.06–7.03 (m, 3H), 2.69–2.60 (m, 4H), 1.68–1.62 (m, 2H), 1.36–1.26 (m, 17H), 0.94–0.91 (m, 3H); ^{13}C NMR (125 MHz, $CDCl_3$): δ 144.3, 143.1, 128.3, 128.2, 125.8, 125.2, 36.2, 32.1, 31.8, 29.8, 29.7, 29.6, 29.5, 29.0, 22.9, 15.8, 14.3; HRMS (m/z): $[M+H]^+$ calcd. for $C_{18}H_{31}$, 247.2420; found, 247.2418.

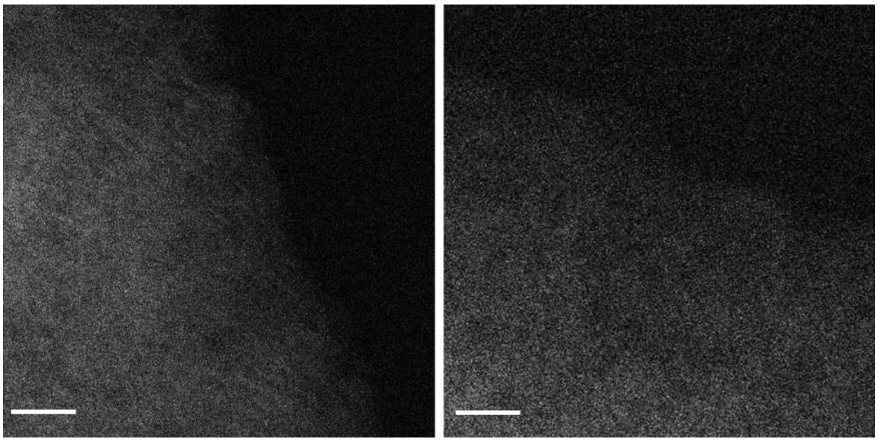

Supplementary Fig. 2 AC HAADF-STEM of N-C. Scale bar, 2 nm.

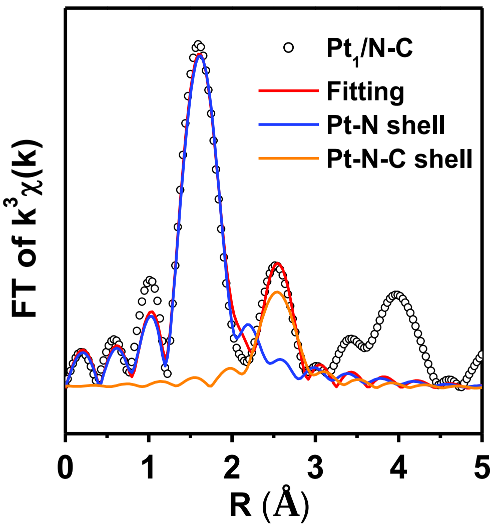

Supplementary Fig. 3 Fourier transform (FT) k^3-weighted $\chi(k)$-function of the EXAFS spectra for Pt L_3-edge and corresponding R-space fitting curves for the Pt_1/N-C catalyst.

Supplementary Table 1. Structural Parameter of EXAFS fitting for the Pt_1/N-C

Sample	Shell	C.N. [a]	R (Å) [b]	σ^2 ($\times 10^{-3}$ Å^2) [c]	E_0 (eV) [d]
Pt_1/N-C	Pt–N	3.4	2.03 ±0.02	3	0.7
	Pt–N–C	2.2	2.98 ±0.04	2.5	5.4

[a] C.N.: coordination number; [b] R: bond distance; [c] σ^2: Debye-waller factors; [d] E_0: the inner potential correction.

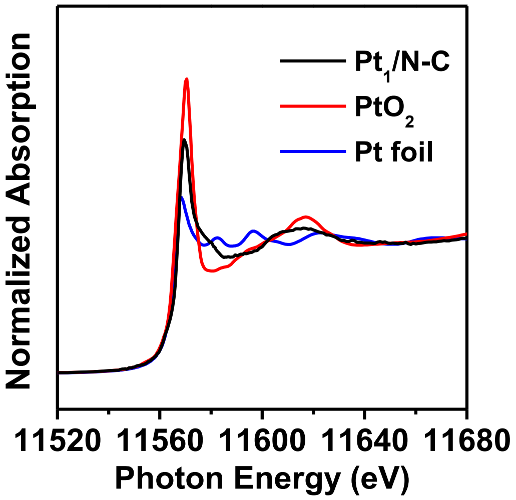

Supplementary Fig. 4 XANES Pt L_3 edge for Pt_1/N-C, PtO_2, and Pt foil.

Supplementary Table 2. Pt content, N content, BET surface area for Pt_1/N-C

Pt (wt%) [a]	N (wt%) [b]	BET surface area ($m^2 g^{-1}$) [c]
0.43	5.17	595

[a] ICP-OES. [b] EA. [c] Sorption isotherm of N_2 at 77 K.

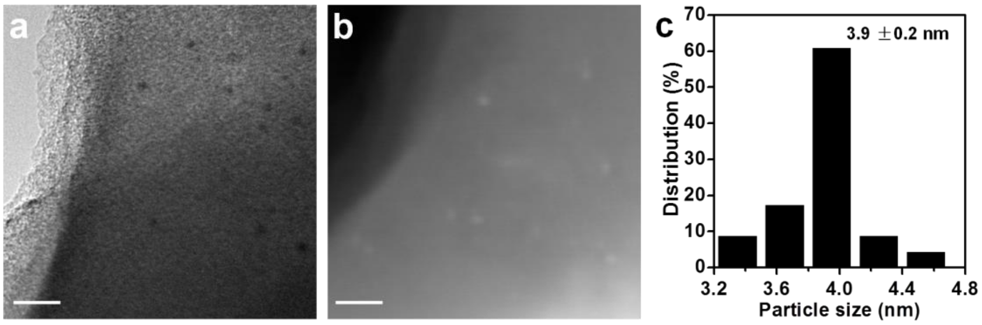

Supplementary Fig. 5 a TEM image, **b** STEM image, and **c** particle size distribution of Pt-NPs/N-C(1:0). Scale bar, 20 nm.

Supplementary Table 3. Pt content, N content, BET surface area for Pt-NPs/N-C

Pt (wt%) [a]	N (wt%) [b]	BET surface area (m^2g^{-1}) [c]
3.31	4.63	700

[a] ICP-OES. [b] EA. [c] Sorption isotherm of N_2 at 77 K.

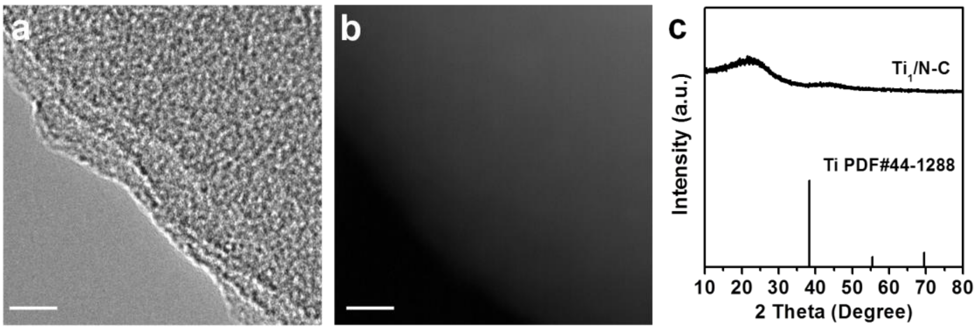

Supplementary Fig. 6 a TEM image, **b** STEM image, and **c** XRD pattern of Ti_1/N-C. Scale bar, 10 nm.

Supplementary Table 4. Ti content, N content, BET surface area for Ti_1/N-C

Ti (wt%) [a]	N (wt%) [b]	BET surface area ($m^2 g^{-1}$) [c]
0.12	4.57	587

[a] ICP-OES. [b] EA. [c] Sorption isotherm of N_2 at 77 K.

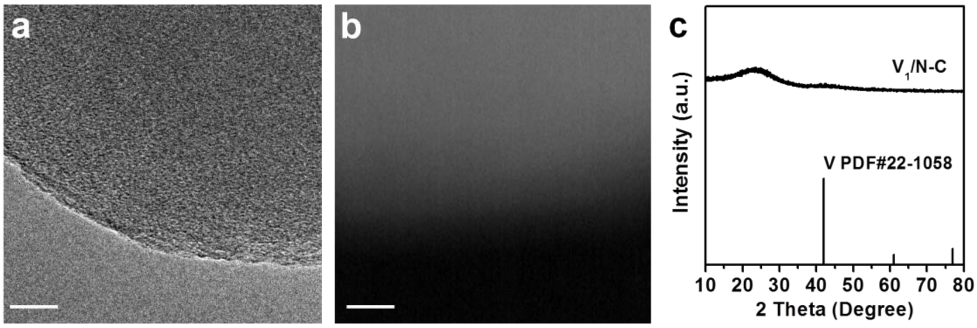

Supplementary Fig. 7 a TEM image, **b** STEM image, and **c** XRD pattern of V_1/N-C. Scale bar, 10 nm.

Supplementary Table 5. V content, N content, BET surface area for V_1/N-C

V (wt%) [a]	N (wt%) [b]	BET surface area ($m^2 g^{-1}$) [c]
0.19	5.05	656

[a] ICP-OES. [b] EA. [c] Sorption isotherm of N_2 at 77 K.

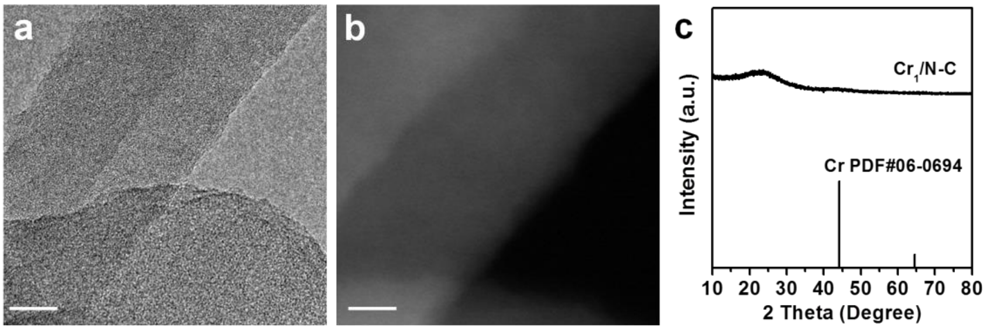

Supplementary Fig. 8 a TEM image, **b** STEM image, and **c** XRD pattern of Cr_1/N-C. Scale bar, 10 nm.

Supplementary Table 6. Cr content, N content, BET surface area for Cr_1/N-C

Cr (wt%) [a]	N (wt%) [b]	BET surface area ($m^2 g^{-1}$) [c]
0.10	5.08	792

[a] ICP-OES. [b] EA. [c] Sorption isotherm of N_2 at 77 K.

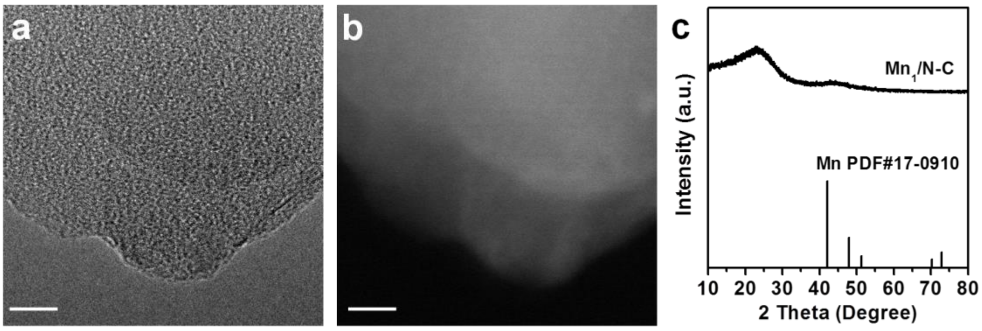

Supplementary Fig. 9 **a** TEM image, **b** STEM image, and **c** XRD pattern of Mn_1/N-C. Scale bar, 10 nm.

Supplementary Table 7. Mn content, N content, BET surface area for Mn_1/N-C

Mn (wt%) [a]	N (wt%) [b]	BET surface area ($m^2 g^{-1}$) [c]
0.07	4.44	790

[a] ICP-OES. [b] EA. [c] Sorption isotherm of N_2 at 77 K.

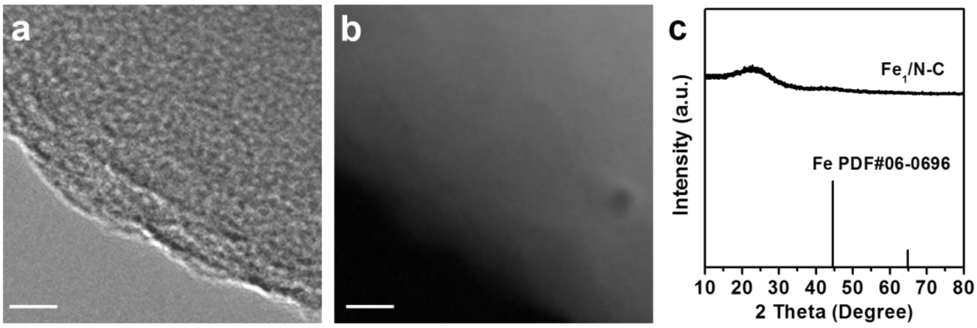

Supplementary Fig. 10 **a** TEM image, **b** STEM image, and **c** XRD pattern of Fe_1/N-C. Scale bar, 10 nm.

Supplementary Table 8. Fe content, N content, BET surface area for Fe_1/N-C

Fe (wt%) [a]	N (wt%) [b]	BET surface area (m^2g^{-1}) [c]
0.18	5.00	784

[a] ICP-OES. [b] EA. [c] Sorption isotherm of N_2 at 77 K.

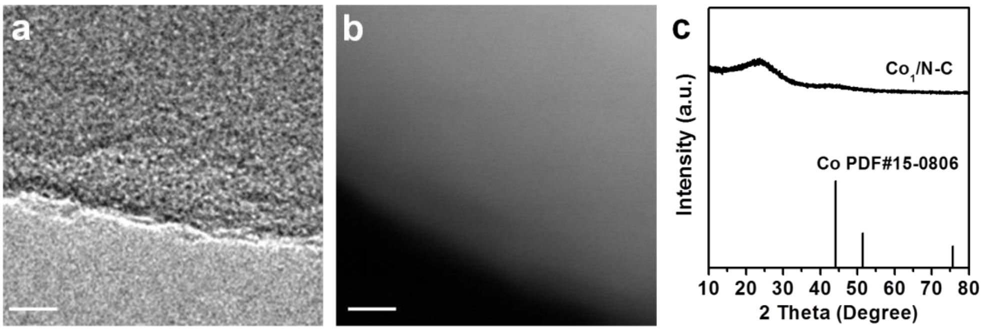

Supplementary Fig. 11 **a** TEM image, **b** STEM image, and **c** XRD pattern of Co_1/N-C. Scale bar, 10 nm.

Supplementary Table 9. Co content, N content, BET surface area for Co_1/N-C

Co (wt%) [a]	N (wt%) [b]	BET surface area (m^2g^{-1}) [c]
0.20	4.86	834

[a] ICP-OES. [b] EA. [c] Sorption isotherm of N_2 at 77 K.

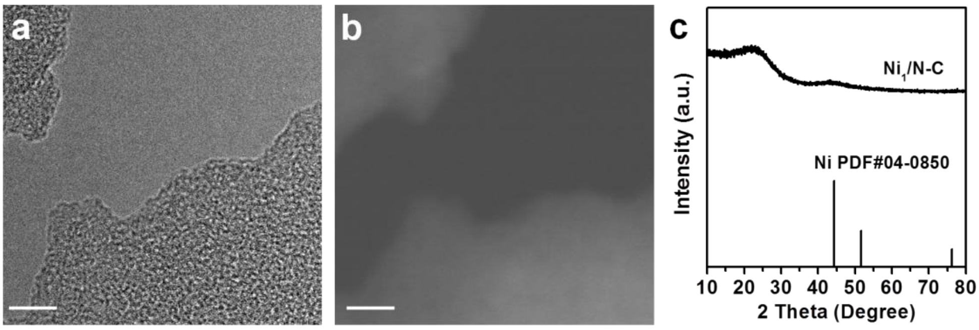

Supplementary Fig. 12 a TEM image, **b** STEM image, and **c** XRD pattern of Ni_1/N-C. Scale bar, 10 nm.

Supplementary Table 10. Ni content, N content, BET surface area for Ni_1/N-C

Ni (wt%) [a]	N (wt%) [b]	BET surface area ($m^2 g^{-1}$) [c]
0.22	5.03	677

[a] ICP-OES. [b] EA. [c] Sorption isotherm of N_2 at 77 K.

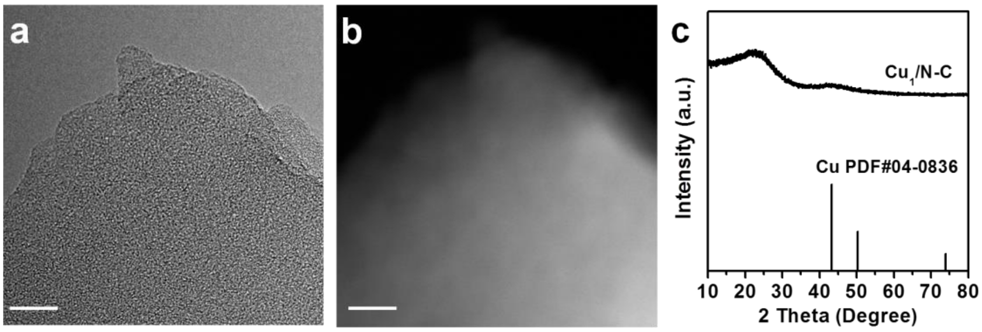

Supplementary Fig. 13 a TEM image, **b** STEM image, and **c** XRD pattern of Cu_1/N-C. Scale bar, 10 nm.

Supplementary Table 11. Cu content, N content, BET surface area for Cu_1/N-C

Cu (wt%) [a]	N (wt%) [b]	BET surface area ($m^2 g^{-1}$) [c]
0.21	5.13	746

[a] ICP-OES. [b] EA. [c] Sorption isotherm of N_2 at 77 K.

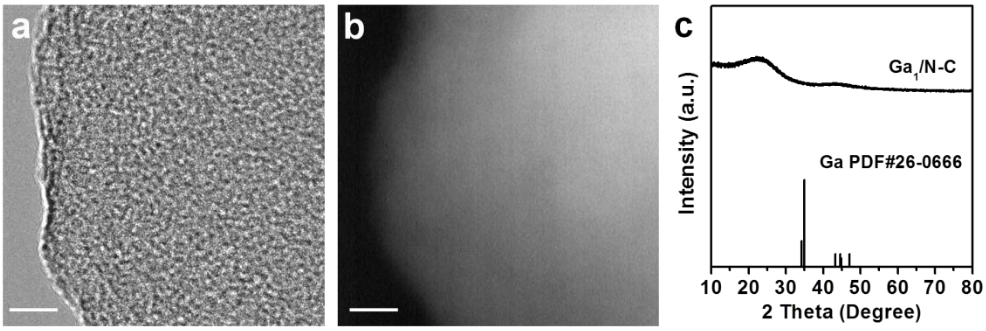

Supplementary Fig. 14 a TEM image, **b** STEM image, and **c** XRD pattern of Ga_1/N-C. Scale bar, 10 nm.

Supplementary Table 12. Ga content, N content, BET surface area for Ga_1/N-C

Ga (wt%) [a]	N (wt%) [b]	BET surface area (m^2g^{-1}) [c]
0.06	4.68	841

[a] ICP-OES. [b] EA. [c] Sorption isotherm of N_2 at 77 K.

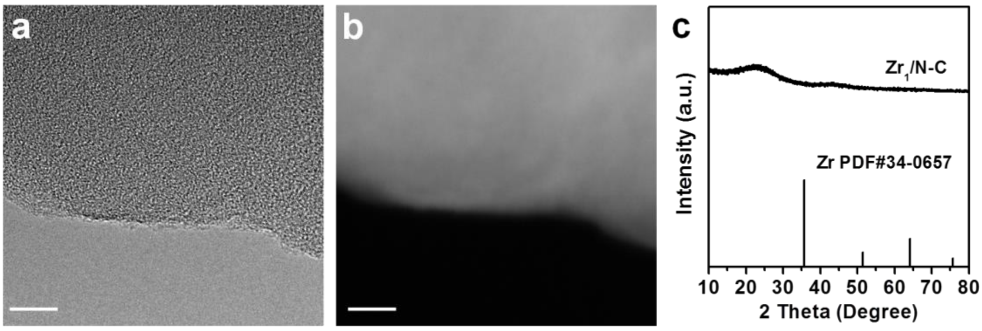

Supplementary Fig. 15 a TEM image, **b** STEM image, and **c** XRD pattern of Zr_1/N-C. Scale bar, 10 nm.

Supplementary Table 13. Zr content, N content, BET surface area for Zr_1/N-C

Zr (wt%) [a]	N (wt%) [b]	BET surface area ($m^2 g^{-1}$) [c]
0.05	4.62	524

[a] ICP-OES. [b] EA. [c] Sorption isotherm of N_2 at 77 K.

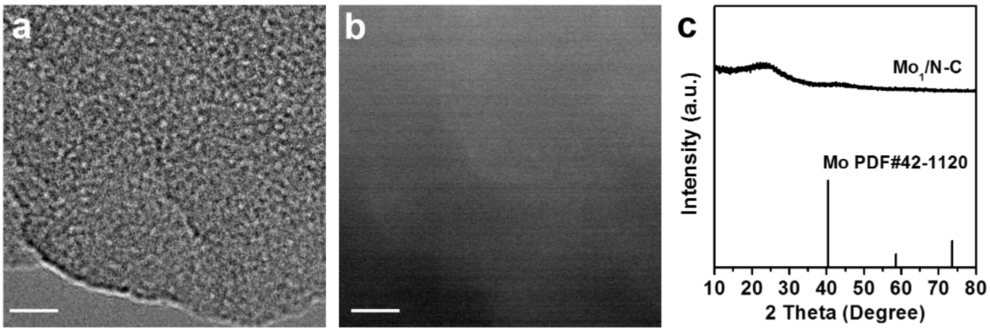

Supplementary Fig. 16 a TEM image, **b** STEM image, and **c** XRD pattern of Mo_1/N-C. Scale bar, 10 nm.

Supplementary Table 14. Mo content, N content, BET surface area for Mo_1/N-C

Mo (wt%) [a]	N (wt%) [b]	BET surface area ($m^2 g^{-1}$) [c]
0.15	4.90	567

[a] ICP-OES. [b] EA. [c] Sorption isotherm of N_2 at 77 K.

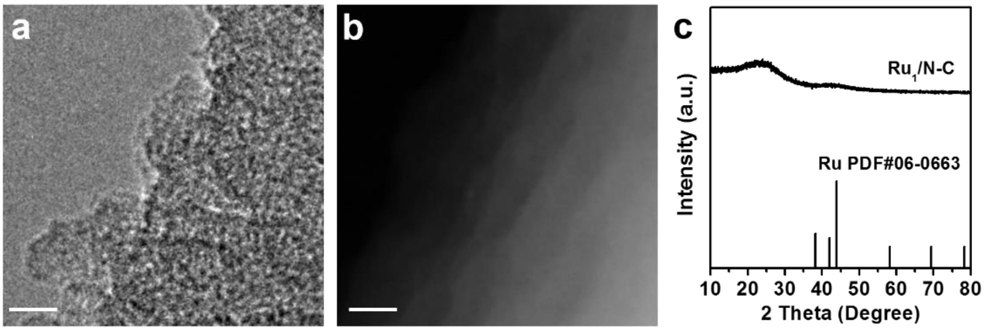

Supplementary Fig. 17 a TEM image, **b** STEM image, and **c** XRD pattern of Ru_1/N-C. Scale bar, 10 nm.

Supplementary Table 15. Ru content, N content, BET surface area for Ru_1/N-C

Ru (wt%) [a]	N (wt%) [b]	BET surface area ($m^2 g^{-1}$) [c]
0.10	5.01	877

[a] ICP-OES. [b] EA. [c] Sorption isotherm of N_2 at 77 K.

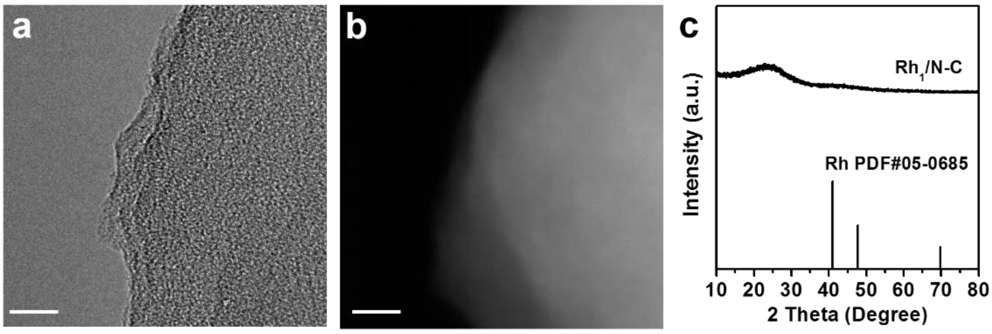

Supplementary Fig. 18 a TEM image, **b** STEM image, and **c** XRD pattern of Rh_1/N-C. Scale bar, 10 nm.

Supplementary Table 16. Rh content, N content, BET surface area for Rh_1/N-C

Rh (wt%) [a]	N (wt%) [b]	BET surface area ($m^2 g^{-1}$) [c]
0.08	4.67	869

[a] ICP-OES. [b] EA. [c] Sorption isotherm of N_2 at 77 K.

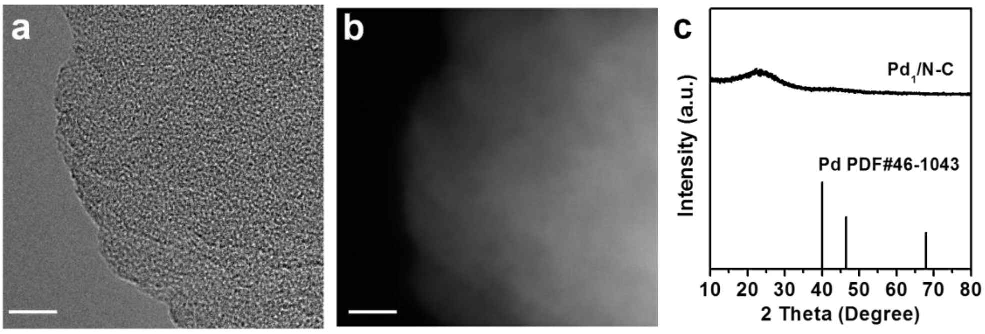

Supplementary Fig. 19 a TEM image, **b** STEM image, and **c** XRD pattern of Pd_1/N-C. Scale bar, 10 nm.

Supplementary Table 17. Pd content, N content, BET surface area for Pd_1/N-C

Pd (wt%) [a]	N (wt%) [b]	BET surface area ($m^2 g^{-1}$) [c]
0.30	4.98	682

[a] ICP-OES. [b] EA. [c] Sorption isotherm of N_2 at 77 K.

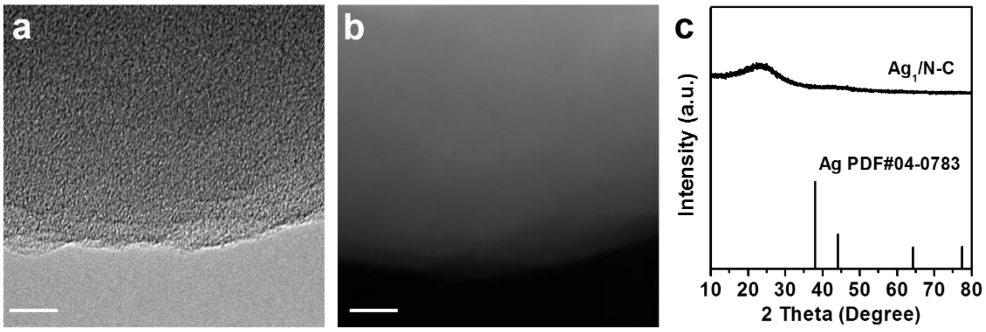

Supplementary Fig. 20 a TEM image, **b** STEM image, and **c** XRD pattern of Ag_1/N-C. Scale bar, 10 nm.

Supplementary Table 18. Ag content, N content, BET surface area for Ag_1/N-C

Ag (wt%) [a]	N (wt%) [b]	BET surface area ($m^2 g^{-1}$) [c]
0.06	4.95	799

[a] ICP-OES. [b] EA. [c] Sorption isotherm of N_2 at 77 K.

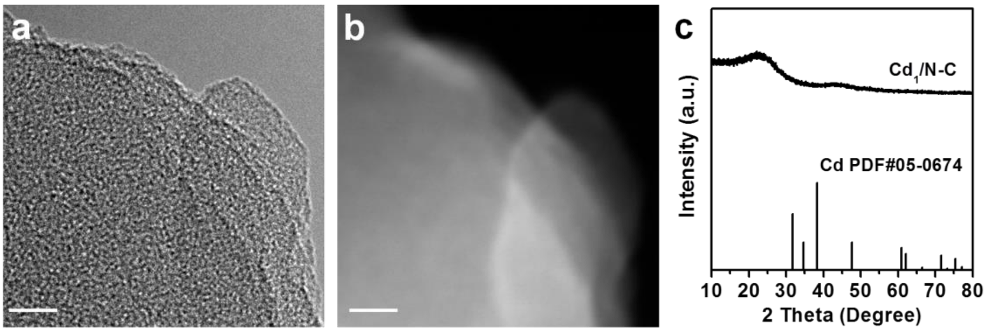

Supplementary Fig. 21 a TEM image, **b** STEM image, and **c** XRD pattern of Cd_1/N-C. Scale bar, 10 nm.

Supplementary Table 19. Cd content, N content, BET surface area for Cd_1/N-C

Cd (wt%) [a]	N (wt%) [b]	BET surface area (m^2g^{-1}) [c]
0.32	4.59	629

[a] ICP-OES. [b] EA. [c] Sorption isotherm of N_2 at 77 K.

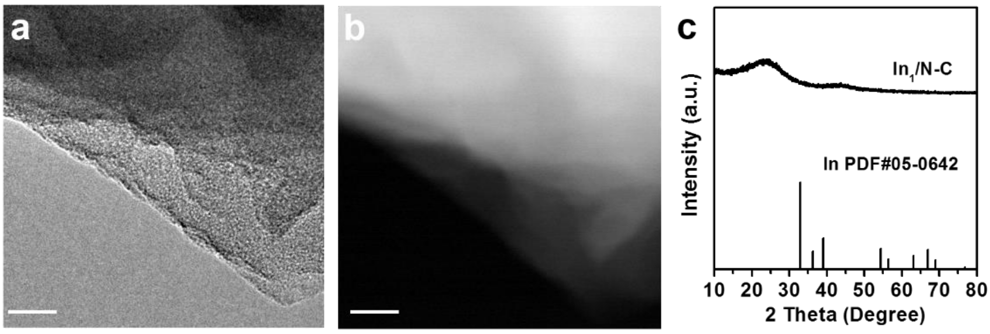

Supplementary Fig. 22 a TEM image, **b** STEM image, and **c** XRD pattern of In_1/N-C. Scale bar, 10 nm.

Supplementary Table 20. In content, N content, BET surface area for In_1/N-C

In (wt%) [a]	N (wt%) [b]	BET surface area ($m^2 g^{-1}$) [c]
0.09	4.57	683

[a] ICP-OES. [b] EA. [c] Sorption isotherm of N_2 at 77 K.

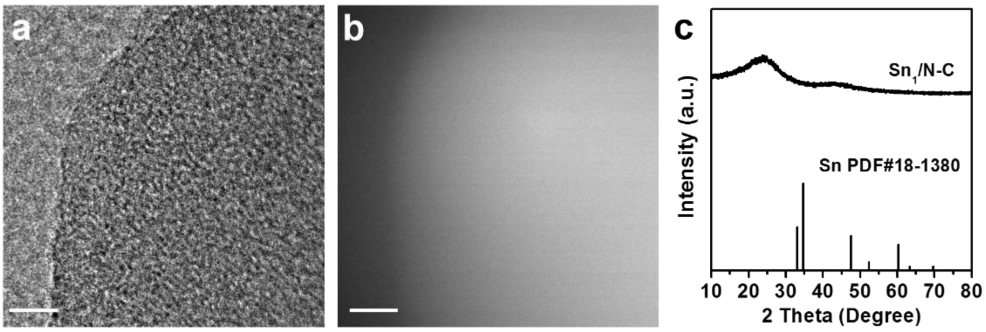

Supplementary Fig. 23 a TEM image, **b** STEM image, and **c** XRD pattern of Sn_1/N-C. Scale bar, 10 nm.

Supplementary Table 21. Sn content, N content, BET surface area for Sn_1/N-C

Sn (wt%) [a]	N (wt%) [b]	BET surface area ($m^2 g^{-1}$) [c]
0.43	4.54	426

[a] ICP-OES. [b] EA. [c] Sorption isotherm of N_2 at 77 K.

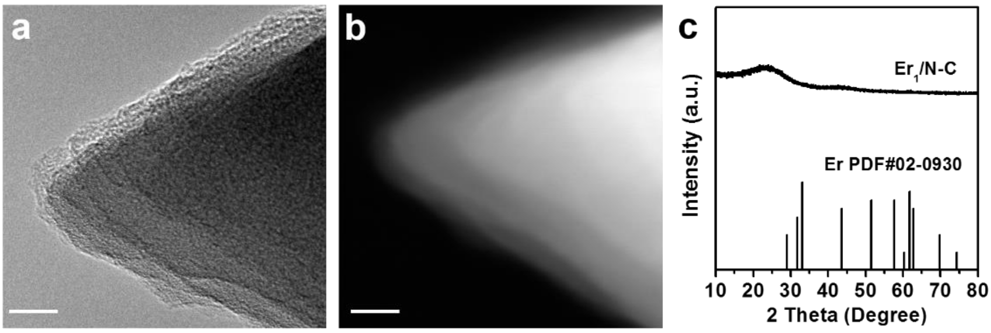

Supplementary Fig. 24 a TEM image, **b** STEM image, and **c** XRD pattern of Er_1/N-C. Scale bar, 10 nm.

Supplementary Table 22. Er content, N content, BET surface area for Er_1/N-C

Er (wt%) [a]	N (wt%) [b]	BET surface area ($m^2 g^{-1}$) [c]
0.06	4.68	793

[a] ICP-OES. [b] EA. [c] Sorption isotherm of N_2 at 77 K.

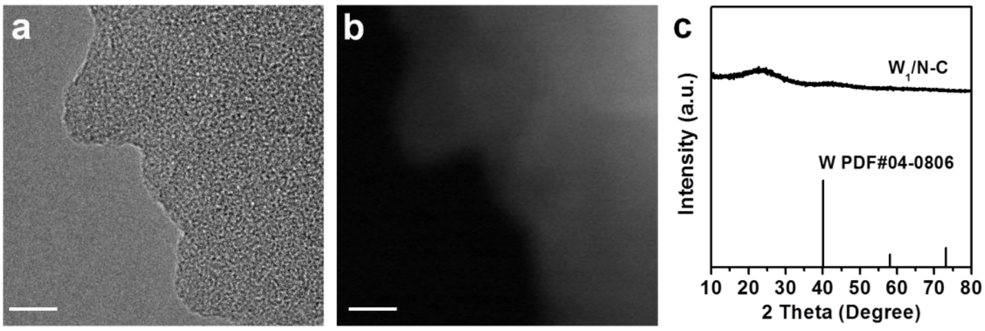

Supplementary Fig. 25 a TEM image, **b** STEM image, and **c** XRD pattern of W_1/N-C. Scale bar, 10 nm.

Supplementary Table 23. W content, N content, BET surface area for W_1/N-C

W (wt%) [a]	N (wt%) [b]	BET surface area ($m^2 g^{-1}$) [c]
0.27	5.34	443

[a] ICP-OES. [b] EA. [c] Sorption isotherm of N_2 at 77 K.

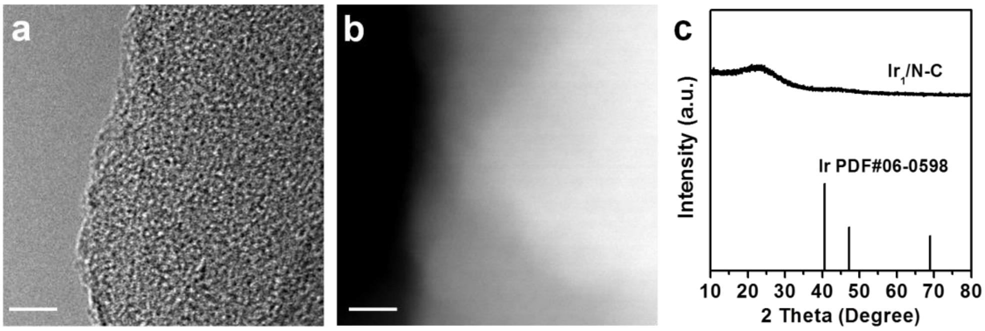

Supplementary Fig. 26 a TEM image, **b** STEM image, and **c** XRD pattern of Ir_1/N-C. Scale bar, 10 nm.

Supplementary Table 24. Ir content, N content, BET surface area for Ir_1/N-C

Ir (wt%) [a]	N (wt%) [b]	BET surface area ($m^2 g^{-1}$) [c]
0.38	5.02	837

[a] ICP-OES. [b] EA. [c] Sorption isotherm of N_2 at 77 K.

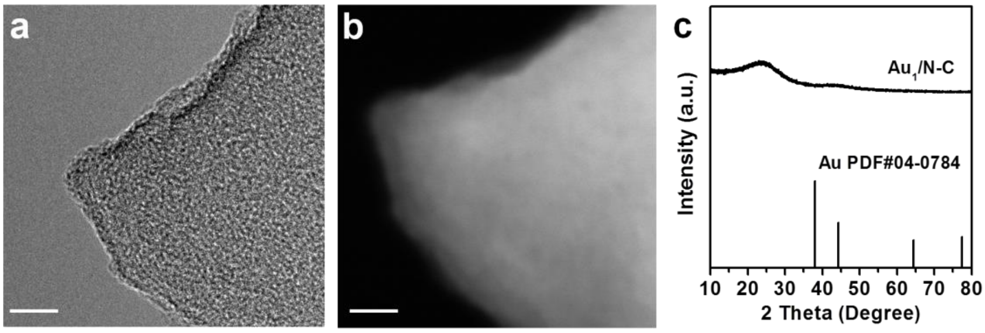

Supplementary Fig. 27 a TEM image, **b** STEM image, and **c** XRD pattern of Au_1/N-C. Scale bar, 10 nm.

Supplementary Table 25. Au content, N content, BET surface area for Au_1/N-C

Au (wt%) [a]	N (wt%) [b]	BET surface area ($m^2 g^{-1}$) [c]
0.19	5.02	783

[a] ICP-OES. [b] EA. [c] Sorption isotherm of N_2 at 77 K.

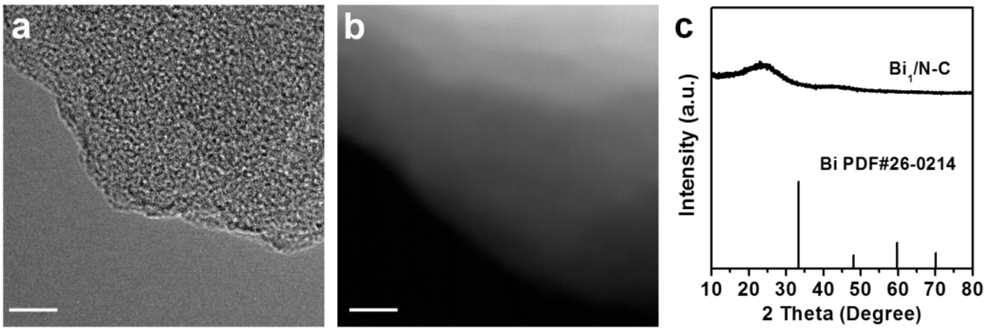

Supplementary Fig. 28 a TEM image, **b** STEM image, and **c** XRD pattern of Bi_1/N-C. Scale bar, 10 nm.

Supplementary Table 26. Bi content, N content, BET surface area for Bi_1/N-C

Bi (wt%) [a]	N (wt%) [b]	BET surface area ($m^2 g^{-1}$) [c]
0.08	5.19	743

[a] ICP-OES. [b] EA. [c] Sorption isotherm of N_2 at 77 K.

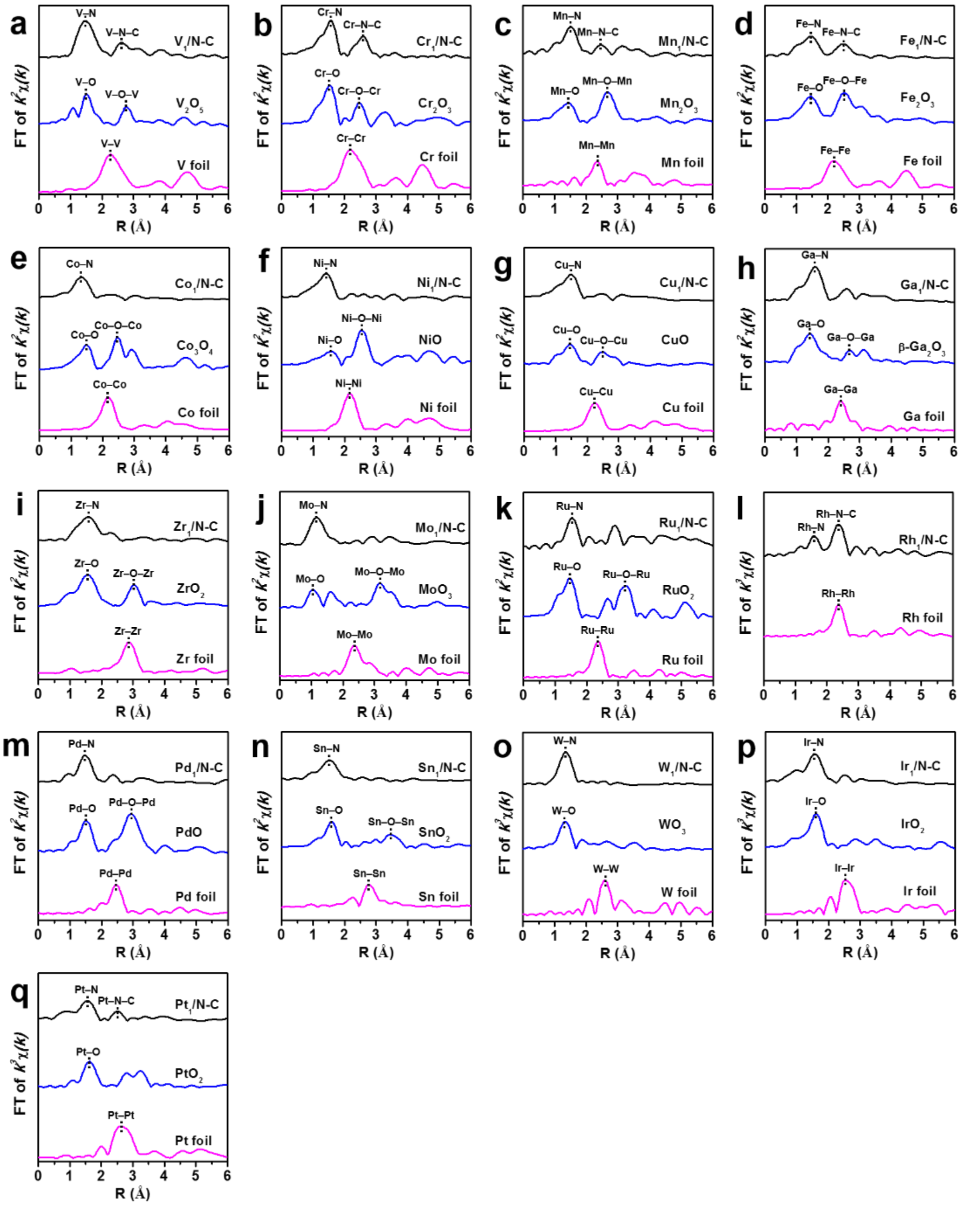

Supplementary Fig. 29 EXAFS data for M_1/N-C, corresponding metal oxides and metal foils (M= V, Cr, Mn, Fe, Co, Ni, Cu, Ga, Zr, Mo, Ru, Rh, Pd, Sn, W, Ir, and Pt, respectively).

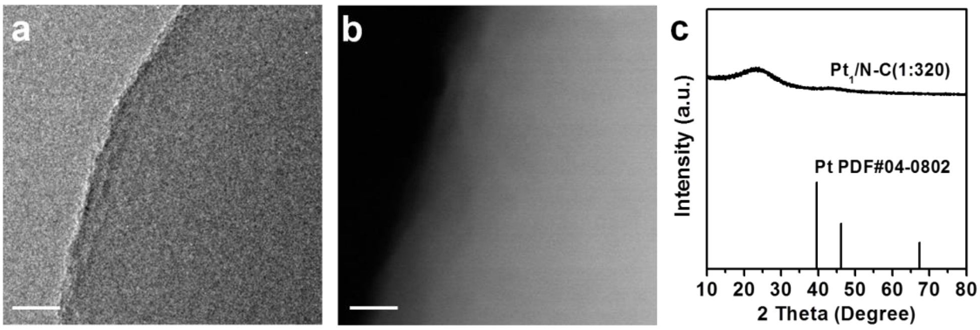

Supplementary Fig. 30 a TEM image, **b** STEM image and **c** XRD pattern of Pt_1/N-C(1:320). Scale bar, 10 nm.

Supplementary Table 27. Pt content, N content, BET surface area for Pt_1/N-C(1:320)

Pt (wt%) [a]	N (wt%) [b]	BET surface area ($m^2 g^{-1}$) [c]
0.06	4.83	644

[a] ICP-OES. [b] EA. [c] Sorption isotherm of N_2 at 77 K.

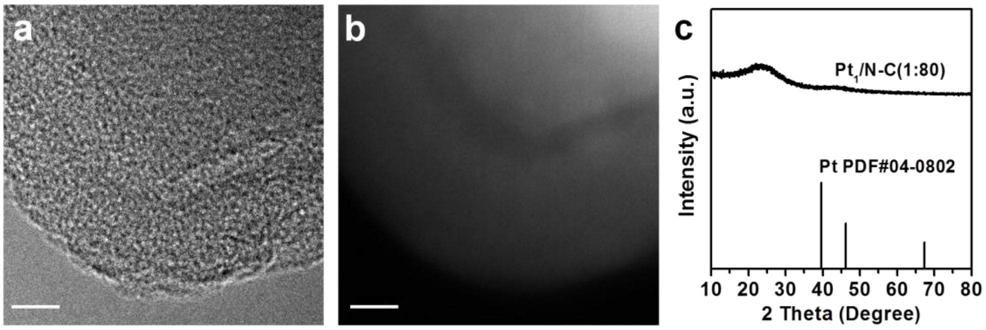

Supplementary Fig. 31 a TEM image, **b** STEM image and **c** XRD pattern of Pt_1/N-C(1:80). Scale bar, 10 nm.

Supplementary Table 28. Pt content, N content, BET surface area for Pt_1/N-C(1:80)

Pt (wt%) [a]	N (wt%) [b]	BET surface area (m^2g^{-1}) [c]
0.21	4.66	632

[a] ICP-OES. [b] EA. [c] Sorption isotherm of N_2 at 77 K.

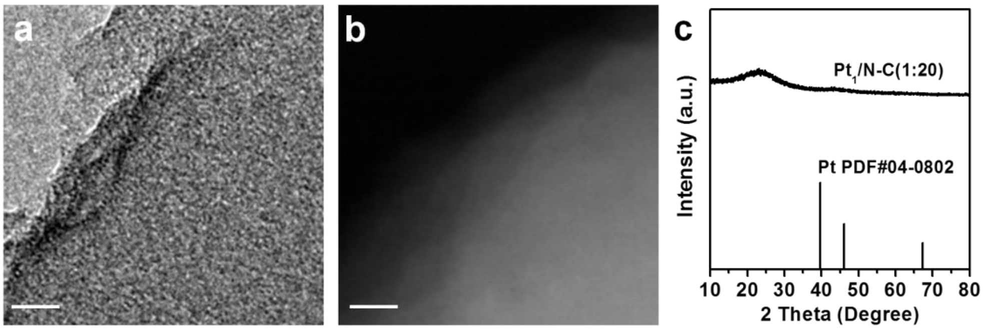

Supplementary Fig. 32 a TEM image, **b** STEM image and **c** XRD pattern of Pt_1/N-C(1:20). Scale bar, 10 nm.

Supplementary Table 29. Pt content, N content, BET surface area for Pt_1/N-C(1:20)

Pt (wt%) [a]	N (wt%) [b]	BET surface area ($m^2 g^{-1}$) [c]
0.73	4.85	656

[a] ICP-OES. [b] EA. [c] Sorption isotherm of N_2 at 77 K.

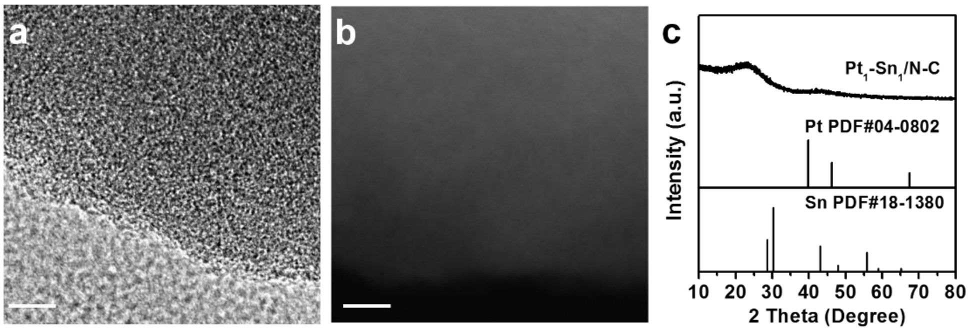

Supplementary Fig. 33 a TEM image, **b** STEM image and **c** XRD pattern of Pt_1-Sn_1/N-C. Scale bar, 10 nm.

Supplementary Table 30. Pt, Sn, and N content, BET surface area for Pt_1-Sn_1/N-C

Pt (wt%) [a]	Sn (wt%) [a]	N (wt%) [b]	BET surface area ($m^2 g^{-1}$) [c]
0.48	0.35	5.19	724

[a] ICP-OES. [b] EA. [c] Sorption isotherm of N_2 at 77 K.

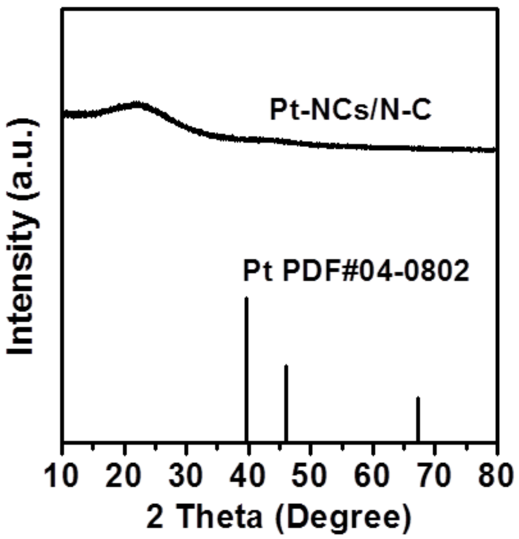

Supplementary Fig. 34 XRD pattern of Pt-NCs/N-C.

Supplementary Table 31. Pt content, N content, BET surface area for Pt-NCs/N-C

Pt (wt%) [a]	N (wt%) [b]	BET surface area (m^2g^{-1}) [c]
0.47	4.82	581

[a] ICP-OES. [b] EA. [c] Sorption isotherm of N_2 at 77 K.

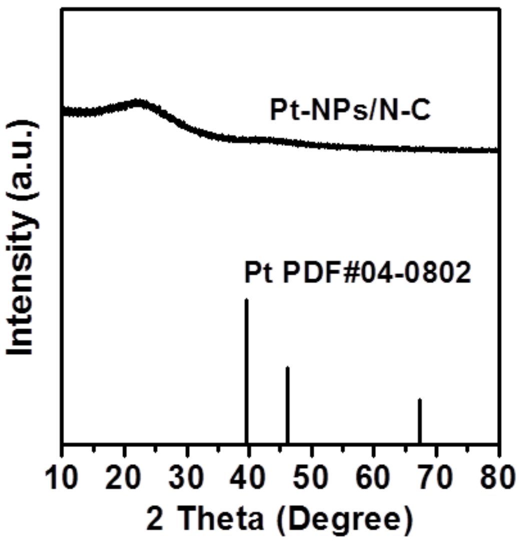

Supplementary Fig. 35 XRD pattern of Pt-NPs/N-C.

Supplementary Table 32. Pt content, N content, BET surface area for Pt-NPs/N-C

Pt (wt%) [a]	N (wt%) [b]	BET surface area (m^2g^{-1}) [c]
0.52	4.51	566

[a] ICP-OES. [b] EA. [c] Sorption isotherm of N_2 at 77 K.

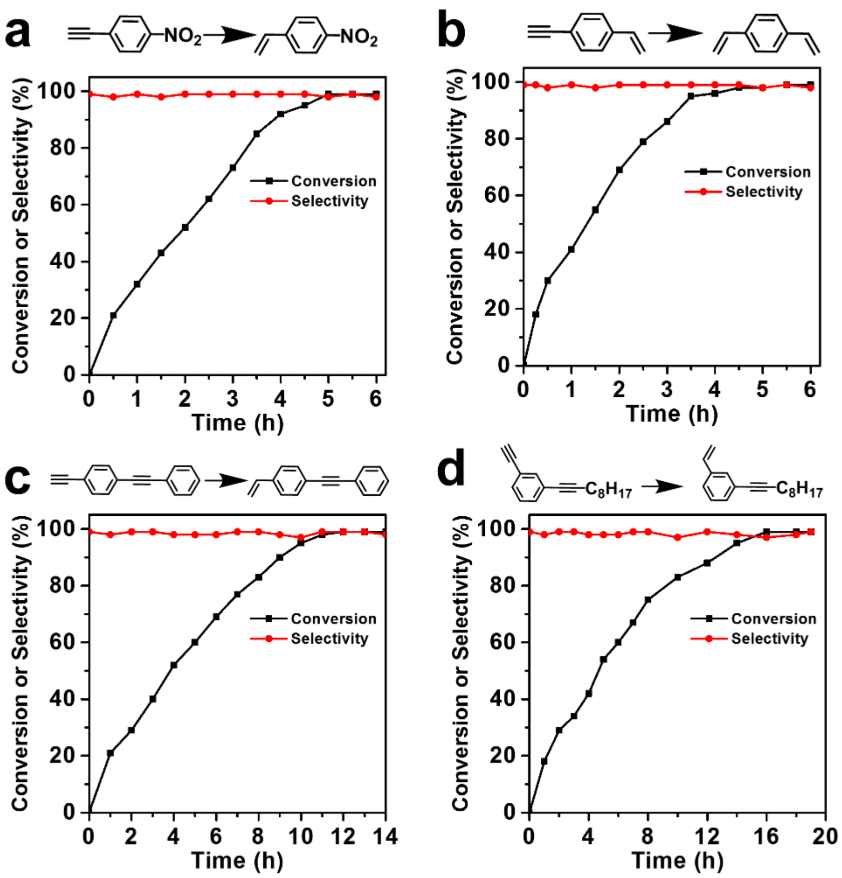

Supplementary Fig. 36 Selective hydrogenation of **a** 1-nitro-4-ethynylbenzene, **b** 1-ethynyl-4-vinylbenzene, **c** 1-ethynyl-4-(phenylethynyl)benzene, and **d** 1-(dec-1-yn-1-yl)-3-ethynylbenzene on Pt_1/N-C. Reaction condition: substrate (0.5 mmol), Pt_1/N-C (Pt:substrate = 1:1200, mol:mol), methanol (2.0 mL), H_2 (1.0 MPa). Reaction temperature: **a** and **b** 50 ℃; **c** and **d** 80 ℃.

Supplementary Table 33. Hydrogenation of substrates with internal alkyne on Pt catalysts with different aggregation states [a]

Entry	Substrate	Product	Pt_1/N-C Atomically dispersed		Pt-NCs/N-C 1.1 nm		Pt-NPs/N-C 6.9 nm	
			TOF (h^{-1})	Sel. (%)	TOF (h^{-1})	Sel. (%)	TOF (h^{-1})	Sel. (%)
1	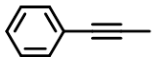	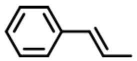	0	—	132	86	2946	83
2	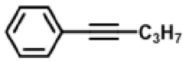	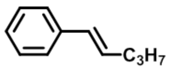	0	—	93	83	2556	81
3 [b]	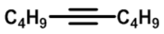	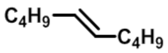	0	—	2860	76	13300	75

[a] Substrates (1.0 mmol), catalyst (Pt:substrates = 1:1200, mol:mol), methanol (2.0 mL), 80 ℃, H_2 (1.0 MPa). b Pt:substrates = 1:4000, mol:mol, 50 ℃. All the conversions were maintained at <20%.

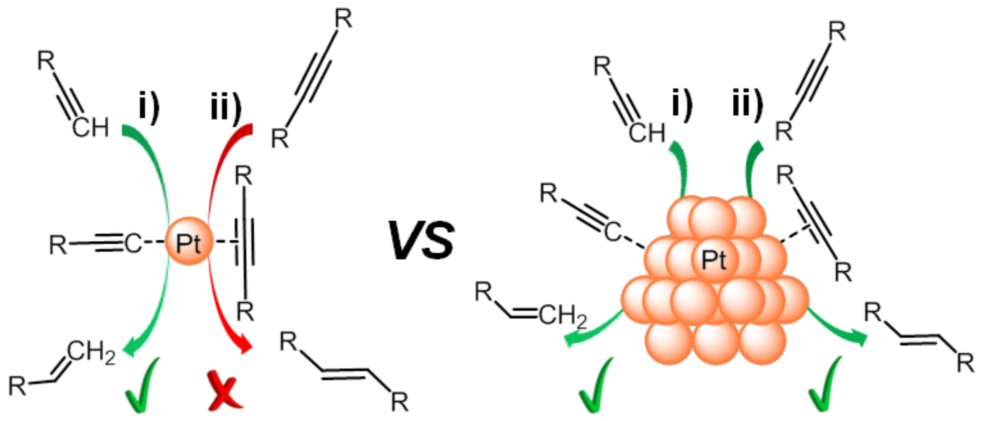

Supplementary Fig. 37 Schematic illustration of the hydrogenation of substrates with terminal alkyne or internal alkyne on Pt SACs and Pt NPs.

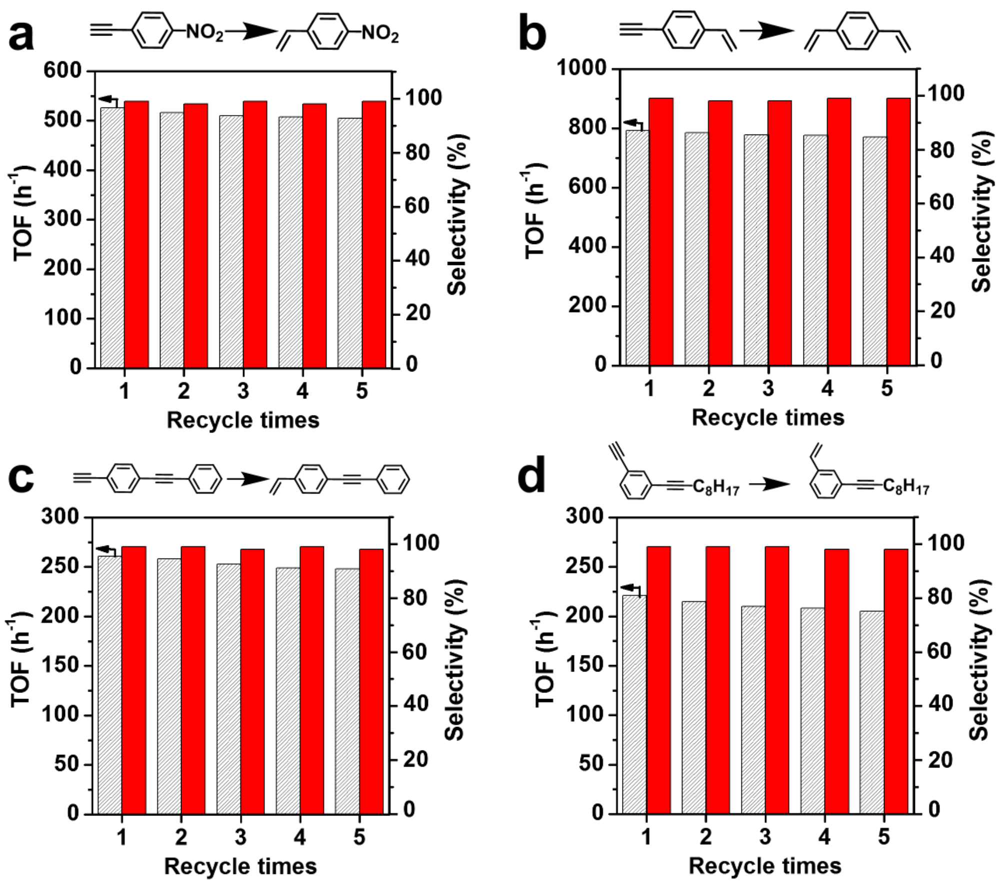

Supplementary Fig. 38 Catalytic performance of Pt_1/N-C in hydrogenation of **a** 1-nitro-4-ethynylbenzene, **b** 1-ethynyl-4-vinylbenzene, **c** 1-ethynyl-4-(phenylethynyl)benzene, and **d** 1-(dec-1-yn-1-yl)-3-ethynylbenzene for 5 catalytic runs. Reaction condition: substrate (0.5 mmol), catalyst (Pt:substrate = 1:1200, mol:mol), methanol (2.0 mL), H_2 (1.0 MPa). Reaction temperature: **a** and **b** 50 ℃; **c** and **d** 80 ℃. All the conversions were maintained at ~20%.

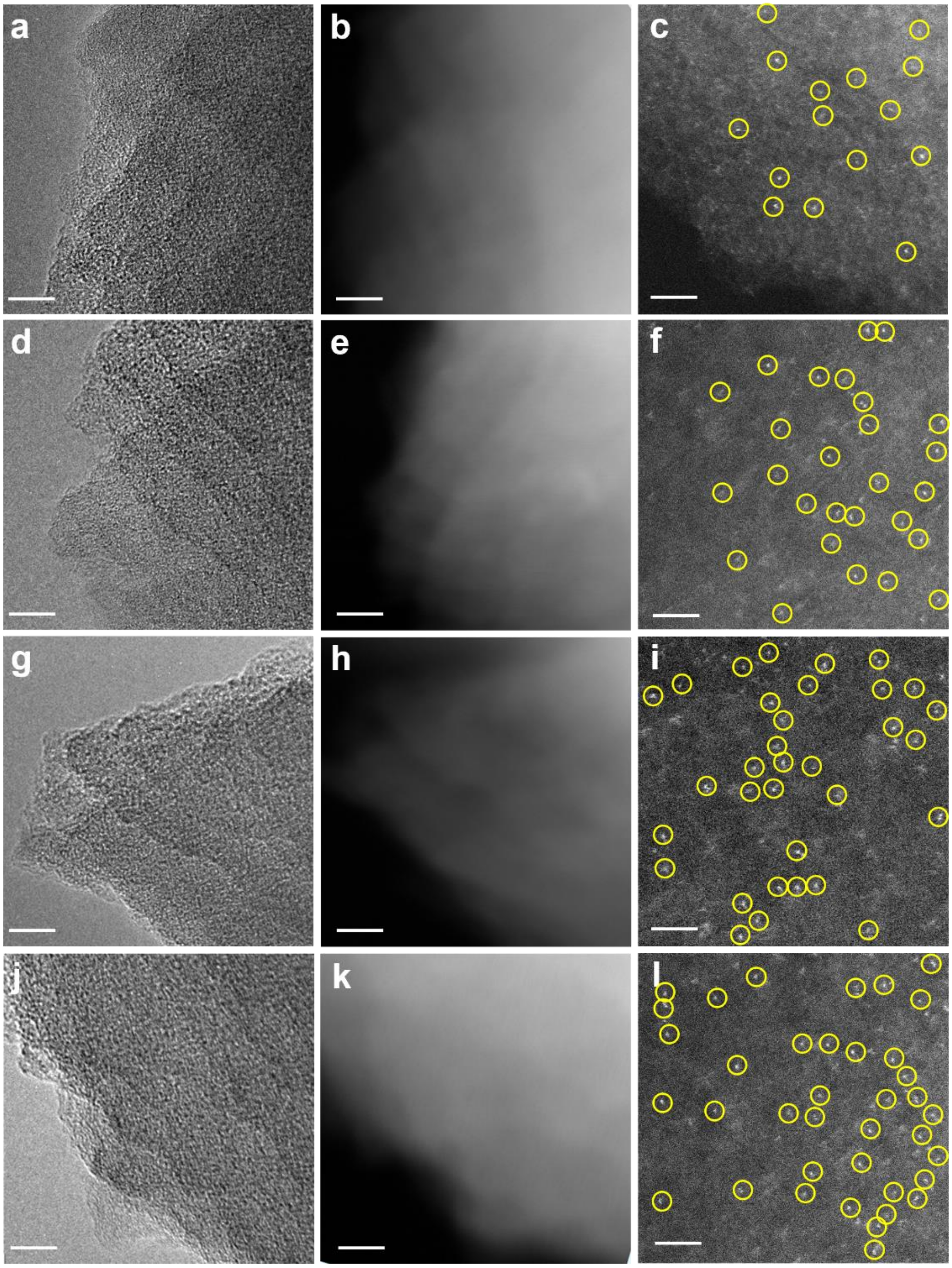

Supplementary Fig. 39 TEM, STEM, and AC HAADF-STEM images of Pt_1/N-C after 5 catalytic runs of hydrogenation of **a–c** 1-nitro-4-ethynylbenzene, **d–f** 1-ethynyl-4-vinylbenzene, **g–i** 1-ethynyl-4-(phenylethynyl)benzene, and **j–l** 1-(dec-1-yn-1-yl)-3-ethynylbenzene, respectively. Scale bar, 10 nm for TEM/STEM images, 2 nm for AC HAADF-STEM images.

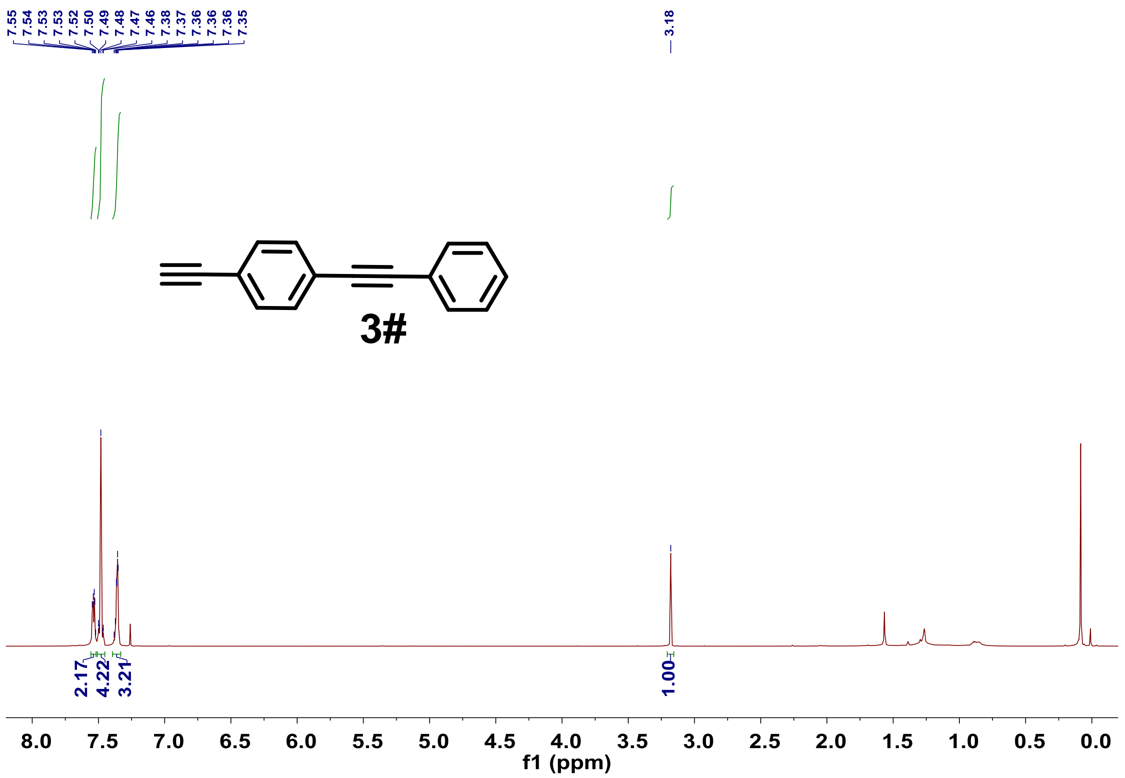

Supplementary Fig. 40 ^{1}H NMR spectrum of 1-ethynyl-4-(phenylethynyl)benzene.

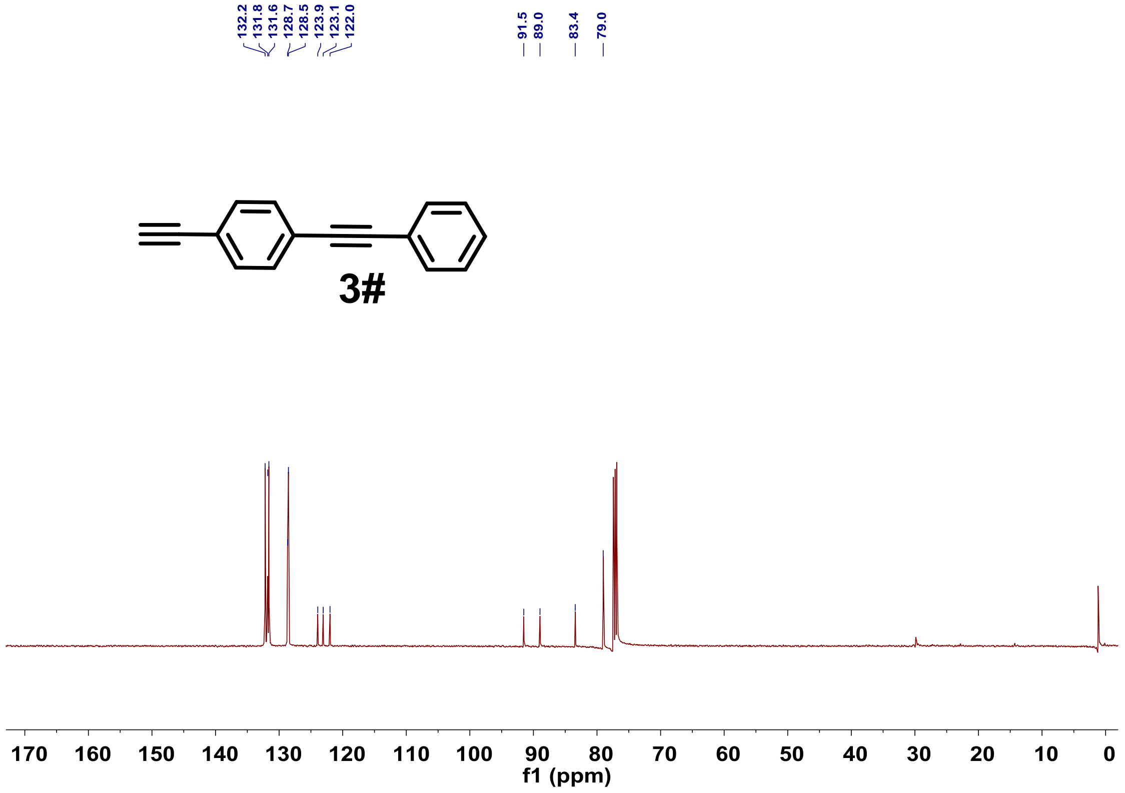

Supplementary Fig. 41 ^{13}C NMR spectrum of 1-ethynyl-4-(phenylethynyl)benzene.

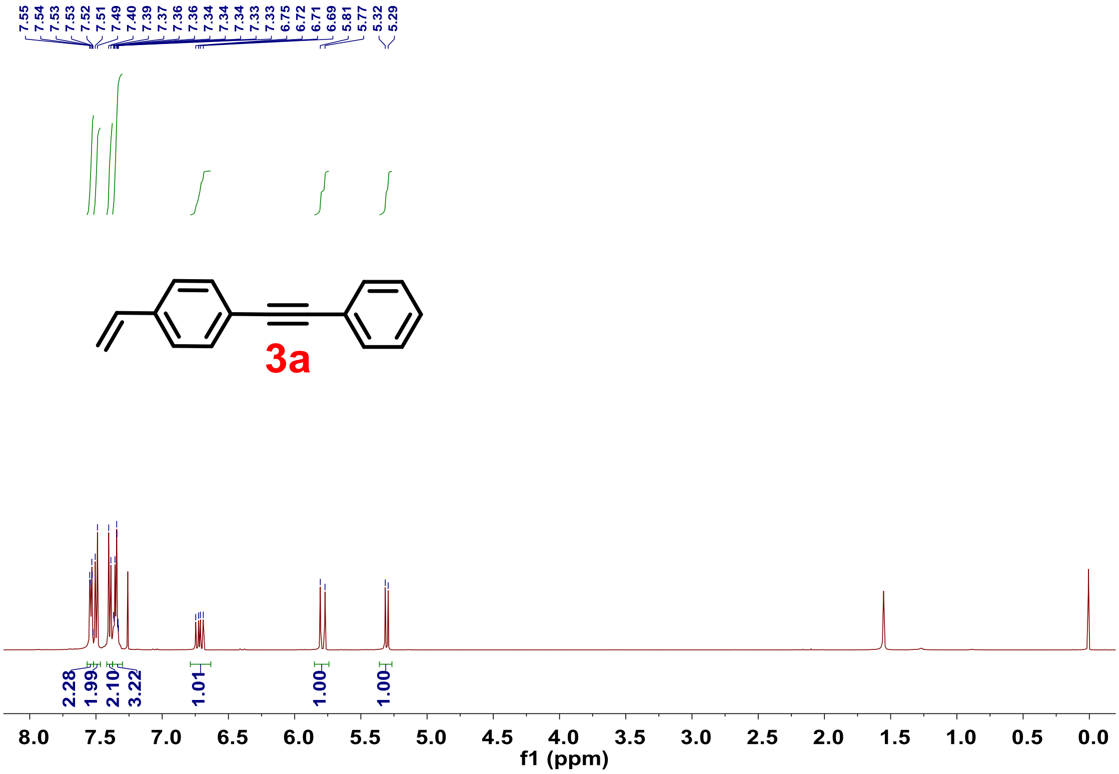

Supplementary Fig. 42 ^{1}H NMR spectrum of 1-(phenylethynyl)-4-vinylbenzene.

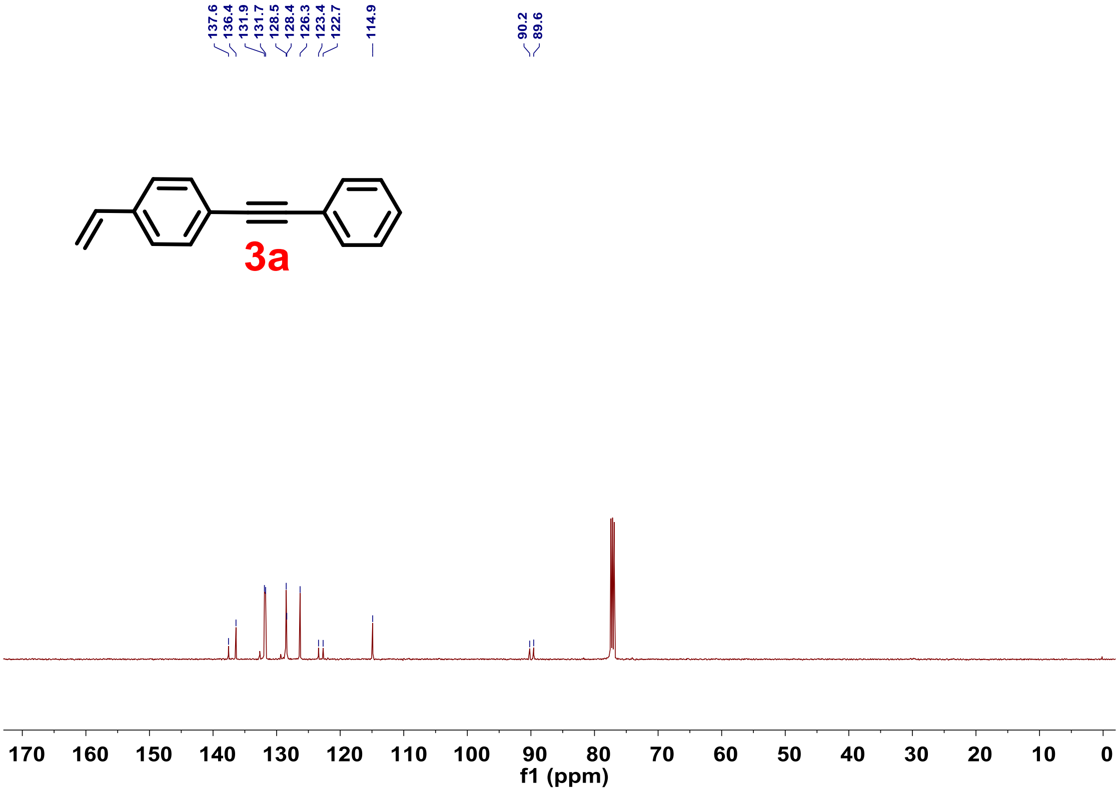

Supplementary Fig. 43 ^{13}C NMR spectrum of 1-(phenylethynyl)-4-vinylbenzene.

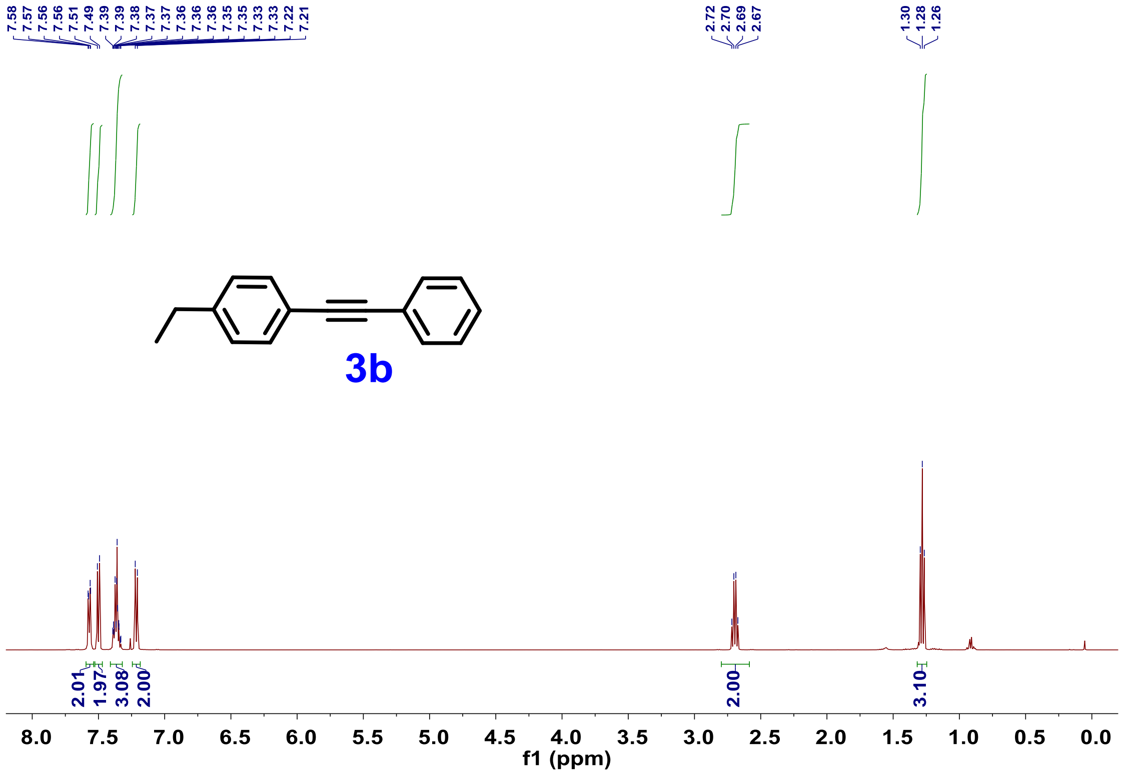

Supplementary Fig. 44 ^{1}H NMR spectrum of 1-ethyl-4-(phenylethynyl)benzene.

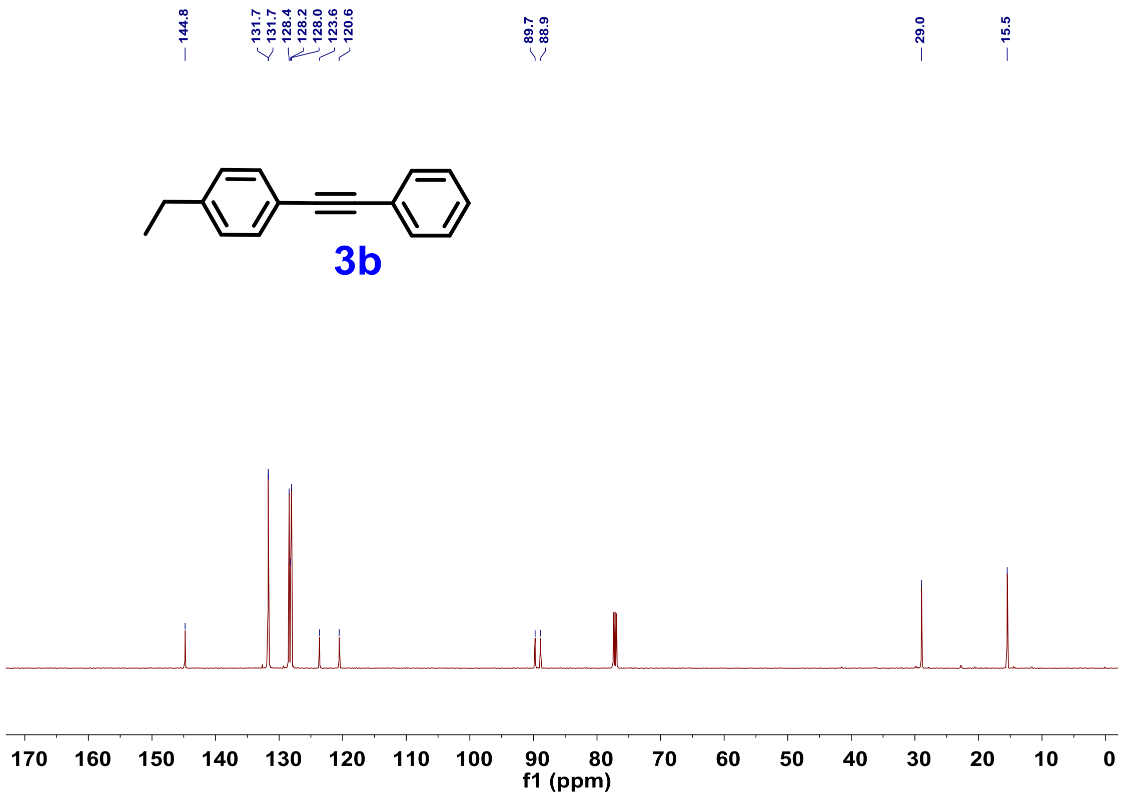

Supplementary Fig. 45 ^{13}C NMR spectrum of 1-ethyl-4-(phenylethynyl)benzene.

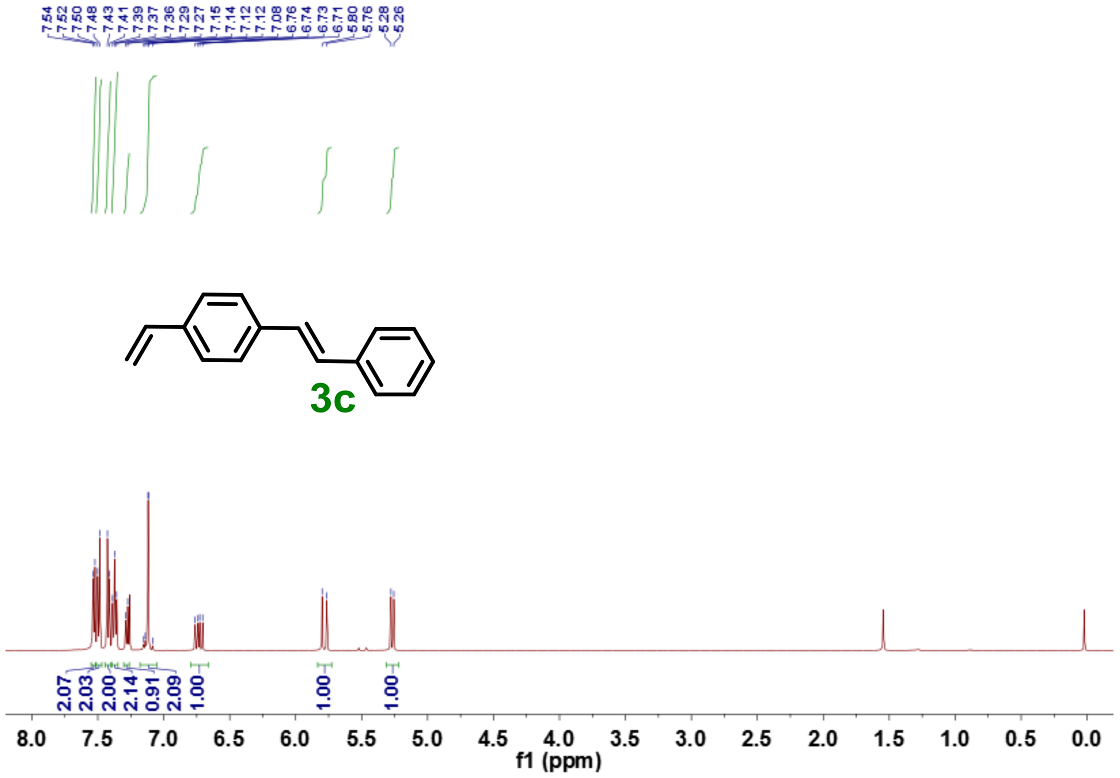

Supplementary Fig. 46 ^{13}C NMR spectrum of 1-styryl-4-vinylbenzene.

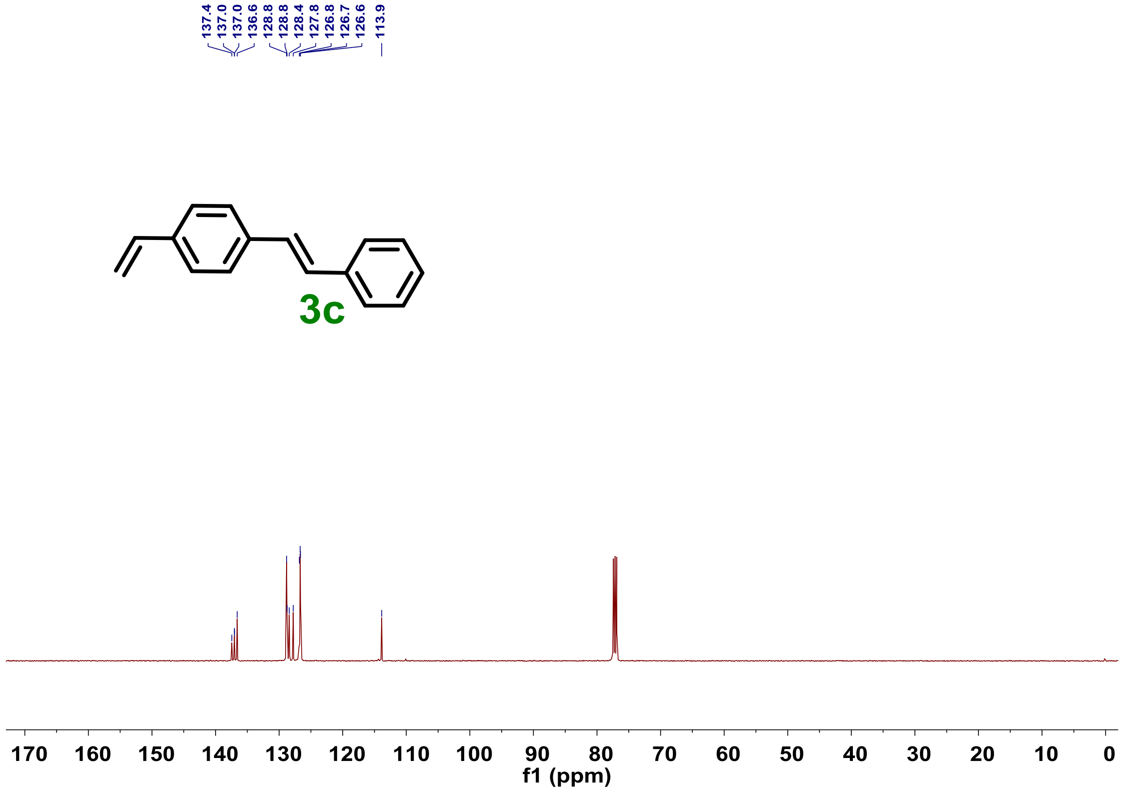

Supplementary Fig. 47 ^{13}C NMR spectrum of 1-styryl-4-vinylbenzene.

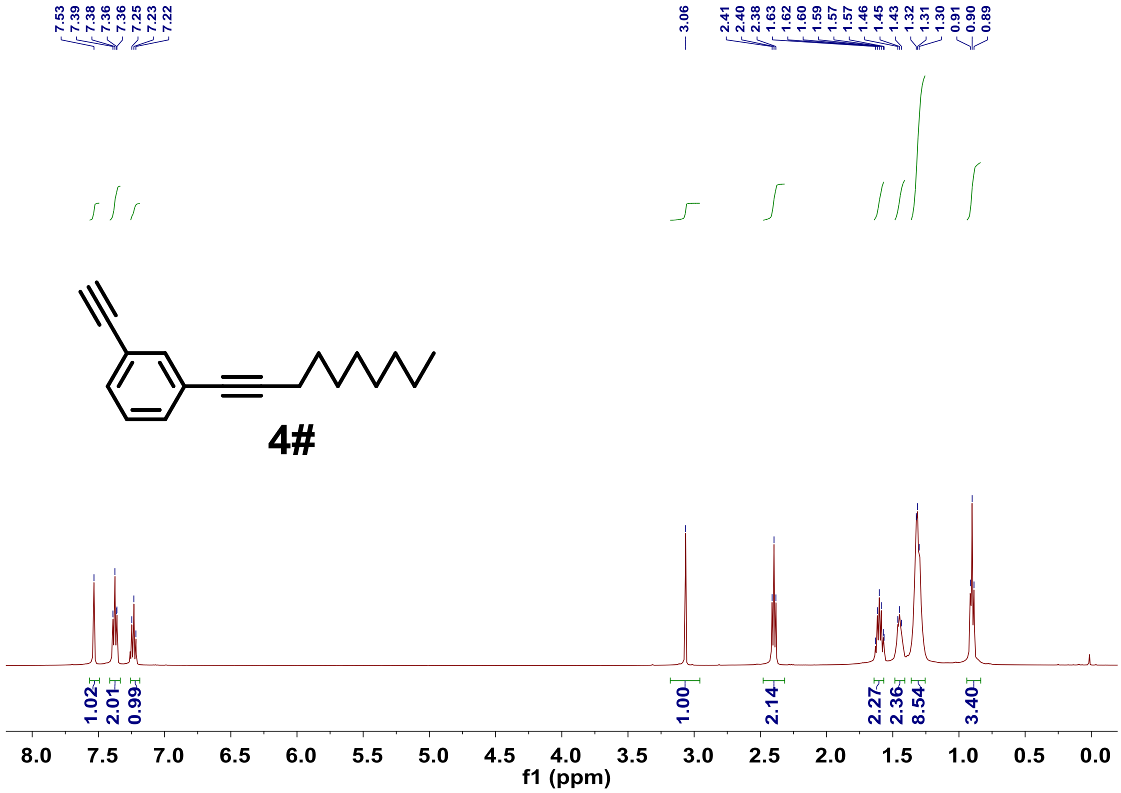

Supplementary Fig. 48 ^{1}H NMR spectrum of 1-(dec-1-yn-1-yl)-3-ethynylbenzene.

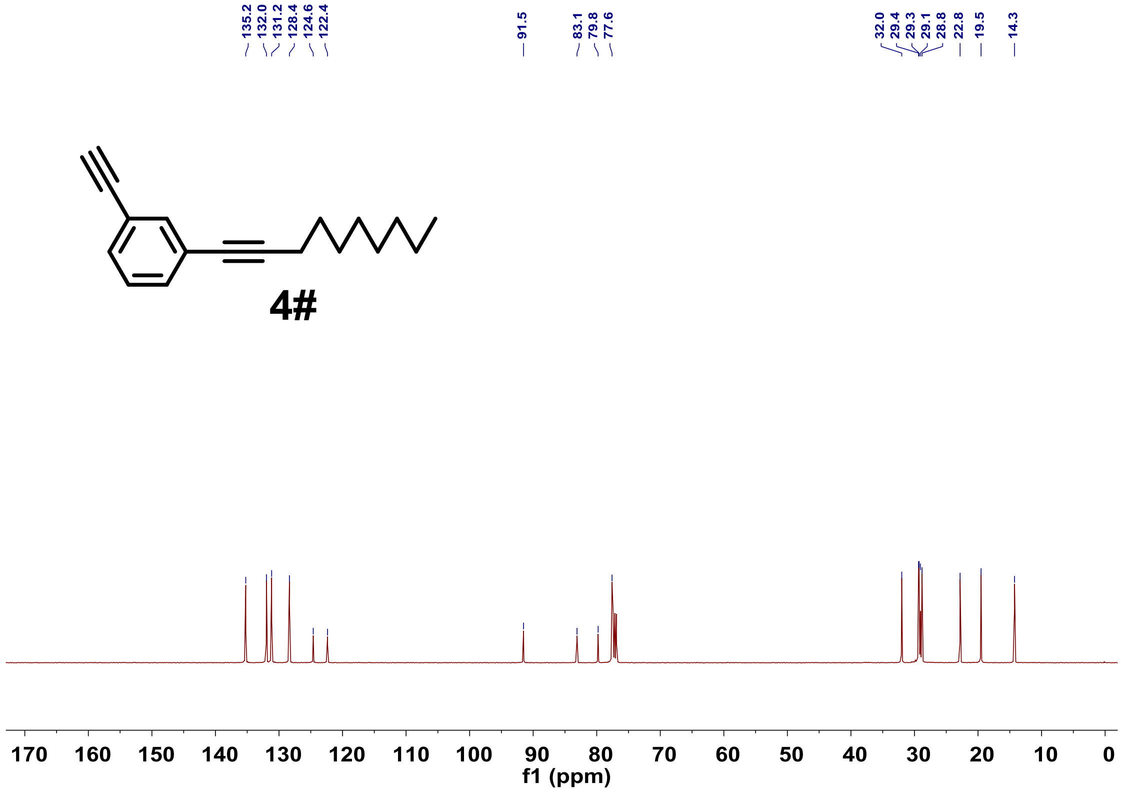

Supplementary Fig. 49 ^{13}C NMR spectrum of 1-(dec-1-yn-1-yl)-3-ethynylbenzene.

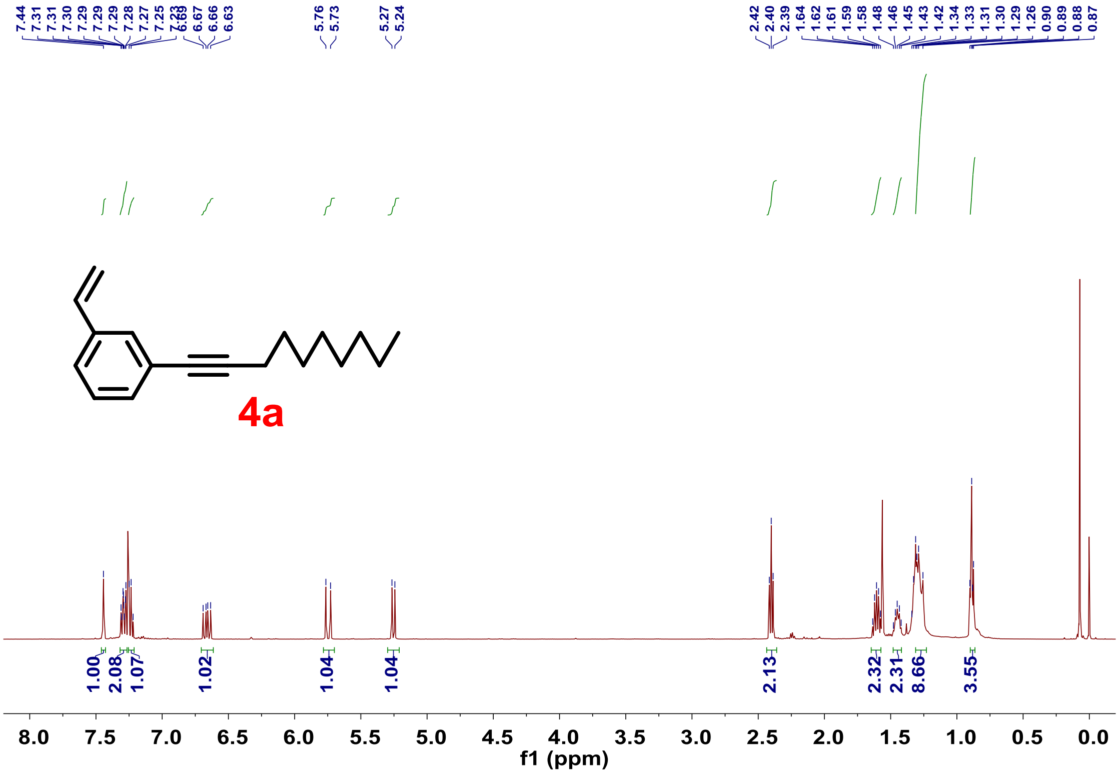

Supplementary Fig. 50 ^{1}H NMR spectrum of 1-(dec-1-yn-1-yl)-3-vinylbenzene.

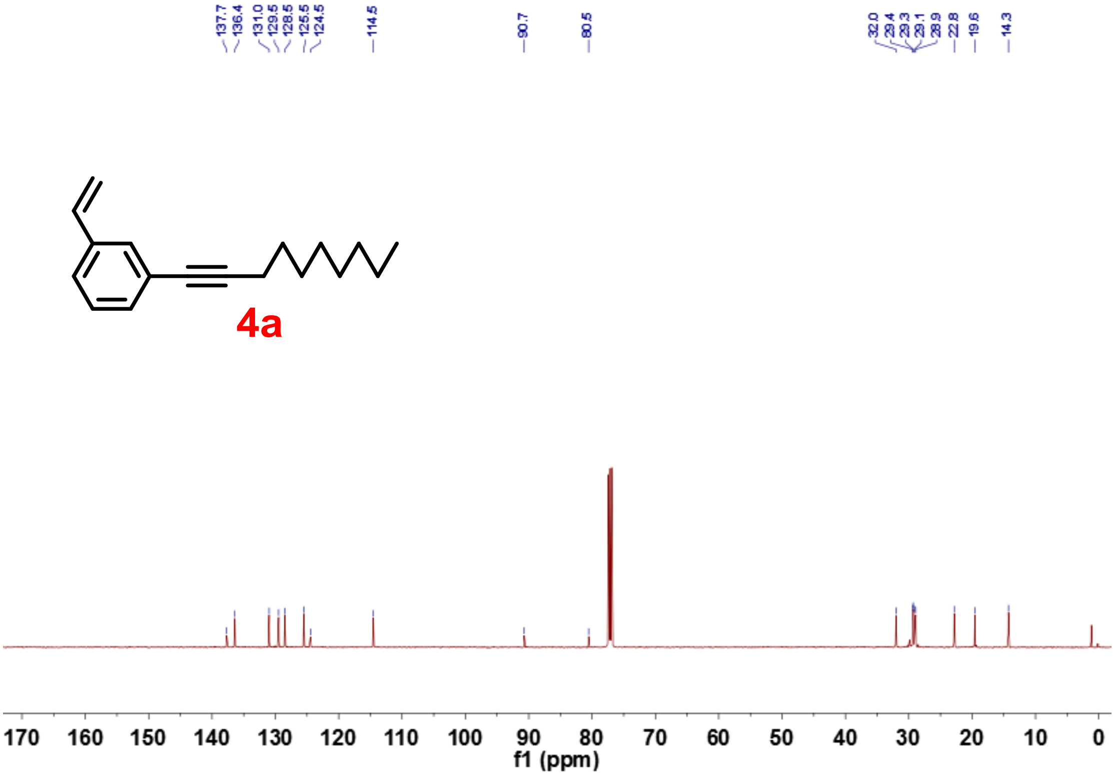

Supplementary Fig. 51 ^{13}C NMR spectrum of1-(dec-1-yn-1-yl)-3-vinylbenzene.

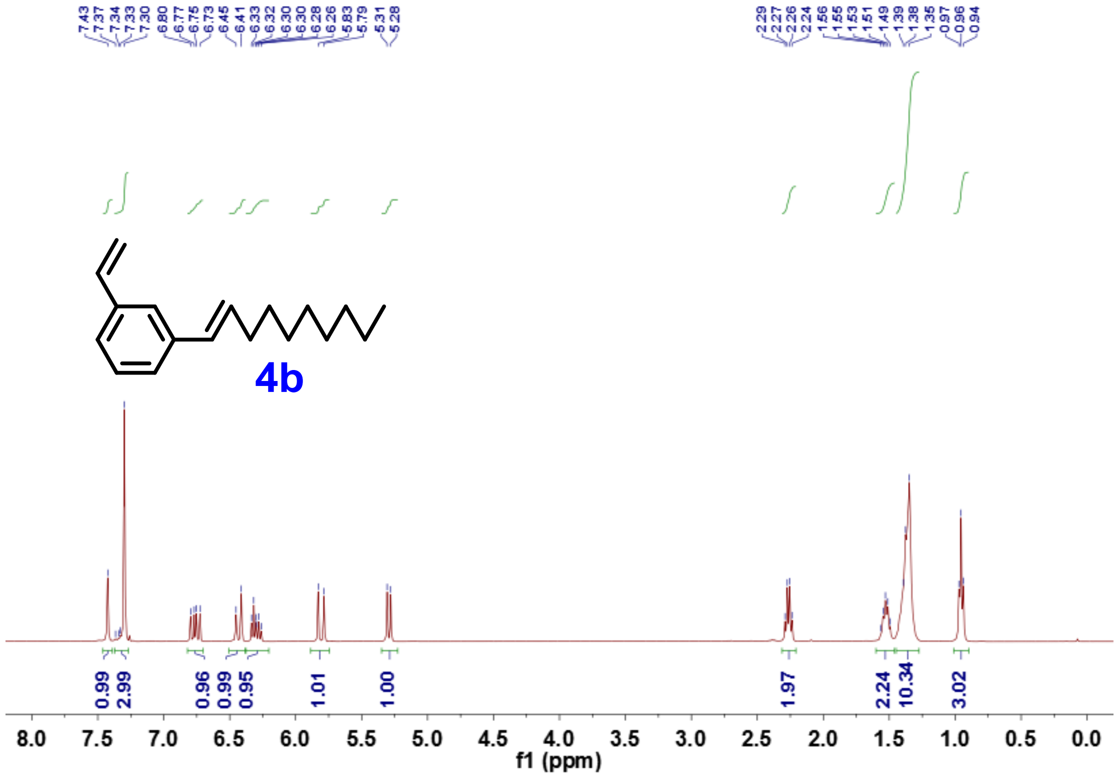

Supplementary Fig. 52 ^{1}H NMR spectrum of 1-(dec-1-en-1-yl)-3-vinylbenzene.

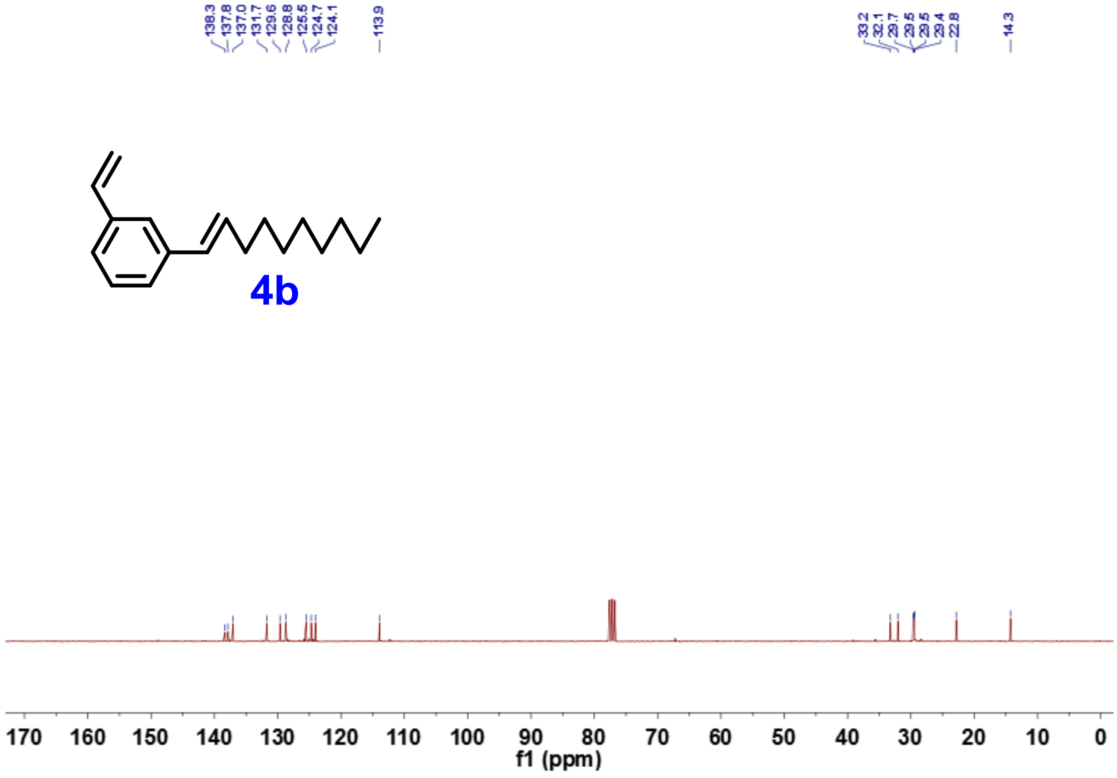

Supplementary Fig. 53 ^{13}C NMR spectrum of 1-(dec-1-en-1-yl)-3-vinylbenzene.

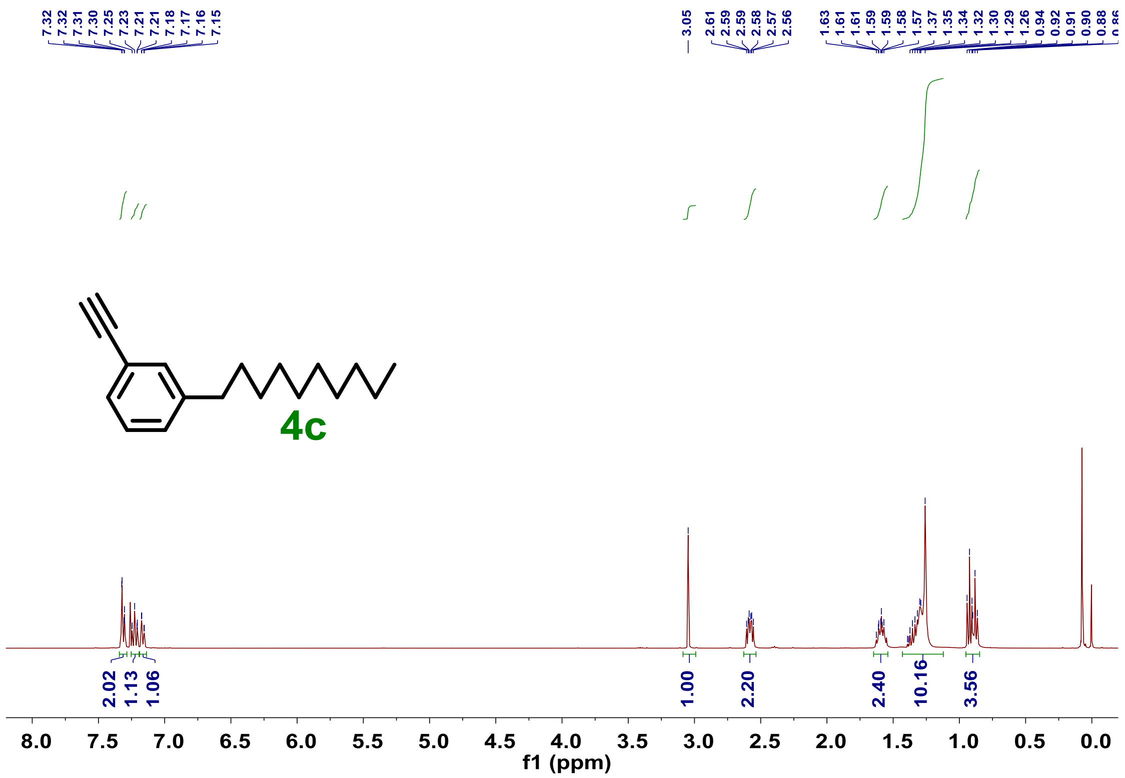

Supplementary Fig. 54 ^{1}H NMR spectrum of 1-decyl-3-ethynylbenzene.

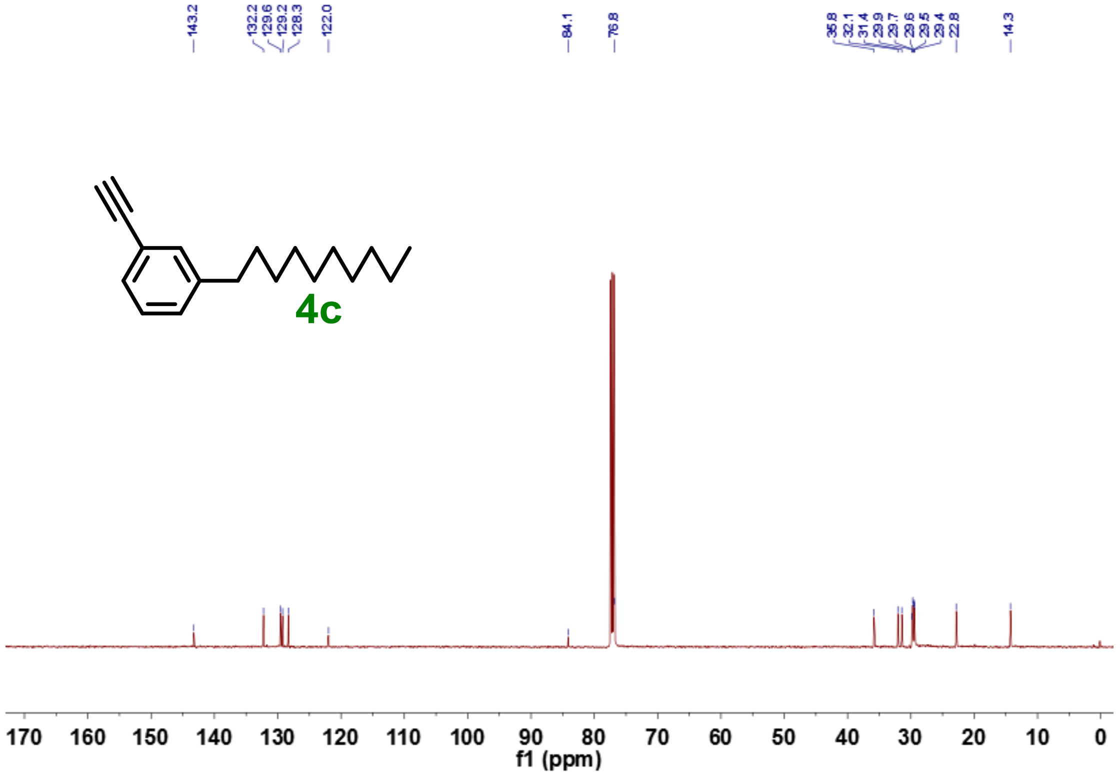

Supplementary Fig. 55 ^{13}C NMR spectrum of 1-decyl-3-ethynylbenzene.

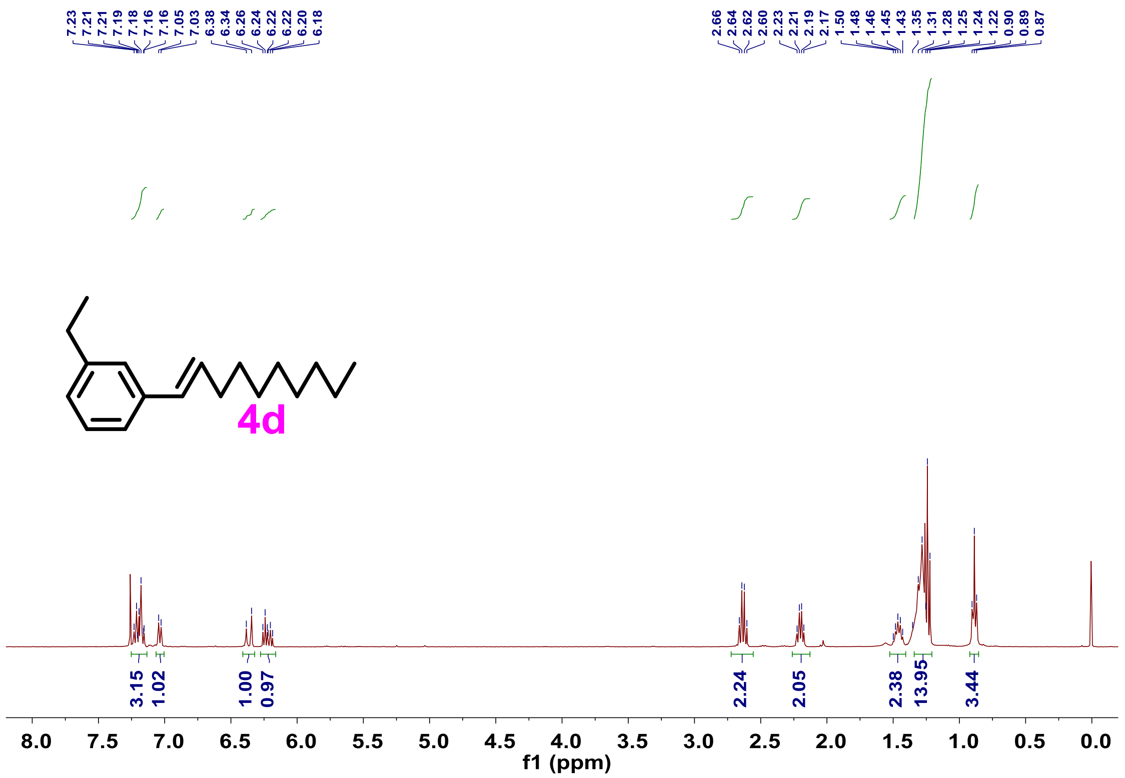

Supplementary Fig. 56 ^{1}H NMR spectrum of 1-(dec-1-en-1-yl)-3-ethylbenzene.

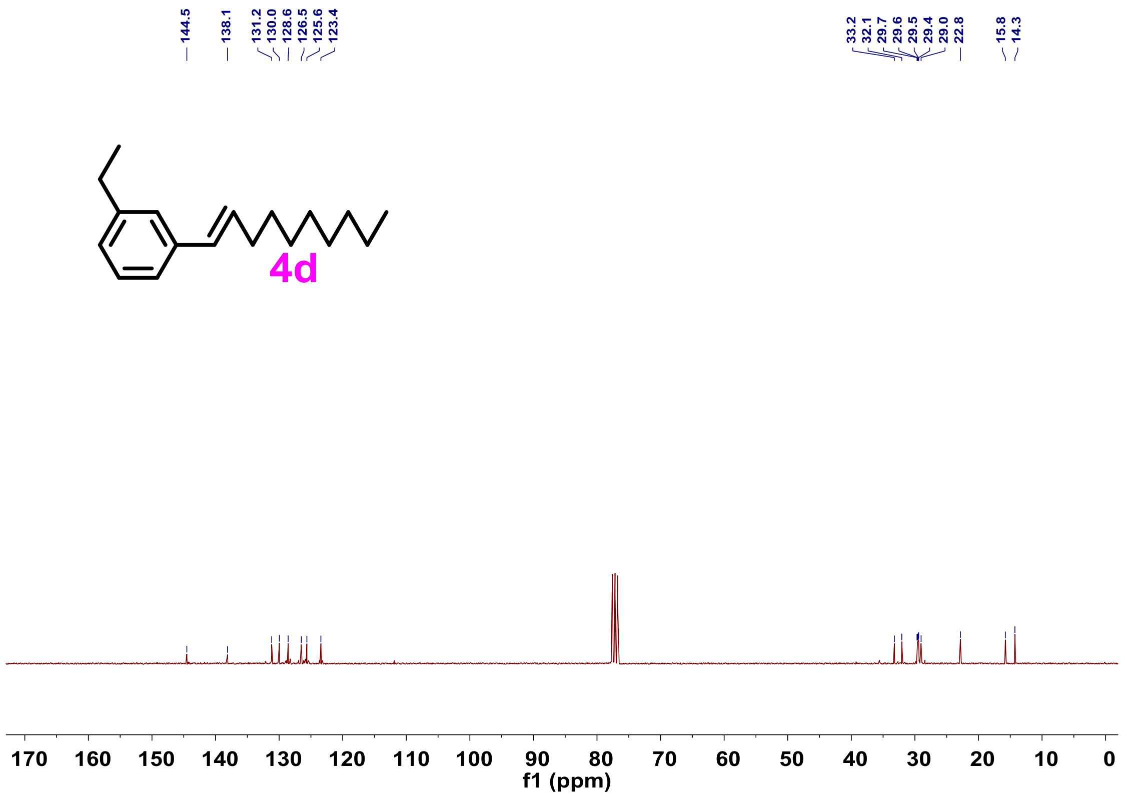

Supplementary Fig. 57 ^{13}C NMR spectrum of 1-(dec-1-en-1-yl)-3-ethylbenzene.

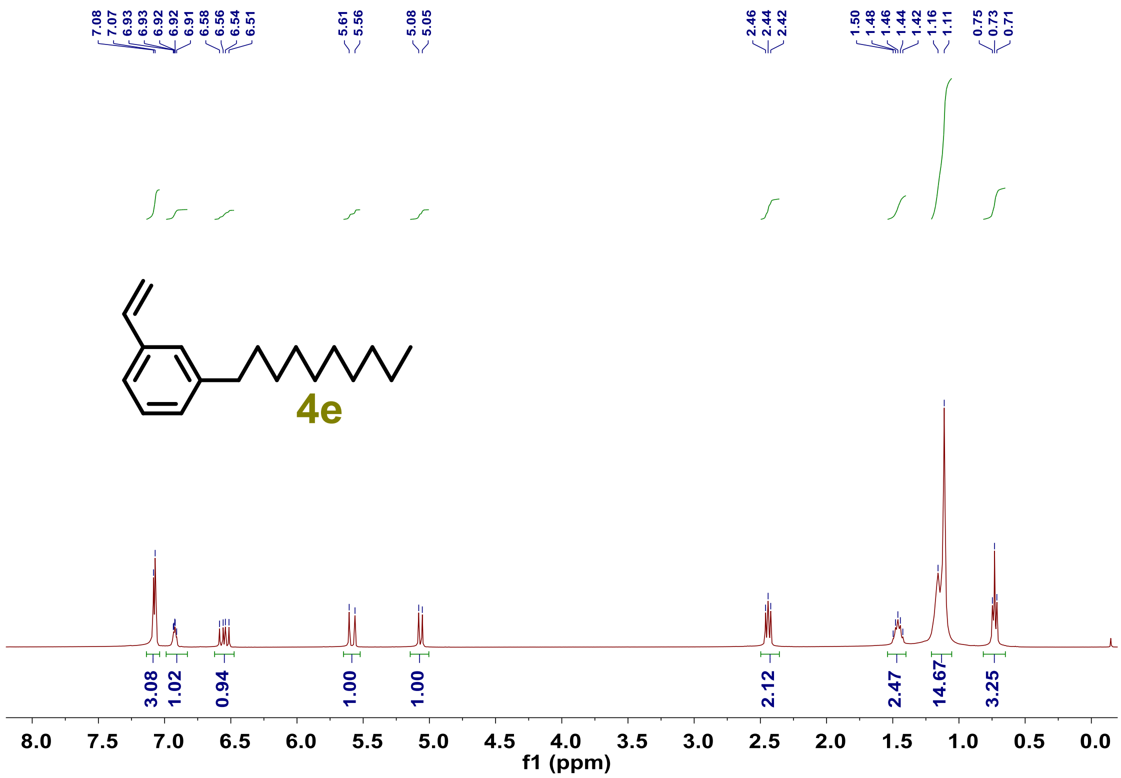

Supplementary Fig. 58 ^{13}C NMR spectrum of 1-decyl-3-vinylbenzene.

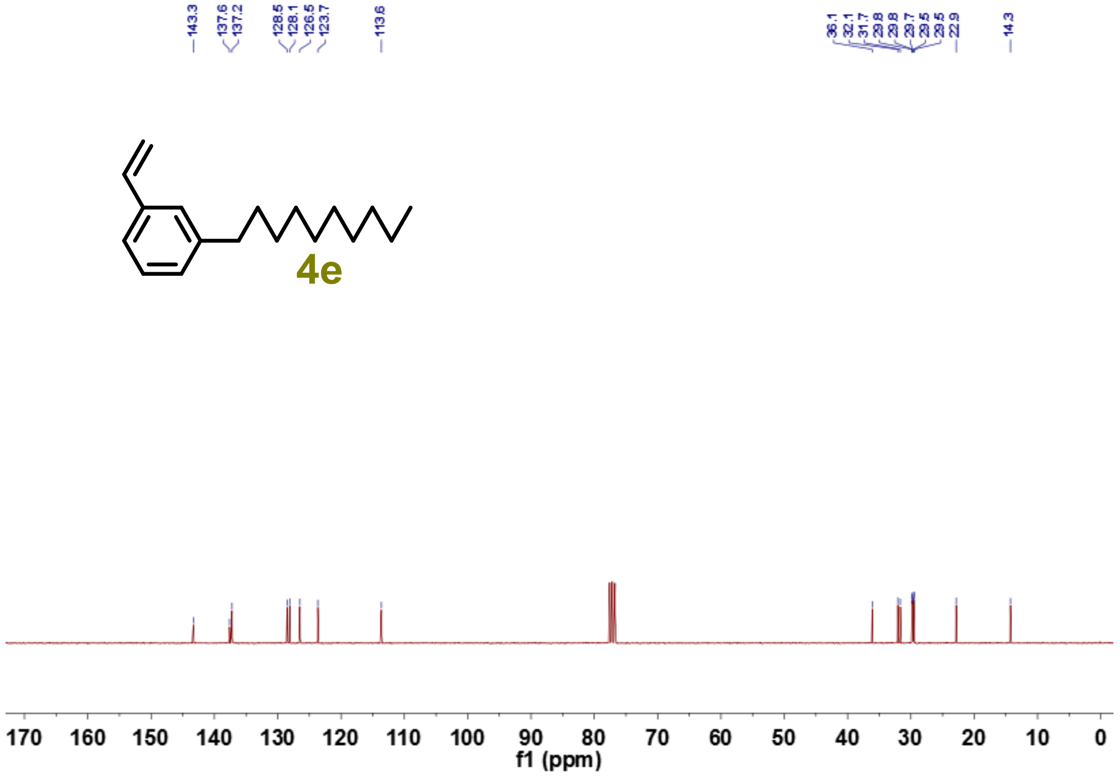

Supplementary Fig. 59 ^{13}C NMR spectrum of 1-decyl-3-vinylbenzene.

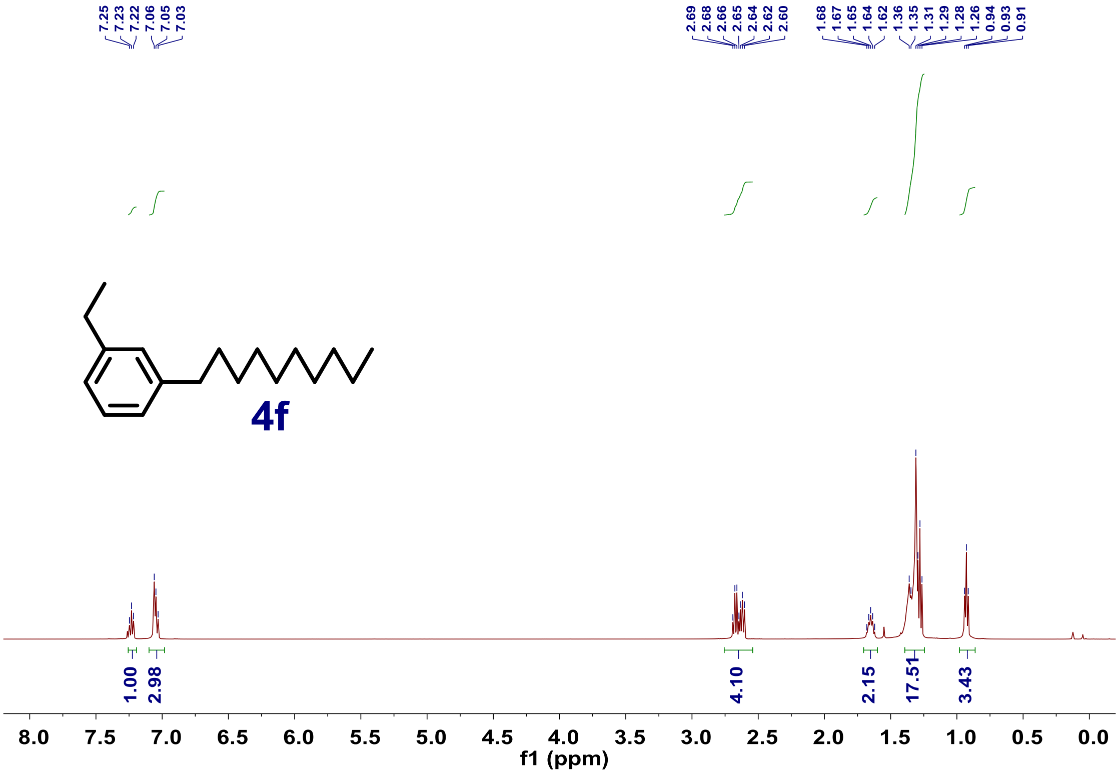

Supplementary Fig. 60 ^{13}C NMR spectrum of 1-decyl-3-ethylbenzene.

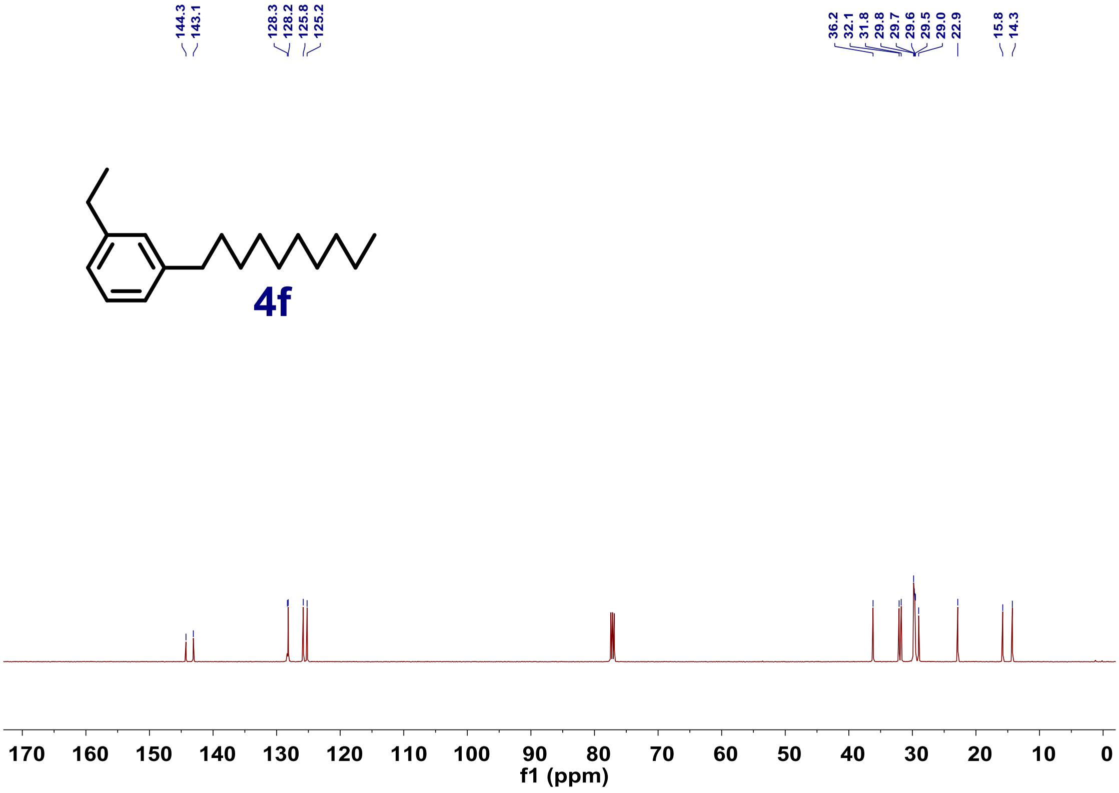

Supplementary Fig. 61 ^{13}C NMR spectrum of 1-decyl-3-ethylbenzene.

Supplementary References

1. Chen, Y., et al. State-of-the-art aluminum porphyrin-based heterogeneous catalysts for the chemical fixation of CO_2 into cyclic carbonates at ambient conditions. *ChemCatChem* **9**, 767–773 (2017).
2. Stolzenberg, A. M., Haymond, G. S. Activation barriers to meso-aryl group rotation in titanyl tetraaryltetrapyrroles. an investigation of the out-of-plane flexibility of hydroporphyrins. *Inorg. Chem.* **41**, 300–308 (2002).
3. Kumar, R., Chaudhary, N., Sankar, M., Maurya, M. R. Electron deficient nonplanar β-octachlorovanadylporphyrin as a highly efficient and selective epoxidation catalyst for olefins. *Dalton Trans.* **44**, 17720–17729 (2015).
4. Chen, P., et al. Binding of propylene oxide to porphyrin- and salen-M(III) cations, where M = Al, Ga, Cr, and Co. *Inorg. Chem.* **44**, 2588–2595 (2005).
5. Han, Y., et al. Singly versus doubly reduced nickel porphyrins for proton reduction: experimental and theoretical evidence for a homolytic hydrogen-evolution reaction. *Angew. Chem. Int. Ed.* **55**, 5457–5462 (2016).
6. Ke, W.-z., et al. Synthesis and properties of a meso-tetraphenylporphyrin and its Zn(II) complex with a dicyanoisophorone liked by double bond in the β pyrrolic position. *Inorg. Chem. Commun.* **14**, 1311–1313 (2011).
7. Bajju, G. D., et al. Synthesis, spectroscopic and biological studies on new zirconium(IV) porphyrins with axial ligand. *Bioinorg. Chem. Appl.*, **2013**, 903616 (2013).
8. Motorina, E. V., Lomova, T. N., Troshin, P. A., Klyuev, M. V. Novel 2'-(pyridin-4-yl)-5'-(pyridin-2-yl)-1'-(pyridin-2-yl)methylpyrrolidinyl[60]fullerene-hydroxyoxo(5,10,15,20-tetraphenyl-21H,23H-porphinato) molybdenum(V) dyads. *Russ. J. Gen. Chem.* **84**, 946–952 (2014).
9. Tanaka, K., et al. Auxiliary-directed oxidation of ursolic acid by 'Ru'-porphyrins: chemical modulation of cytotoxicity against tumor cell lines. *Tetrahedron Lett.* **53**, 1756–1759 (2012).
10. Nakamura, M., et al. Self-assembled molecular gear: A 4:1 complex of Rh(III)Cl tetraarylporphyrin and tetra(p-pyridyl)cavitand. *J. Am. Chem. Soc.* **138**, 12564–12577 (2016).
11. Wang, R.-F., et al. A hydrogen-bonded-supramolecular-polymer-based nanoprobe for ratiometric oxygen sensing in living cells. *Adv. Funct. Mater.* **26**, 5419–5425 (2016).
12. Bichan, N. G., Tyulyaeva, E. Y., Lomova, T. N. Structure and stability of H^+ associates of (5,10,15,20-tetraphenylporphinato)silver(II) in trifluoroacetic acid. *Russ. J. Phys. Chem. A* **88**, 1345–1350 (2014).
13. Azenha, E. G., et al. Heavy-atom effects on metalloporphyrins and polyhalogenated porphyrins. *Chem. Phys.* **280**, 177–190 (2002).
14. Xu, L., Zhai, M.-K., Lu, X.-C., Du, H.-B. A robust indium-porphyrin framework for CO_2 capture and chemical transformation. *Dalton Trans.* **45**, 18730–18736 (2016).
15. Bai, D., Duan, S., Hai, L., Jing, H. Carbon dioxide fixation by cycloaddition with epoxides, catalyzed by biomimetic metalloporphyrins. *ChemCatChem* **4**, 1752–1758 (2012).
16. Santria, A., Fuyuhiro, A., Fukuda, T., Ishikawa, N. Synthesis of a series of heavy Lanthanide(III) monoporphyrinato complexes with tetragonal symmetry. *Inorg. Chem.* **56**, 10625–10632 (2017).

17. Motorina, E. V., Lomova, T. N. Hydroxyoxo(5,10,15,20-tetraphenylporphinato)tungsten(V) as a receptor for foodstuff and drug components. Thermodynamics of supramolecular complexation. *Russ. J. Inorg. Chem.* **55**, 727–733 (2010).
18. Wang, J.-C., et al. Enantioselective intramolecular carbene C-H insertion catalyzed by a chiral iridium(III) complex of D_4-symmetric porphyrin ligand. *ACS Catal.* **3**, 1144–1148 (2013).
19. Borisov, S. M., Klimant, I. Efficient metalation in diphenyl ether - A convenient route to luminescent platinum(II) complexes. *Dyes Pigm.* **83**, 312–316 (2009).
20. Dixon, I. M., Collin, J.-P., Sauvage, J.-P., Flamigni, L. Porphyrinic dyads and triads assembled around iridium(III) bis-terpyridine: Photoinduced electron transfer processes. *Inorg. Chem.* **40**, 5507–5517 (2001).
21. Gomes, M. L., et al. Synthesis and characterization of bismuth(III) and antimony(V) porphyrins: high antileishmanial activity against antimony-resistant parasite. *J. Biol. Inorg. Chem.* **20**, 771–779 (2015).
