# Supplementary material for: A versatile route to fabricate single atom catalysts with high chemoselectivity and regioselectivity in hydrogenation
Source: Nat Commun. 2019 Aug 14;10:3663. doi: 10.1038/s41467-019-11619-6 (PMC6694111; doi:10.1038/s41467-019-11619-6)
Supplement: Supplementary file 2 — Peer Review [file 41467_2019_11619_MOESM2_ESM.pdf]

Reviewers' comments:

Reviewer #1 (Remarks to the Author):

The authors report a synthesis that involves a copolymerization of a metalloporphyrin with tetraphenyly porphyrin, and subsequent pyrolysis, to yield a single atom catalyst where Pt is bound to Nitrogen and Carbon in the support. While pyrolysis is generally used to make such M-N-C catalysts, what is different here is that there is no additional step beyond the pyrolysis. The simplicity of the synthesis makes this of interest to generate carbon supported single atom catalysts. But the drawback is that the concentrations of the metal remain low. The authors further demonstrate the versatility of the synthesis by preparing single atom materials with a variety of metals.

I have concerns about some of the results presented here, and they need to be addressed before the work is suitable for publication.

1) Figures 1 – 4 show AC-STEM images where the atomically dispersed metal atoms are circled in yellow. My concern is that there appear to be too many such circled atoms. The authors need to check their loading and estimate how many they would expect to see per nm2. The limited depth of focus of the AC-STEM image ensures that the atoms in focus come from a plane that is perpendicular to the viewing direction. Hence a mass balance can be achieved by simply counting the number of atoms visible over a certain area of the image. A recently published paper on Pt/CeO2 (Kunwar et al., ACS Catal, DOI: 10.1021/acscatal.8b04885) demonstrates images of high concentrations of single atom Pt (1 atom/nm2) where counting of the single atoms agrees with the bulk and surface analysis of these atoms. Such a mass balance is necessary to validate these images. I get the feeling that there are too many bright dots being circled, leading me to the suspicion that these are bright dots are caused by fluctuations in the density of the sample in the viewing direction, rather than isolated single atoms. Hence, to establish the validity of this measurement, besides the counting suggested above, the authors need to present in the supporting information some pictures of the sample without the single atoms (the support), so the reader can assess the validity of the evidence presented here for establishing the single atom nature of the catalyst.

2) When performing catalysis, especially when the nanoparticles are more active, the authors must present characterization of the catalyst after reaction, to demonstrate that the structure is maintained.

3) During the selective hydrogenation, it appears that the single atom catalyst is much less active, but highly selective. It is expected that selectivity for such reactions is influenced by conversion, since at higher conversion the molecule has a chance to get hydrogenated at multiple sites. The authors need to present results for selectivity as a function of conversion. It is not enough to state that conversion was less than 20% since that does not tell us the conversion for each sample.

Reviewer #2 (Remarks to the Author):

In this work, He and co-authors used a precursor-dilution strategy to synthesize a series of single atom catalysts with different densities. The key novelty of this work is a demonstration of a general approach for the synthesis of 24 different kinds of SACs. In addition, the authors also demonstrate that Pt1/N-C SAC shows a high chemo- and regio-selectivity in hydrogenation. This work is potentially suitable for publication in Nature communications journals. However, the following points have to be addressed.

[1] In addition to STEM images, the authors need to provide EXAFS data for all the SACs reported in this work. Since the central claim of this work is the synthesis of a library of SACs. The experimental evidence has to be convincing.

[2] The authors have to comment the atomic structures of all the SACs reported in this work. Do all the SACs have the same atomic structure? The authors need to provide evidence.
[3] The authors have shown a successful synthesis of bimetallic Pt and Sn SACs site. Does it apply for other metal species?
[4] The stability of Pt1/N-C SAC for the reaction shall be evaluated.

Response to the Reviewers' Comments:

Reviewer #1:

Comment 1: *Figures 1–4 show AC-STEM images where the atomically dispersed metal atoms are circled in yellow. My concern is that there appear to be too many such circled atoms. The authors need to check their loading and estimate how many they would expect to see per nm^2. The limited depth of focus of the AC-STEM image ensures that the atoms in focus come from a plane that is perpendicular to the viewing direction. Hence a mass balance can be achieved by simply counting the number of atoms visible over a certain area of the image. A recently published paper on Pt/CeO_2 (Kunwar et al., ACS Catal, DOI: 10.1021/acscatal.8b04885) demonstrates images of high concentrations of single atom Pt (1 atom/nm^2) where counting of the single atoms agrees with the bulk and surface analysis of these atoms. Such a mass balance is necessary to validate these images. I get the feeling that there are too many bright dots being circled, leading me to the suspicion that these are bright dots are caused by fluctuations in the density of the sample in the viewing direction, rather than isolated single atoms. Hence, to establish the validity of this measurement, besides the counting suggested above, the authors need to present in the supporting information some pictures of the sample without the single atoms (the support), so the reader can assess the validity of the evidence presented here for establishing the single atom nature of the catalyst.*

Response 1: We thank the reviewer's comment. First, AC HAADF-STEM images for the N-C support without any metal atoms (prepared with the same method but from pure porphyrin without metal) were shown in Fig. R1. Clearly, there were no bright dots on N-C support, which excluded the possibility that "the bright dots were caused by fluctuations in the density of the sample in the viewing direction". Second, following the reviewer's suggestion, we also evaluated the mass balance (D. Kunwar et al., ACS Catal. 2019, 9, 3978–3990, cited as Ref. 40 in the revised manuscript): 1. Estimating the metal atom densities based on the results of ICP (for metal loading) and BET areas (denoted as Density1); 2. Estimating the metal atom densities based on the number of bright dots and the area (~200 nm^2) of the AC HAADF-STEM images (denoted as Density2); 3. Comparing the two results. The results were shown in Table R1. Indeed, Density2 was several times as high as Density1. However, this could be due to two reasons. 1. the mass balance above is based on an assumption that the depth of focus of the AC HAADF-STEM is limited to only one layer. Actually, according to the reference (P. D. Nellist et al., Microsc. Microanal. 2008, 14, 82–88), the depth of focus of the AC HAADF-STEM is mainly determined by convergence semi-angle of the condenser lens, and for the ARM 200F we used, the depth of focus is about 10 nm. In the D. Kunwar et al.'s work, the thickness of the

underlying support of CeO_2 is probably ~10 nm (L. Wu et al., Phys. Rev. B 2004, 69, 125415; I. I. Soykal et al., ACS Catal. 2012, 2, 2335–2348), so the bright dots on the support should be ascribed to the metal atoms on only one layer of CeO_2 (see Fig. R2). While according to the literature (M. Allen et al., Chem. Rev. 2010, 110, 132–145), the thickness of single layer of graphene is about 0.6 nm, so it is reasonable to ascribe the bright dots in our images to the metal atoms on several layers of nitrogen-doped carbon (see Fig. R2). Here we also give some previous reports to support our speculation (see Table R2) that Density1 and Density2 of metal oxides supported SACs are always similar while those of carbon supported SACs are not. 2. Also, as the localized observation method, the images selected cannot be regarded as statistic of the overall situation.

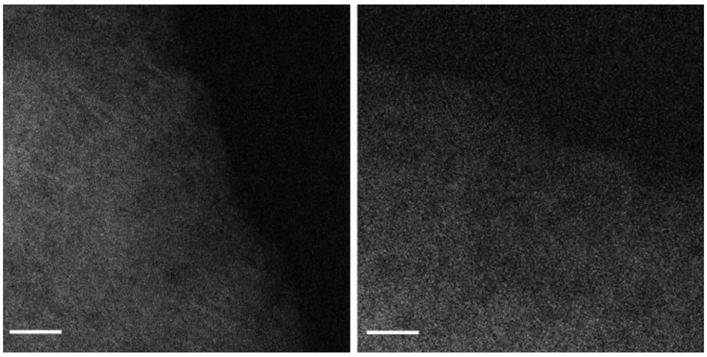

Fig. R1 AC HAADF-STEM of N-C. Scale bar, 2 nm.

Table R1. Metal contents, BET surface areas, Density1, Density2, and their ratios for M_1/N-C catalysts

Sample	M (wt%)	BET (m^2/g)	Density1 (atom/nm^2) [a]	Density2 (atom/nm^2) [b]	Density2/ Density1
Pt_1/N-C(1:20)	0.73	656	0.034	0.340	10.0
Pt_1/N-C	0.43	595	0.022	0.175	8.0
Pt_1/N-C(1:80)	0.21	632	0.010	0.085	8.5
Pt_1/N-C(1:320)	0.06	644	0.003	0.025	8.3
Ti_1/N-C	0.12	587	0.026	0.050	1.9
V_1/N-C	0.19	656	0.034	0.080	2.4
Cr_1/N-C	0.10	792	0.015	0.060	4.0
Mn_1/N-C	0.07	790	0.010	0.060	6.0
Fe_1/N-C	0.18	784	0.025	0.060	2.4
Co_1/N-C	0.20	834	0.024	0.045	1.9
Ni_1/N-C	0.22	677	0.033	0.080	2.4
Cu_1/N-C	0.21	746	0.027	0.045	1.7
Ga_1/N-C	0.06	841	0.006	0.040	6.7
Zr_1/N-C	0.05	524	0.006	0.050	8.3
Mo_1/N-C	0.15	567	0.017	0.090	5.3
Ru_1/N-C	0.10	877	0.007	0.050	7.1
Rh_1/N-C	0.08	869	0.005	0.075	15.0
Pd_1/N-C	0.30	682	0.025	0.075	3.0

Ag_1/N-C	0.06	799	0.004	0.055	13.8
Cd_1/N-C	0.32	629	0.027	0.115	4.3
In_1/N-C	0.09	683	0.007	0.045	6.4
Sn_1/N-C	0.43	426	0.051	0.040	0.8
Er_1/N-C	0.06	793	0.003	0.050	16.7
W_1/N-C	0.27	443	0.020	0.095	4.8
Ir_1/N-C	0.38	837	0.014	0.140	10.0
Au_1/N-C	0.19	783	0.007	0.170	24.3
Bi_1/N-C	0.08	743	0.003	0.050	16.7
Pt_1-Sn_1/N-C	0.48-0.35	724	0.045	0.140	3.1

[a] Density1 = (Metal loading × N_A)/(Molar mass × BET surface area). N_A is Avogadro's constant. [b] Density2 = (the number of bright dots) / (AC HAADF-STEM image area).

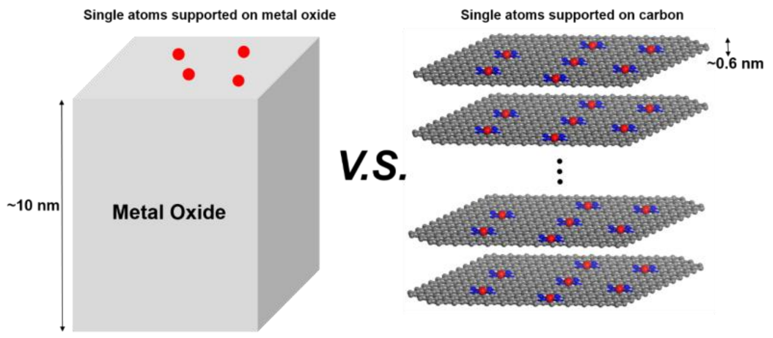

Fig. R2 Illustration of single atoms catalyst supported on metal oxides or carbon.

Table R2 Density1 and Density2 of the previous literatures

Support	Catalyst	Density1 (atom/nm^2) [a]	Density2 (atom/nm^2) [b]	Density2/ Density1	Reference
Metal oxides	Pt_1/CeO_2	0.99	1.00	1.0	D. Kunwar et al., ACS Catal. 2019, 9, 3978–3990
	Pt_1/MnO_2	0.34	0.38	1.1	L. Kuai et al., Chem. Mater. 2018, 30, 5534–5538
	Pt_1/Fe_2O_3	1.20	1.00	0.8	S. Duan et al., Nanotechnology, 2018, 29, 204002
	Pt_1/FeO_x	0.09	0.07	0.8	B. Qiao et al., Nat. Chem. 2011, 3, 634–641
Carbon	Pt_1/N-C	0.086	2.033	23.6	Y. Zhu et al., ACS Catal. 2018, 8, 10004–10011
	Pt_1/N-C	0.064	0.281	4.4	Z. Chen, et al., Adv. Funct. Mater. 2017, 27, 1605785
	Pt_1/N-C	0.011	0.143	13.0	J. Liu et al., Nat. Commun. 2017, 8, 15938
	Pt_1/N-C	0.008	0.319	39.9	R. Kamai et al., Angew. Chem. Int. Ed. 2016, 55, 13184–13188
	Pt_1/N-C	0.022	0.175	8.0	Our work

[a] Density1 = (Metal loading × N_A)/(Molar mass × BET surface area). N_A is Avogadro's constant. [b] Density2 = (the number of bright dots) / (AC HAADF-STEM image area).

Comment 2: *When performing catalysis, especially when the nanoparticles are more active, the authors must present characterization of the catalyst after reaction, to demonstrate that the structure is maintained.*

Response 2: We thank the reviewer for the suggestion. As shown in Fig. R3, no Pt nanoparticles or nanoclusters were found in TEM and STEM images, and the corresponding AC HAADF-STEM images revealed that the Pt species maintained the atomically dispersed states after the hydrogenation reactions. And we have added the discussion in the revised manuscript (Supplementary Fig. 39).

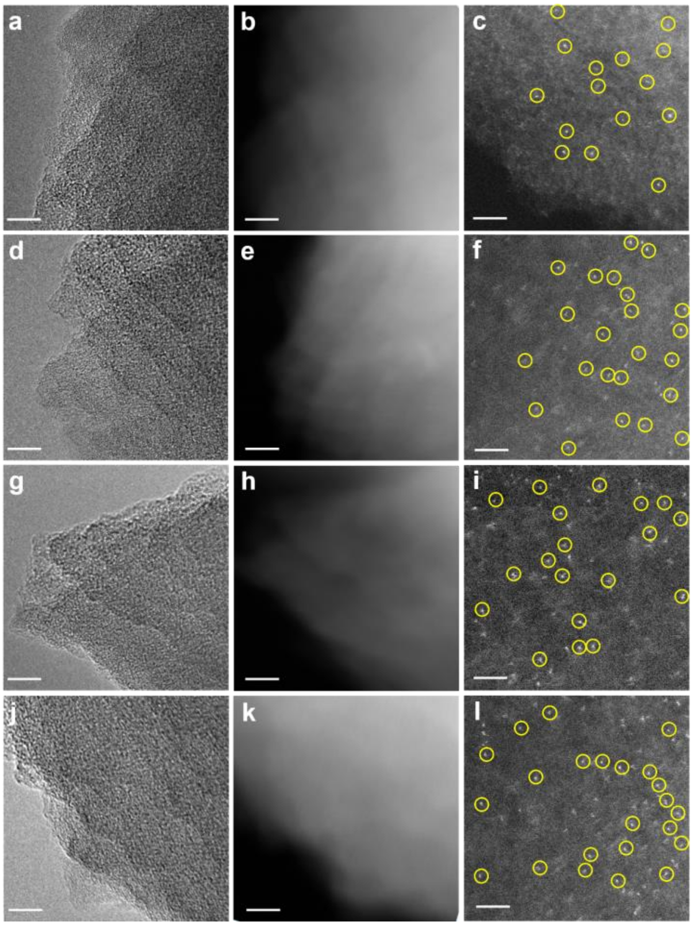

Fig. R3 TEM, STEM, and AC HAADF-STEM images of Pt_1/N-C after 5 catalytic runs of hydrogenation of **a–c** 1-nitro-4-ethynylbenzene, **d–f** 1-ethynyl-4-vinylbenzene, **g–i** 1-ethynyl-4-(phenylethynyl)benzene, and **j–l** 1-(dec-1-yn-1-yl)-3-ethynylbenzene, respectively. Scale bar, 10 nm for TEM/STEM images, 2 nm for AC HAADF-STEM images.

Comment 3: *During the selective hydrogenation, it appears that the single atom catalyst is much less active, but highly selective. It is expected that selectivity for such reactions is influenced by conversion, since at higher conversion the molecule has a chance to get hydrogenated at multiple sites. The authors need to present results for selectivity as a function*

of conversion. It is not enough to state that conversion was less than 20% since that does not tell us the conversion for each sample.

Response 3: Following the suggestion of the reviewer, we did the selective hydrogenation reaction of several substrates on Pt_1/N-C of longer reaction time (Fig. R4). When the conversions increased to ~100%, the selectivities to the target products still maintained >96%, which clearly indicated that Pt_1/N-C was highly selective in these reactions. And we have added the results in the revised manuscript (Supplementary Fig. 36).

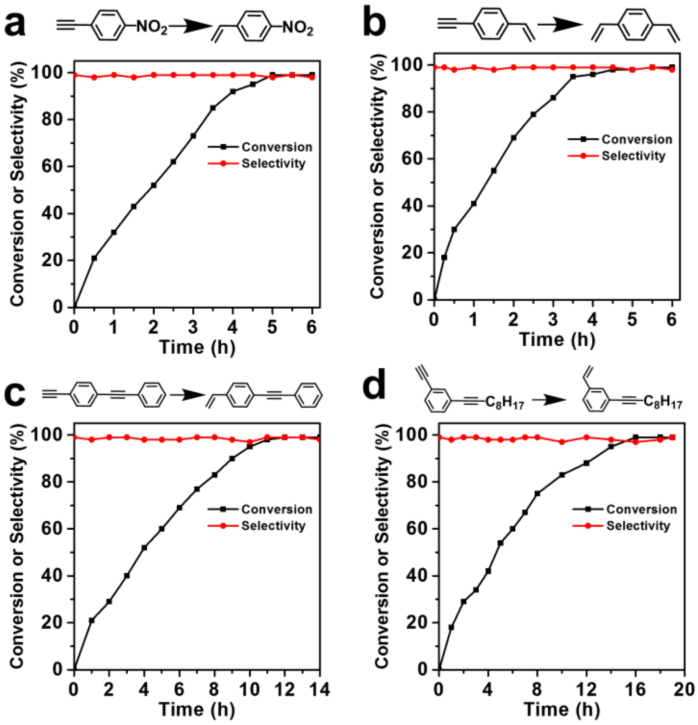

Fig. R4 Selective hydrogenation of **a** 1-nitro-4-ethynylbenzene, **b** 1-ethynyl-4-vinylbenzene, **c** 1-ethynyl-4-(phenylethynyl)benzene, and **d** 1-(dec-1-yn-1-yl)-3-ethynylbenzene on Pt_1/N-C. Reaction condition: substrate (0.5 mmol), Pt_1/N-C (Pt:substrate = 1:1200, mol:mol), methanol (2.0 mL), H_2 (1.0 MPa). Reaction temperature: **a** and **b** 50 °C; **c** and **d** 80 °C.

Reviewer #2:

Comment 1: *In addition to STEM images, the authors need to provide EXAFS data for all the SACs reported in this work. Since the central claim of this work is the synthesis of a library of SACs. The experimental evidence has to be convincing.*

Response 1: We thank the reviewer for the suggestion. Although with very tight beamtime schedule, we still tried out best to measure SACs as many as we can. As shown in Fig. R5, the EXAFs profiles of 17 SACs covering noble metals (Ru, Rh, Pd, Ir, and Pt), transition metals (V, Cr, Mn, Fe, Co, Ni, Cu, Zr, Mo, and W), and main group metals (Ga and Sn) were provided. Gratifyingly all of them revealed the absence of the metal-metal bond, which clearly demonstrated the atomically dispersed nature of the metal species, in good agreement with the results of AC HAADF-STEM. It is very difficult to get the EXAFS results for the other 7 samples (Ti, Ag, Cd, In, Er, Au, and Bi) mainly because the metal loadings were too low to obtain the high quality EXAFS results. And we have added the discussion in the revised manuscript (Supplementary Fig.29).

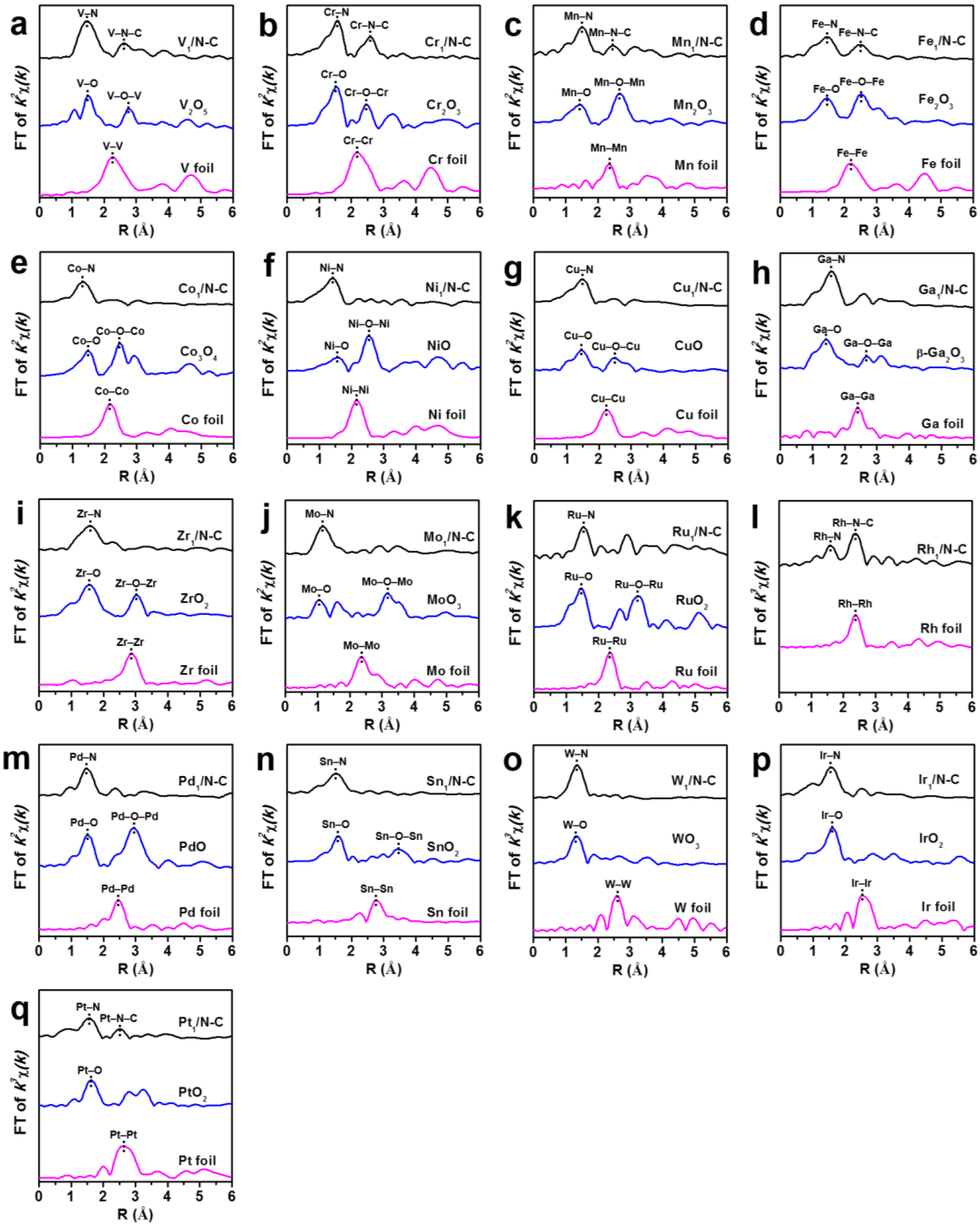

Fig. R5 EXAFS data for M_1/N-C, corresponding metal oxides and metal foils (M= V, Cr, Mn, Fe, Co, Ni, Cu, Ga, Zr, Mo, Ru, Rh, Pd, Sn, W, Ir, and Pt, respectively).

Comment 2: *The authors have to comment the atomic structures of all the SACs reported in this work. Do all the SACs have the same atomic structure? The authors need to provide evidence.*

Response 2: We thank the reviewer's comment. We fitted the EXAFs profiles of 17 SACs. As shown in Fig. R6 and Table R3. The coordination numbers of the metal centers with surrounding N atoms extracted from the EXAFs result was about 4 (Table R3; M = Cr, Mn, Fe, Co, Ni, Cu, Ga, Zr, Rh, Pd, Sn, W, Ir, and Pt), indicating that most of the SACs possessed a MN_4-like structure inherited from the precursors of metalloporphyrins (X. Fang et al., Adv. Mater. 2018, 30, 1705112; Y. Han et al., J. Am. Chem. Soc. 2017, 139, 17269–17272). However, due to the extremely low metal loadings, the signal-to-noise ratios of some SACs (V, Mo and Ru) were not satisfactory, leading to significant ghost-peaks or peak shifting in R space spectrum so that fitting results became unreliable, making it difficult to determine their atomic structures only based on the EXAFS results. However, combining EXAFS and AC HAADF-STEM results together, it is easy to conclude the atomically dispersed structure of all the samples in the manuscript. We have made this clear in the revised manuscript.

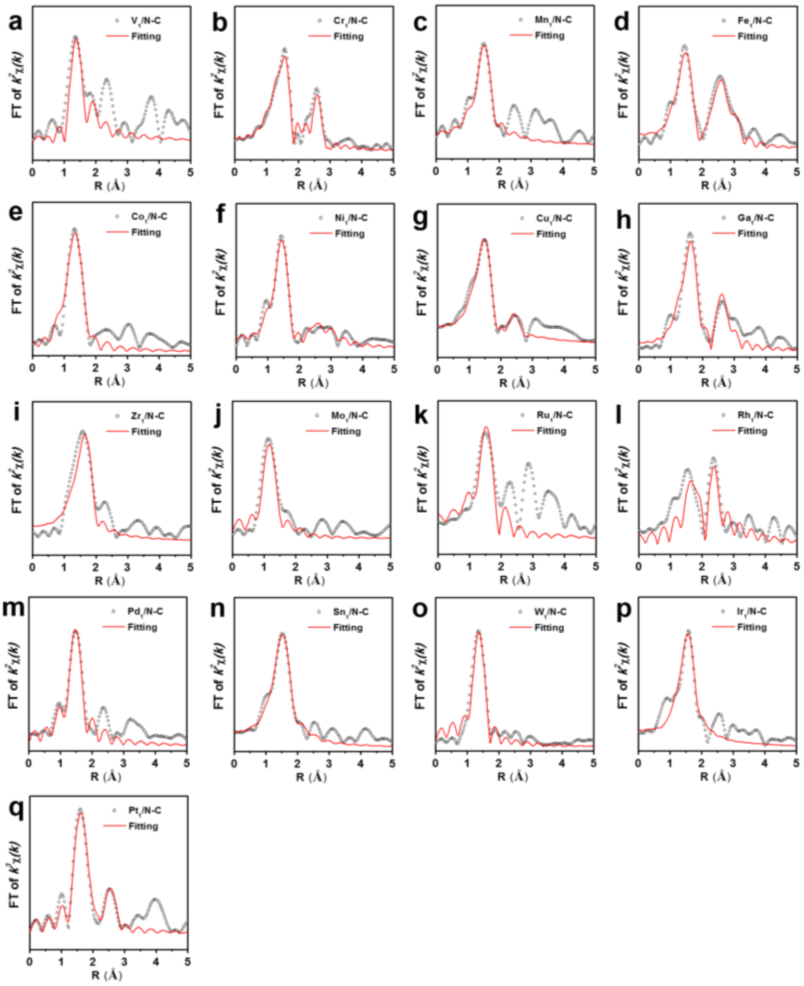

Fig. R6 EXAFS fitting for M_1/N-C (M= V, Cr, Mn, Fe, Co, Ni, Cu, Ga, Zr, Mo, Ru, Rh, Pd, Sn, W, Ir, and Pt, respectively).

Supplementary Table R3. Structural parameters of EXAFS fitting for the M_1/N-C

Sample	Shell	C.N. [a]	σ^2 (× 10^{-3} Å^2) [b]	E_0 (eV) [c]	R (Å) [d]
V_1/N-C	V–N	1.8	6.1	30.9	1.63±0.06
Cr_1/N-C	Cr–N	2.7	3.0	-6.2	1.95±0.04
	Cr–N–C	4.1	0.3	-6.2	3.42±0.07
Mn_1/N-C	Mn–N	3.7	3.5S	1.3	2.03±0.08
Fe_1/N-C	Fe–N/O	6.6	13.0	-0.5	2.02±0.13
	Fe–N–C	4.3	6.8	-0.5	2.90±0.15
Co_1/N-C	Co–N/O	5.0	8.6	-17.5	1.89±0.09
Ni_1/N-C	Ni–N	3.0	5.5	-2.0	1.85±0.05
	Ni–N–C	1.0	1.8	-2.0	2.72±0.10
Cu_1/N-C	Cu–N	3.0	5.5	0.4	1.92±0.01
	Cu–N–C	4.0	7.3	6.7	2.90±0.05
Ga_1/N-C	Ga–N	3.3	5.4	4.1	2.00±0.12
	Ga–N–C	2.4	3.4	4.1	3.07±0.14
Zr_1/N-C	Zr–N	3.9	6.5	-1.0	2.18±0.01
Mo_1/N-C	Mo–N	1.4	1.0	1.6	1.82±0.43
Ru_1/N-C	Ru–N	1.1	5.1	6.5	2.10±0.06
Rh_1/N-C	Rh–N	2.4	0.1	12.0	2.16±0.43
	Rh–N–C	5.8	0.1	12.0	3.16±0.35
Pd_1/N-C	Pd–N	2.3	2.8	-1.2	1.96±0.02
Sn_1/N-C	Sn–N	3.8	7.5	8.8	2.03±0.01
W_1/N-C	W–N	3.5	1.2	9.7	1.73±0.03
Ir_1/N-C	Ir–N	3.5	6.9	4.9	1.98±0.02
Pt_1/N-C	Pt–N	3.4	3.0	0.7	2.03±0.02
	Pt–N–C	2.2	2.5	5.4	2.98±0.04

[a] C.N.: coordination number; [b] σ^2: Debye-waller factors; [c] E_0: the inner potential correction. [d] R: bond distance;

Comment 3: *The authors have shown a successful synthesis of bimetallic Pt and Sn SACs site. Does it apply for other metal species?*

Response 3: We thank the reviewer for the suggestion. Our strategy is of great flexibility and here we give another example, the successful synthesis of trimetallic SACs (Pt_1-Sn_1-Cu_1/N-C, Fig. R7). With the molar ratio of PtTPP:SnTPP:CuTPP:TPP (1:1:1:40) and the same synthesis procedure as previously described, the nitrogen-doped porous carbon based materials with 0.45 wt% Pt loading, 0.28 wt% Sn loading and 0.18 wt% Cu loading were obtained. This ratio of the metal loading for Pt, Sn and Cu (2.5:1.6:1) was in good agreement with the nominal ratio

(3.0:1.8:1), based on the molar ratio of PtTPP:SnTPP:CuTPP (1:1:1) and atomic weight ratio of Pt:Sn:Cu (195.1:118.7:63.5). No nanoparticles were observed in TEM/STEM images and no corresponding XRD peaks emerged. Additionally, in the AC HAADF-STEM image, the homogeneously distributed bright dots tagged by yellow circles can be attributed to Pt, or Sn or Cu atoms. Corresponding element mapping analysis of Pt_1-Sn_1-Cu_1/N-C revealed that Pt, Sn and Cu species were homogeneously distributed. Combined all the results above, it can be concluded that all three metal species were atomically dispersed on the porous carbon matrix, i.e. the synthesis of Pt_1-Sn_1-Cu_1/N-C was achieved. Surely, we will report the strategy for multi-metal SACs construction in our future publication.

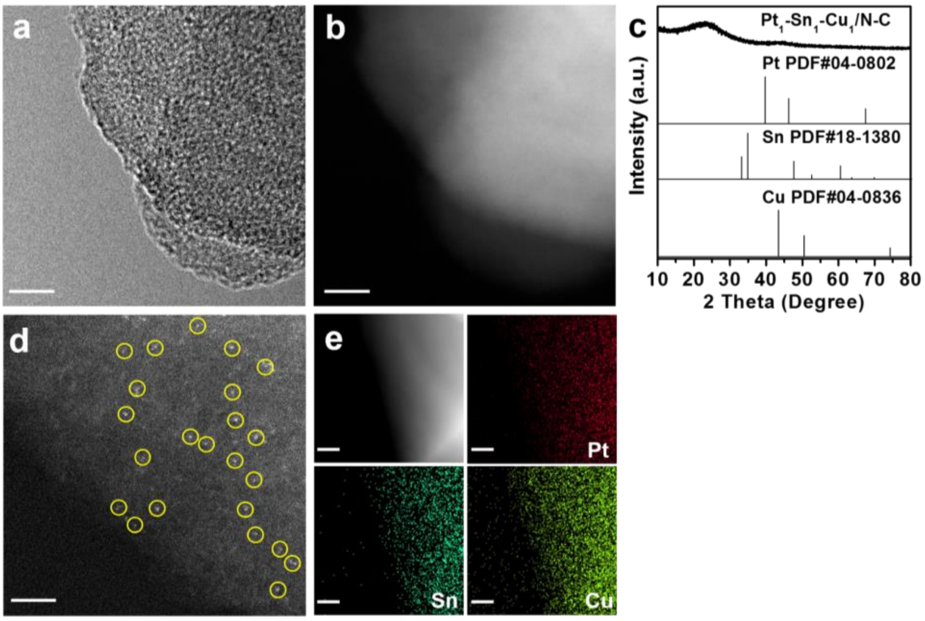

Fig. R7 **a** TEM and **b** STEM of Pt_1-Sn_1-Cu_1/N-C. Scale bar, 10 nm. **c** XRD pattern of Pt_1-Sn_1-Cu_1/N-C. **d** AC HAADF-STEM of Pt_1-Sn_1-Cu_1/N-C. Scale bar, 2 nm. **e** element mapping of Pt_1-Sn_1-Cu_1/N-C. Scale bar, 100 nm.

Comment 4: *The stability of Pt_1/N-C SAC for the reaction shall be evaluated.*

Response 4: We thank the reviewer for the nice suggestion. The recycling experiments of Pt_1/N-C catalysts in four representative hydrogenation reactions were conducted for five runs (Fig. R8). Clearly, no obvious decline in catalytic activity and selectivity were observed. Combined with the results that the structure of the catalysts were well maintained after reactions (see Response 2 for Reviewer 1), we concluded that Pt_1/N-C catalyst is stable under our reaction conditions. We have added the result in the revised manuscript (Supplementary Fig. 38).

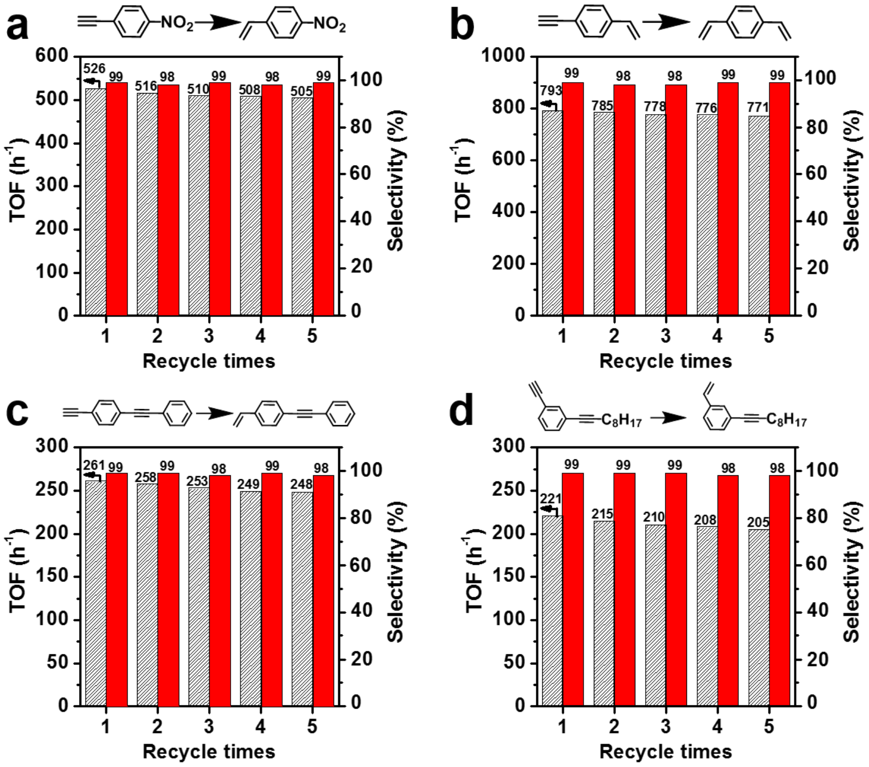

Fig. R8 Catalytic performance of Pt_1/N-C in hydrogenation of **a** 1-nitro-4-ethynylbenzene, **b** 1-ethynyl-4-vinylbenzene, **c** 1-ethynyl-4-(phenylethynyl)benzene, and **d** 1-(dec-1-yn-1-yl)-3- ethynylbenzene for 5 catalytic runs. Reaction condition: substrate (0.5 mmol), catalyst (Pt:substrate = 1:1200, mol:mol), methanol (2.0 mL), H_2 (1.0 MPa). Reaction temperature: **a** and **b** 50 °C; **c** and **d** 80 °C. All the conversions were maintained at ~20%.

REVIEWERS' COMMENTS:

Reviewer #1 (Remarks to the Author):

The authors have addressed most comments, hence the manuscript is suitable for publication. But they have not adequately considered the counting of atoms per unit area in their AC-STEM images. I have provided more details in my attachment.

Reviewer #2 (Remarks to the Author):

The authors have addressed my comments and made the revisions accordingly. Therefore, I recommend the publication of this paper.

Response to the Reviewers' Comments:

Reviewer #1:

Comment 1: *Figures 1–4 show AC-STEM images where the atomically dispersed metal atoms are circled in yellow. My concern is that there appear to be too many such circled atoms. The authors need to check their loading and estimate how many they would expect to see per nm^2. The limited depth of focus of the AC-STEM image ensures that the atoms in focus come from a plane that is perpendicular to the viewing direction. Hence a mass balance can be achieved by simply counting the number of atoms visible over a certain area of the image. A recently published paper on Pt/CeO_2 (Kunwar et al., ACS Catal, DOI: 10.1021/acscatal.8b04885) demonstrates images of high concentrations of single atom Pt (1 atom/nm^2) where counting of the single atoms agrees with the bulk and surface analysis of these atoms. Such a mass balance is necessary to validate these images. I get the feeling that there are too many bright dots being circled, leading me to the suspicion that these are bright dots are caused by fluctuations in the density of the sample in the viewing direction, rather than isolated single atoms. Hence, to establish the validity of this measurement, besides the counting suggested above, the authors need to present in the supporting information some pictures of the sample without the single atoms (the support), so the reader can assess the validity of the evidence presented here for establishing the single atom nature of the catalyst.*

Response 1: We thank the reviewer's comment. First, AC HAADF-STEM images for the N-C support without any metal atoms (prepared with the same method but from pure porphyrin without metal) were shown in Fig. R1. Clearly, there were no bright dots on N-C support, which excluded the possibility that "the bright dots were caused by fluctuations in the density of the sample in the viewing direction". Second, following the reviewer's suggestion, we also evaluated the mass balance (D. Kunwar et al., ACS Catal. 2019, 9, 3978–3990, cited as Ref. 40 in the revised manuscript): 1. Estimating the metal atom densities based on the results of ICP (for metal loading) and BET areas (denoted as Density1); 2. Estimating the metal atom densities based on the number of bright dots and the area (~200 nm^2) of the AC HAADF-STEM images (denoted as Density2); 3. Comparing the two results. The results were shown in Table R1. Indeed, Density2 was several times as high as Density1. However, this could be due to two reasons. 1. the mass balance above is based on an assumption that the depth of focus of the AC HAADF-STEM is limited to only one layer. Actually, according to the reference (P. D. Nellist et al., Microsc. Microanal. 2008, 14, 82–88), the depth of focus of the AC HAADF-STEM is mainly determined by convergence semi-angle of the condenser lens, and for the ARM 200F we used, the depth of focus is about

10 nm. In the D. Kunwar et al.'s work, the thickness of the underlying support of CeO_2 is probably ~10 nm (L. Wu et al., Phys. Rev. B 2004, 69, 125415; I. I. Soykal et al., ACS Catal. 2012, 2, 2335–2348), so the bright dots on the support should be ascribed to the metal atoms on only one layer of CeO_2 (see Fig. R2). While according to the literature (M. Allen et al., Chem. Rev. 2010, 110, 132–145), the thickness of single layer of graphene is about 0.6 nm, so it is reasonable to ascribe the bright dots in our images to the metal atoms on several layers of nitrogen-doped carbon (see Fig. R2). Here we also give some previous reports to support our speculation (see Table R2) that Density1 and Density2 of metal oxides supported SACs are always similar while those of carbon supported SACs are not. 2. Also, as the localized observation method, the images selected cannot be regarded as statistic of the overall situation.

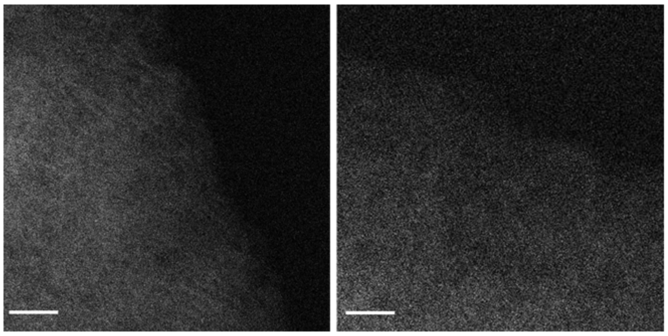

Fig. R1 AC HAADF-STEM of N-C. Scale bar, 2 nm.

Table R1. Metal contents, BET surface areas, Density1, Density2, and their ratios for M_1/N-C catalysts

Sample	M (wt%)	BET (m^2/g)	Density1 (atom/nm^2) [a]	Density2 (atom/nm^2) [b]	Density2/ Density1
Pt_1/N-C(1:20)	0.73	656	0.034	0.340	10.0
Pt_1/N-C	0.43	595	0.022	0.175	8.0
Pt_1/N-C(1:80)	0.21	632	0.010	0.085	8.5
Pt_1/N-C(1:320)	0.06	644	0.003	0.025	8.3
Ti_1/N-C	0.12	587	0.026	0.050	1.9
V_1/N-C	0.19	656	0.034	0.080	2.4
Cr_1/N-C	0.10	792	0.015	0.060	4.0
Mn_1/N-C	0.07	790	0.010	0.060	6.0
Fe_1/N-C	0.18	784	0.025	0.060	2.4
Co_1/N-C	0.20	834	0.024	0.045	1.9
Ni_1/N-C	0.22	677	0.033	0.080	2.4
Cu_1/N-C	0.21	746	0.027	0.045	1.7
Ga_1/N-C	0.06	841	0.006	0.040	6.7
Zr_1/N-C	0.05	524	0.006	0.050	8.3
Mo_1/N-C	0.15	567	0.017	0.090	5.3
Ru_1/N-C	0.10	877	0.007	0.050	7.1

Rh_1/N-C	0.08	869	0.005	0.075	15.0
Pd_1/N-C	0.30	682	0.025	0.075	3.0
Ag_1/N-C	0.06	799	0.004	0.055	13.8
Cd_1/N-C	0.32	629	0.027	0.115	4.3
In_1/N-C	0.09	683	0.007	0.045	6.4
Sn_1/N-C	0.43	426	0.051	0.040	0.8
Er_1/N-C	0.06	793	0.003	0.050	16.7
W_1/N-C	0.27	443	0.020	0.095	4.8
Ir_1/N-C	0.38	837	0.014	0.140	10.0
Au_1/N-C	0.19	783	0.007	0.170	24.3
Bi_1/N-C	0.08	743	0.003	0.050	16.7
Pt_1-Sn_1/N-C	0.48-0.35	724	0.045	0.140	3.1

[a] Density1 = (Metal loading × N_A)/(Molar mass × BET surface area). N_A is Avogadro's constant. [b] Density2 = (the number of bright dots) / (AC HAADF-STEM image area).

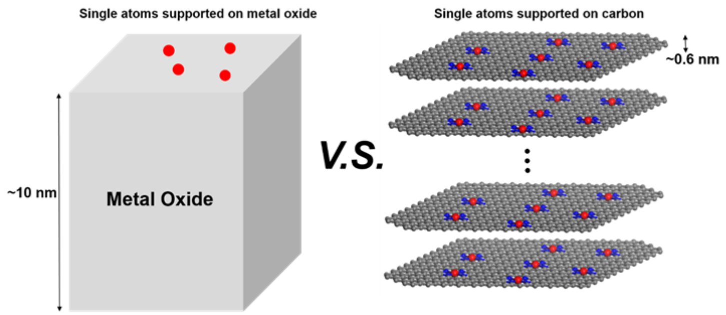

Fig. R2 Illustration of single atoms catalyst supported on metal oxides or carbon.

Table R2 Density1 and Density2 of the previous literatures

Support	Catalyst	Density1 ($atom/nm^2$) [a]	Density2 ($atom/nm^2$) [b]	Density2/ Density1	Reference
Metal oxides	Pt_1/CeO_2	0.99	1.00	1.0	D. Kunwar et al., ACS Catal. 2019, 9, 3978–3990
	Pt_1/MnO_2	0.34	0.38	1.1	L. Kuai et al., Chem. Mater. 2018, 30, 5534–5538
	Pt_1/Fe_2O_3	1.20	1.00	0.8	S. Duan et al., Nanotechnology, 2018, 29, 204002
	Pt_1/FeO_x	0.09	0.07	0.8	B. Qiao et al., Nat. Chem. 2011, 3, 634–641
Carbon	Pt_1/N-C	0.086	2.033	23.6	Y. Zhu et al., ACS Catal. 2018, 8, 10004–10011
	Pt_1/N-C	0.064	0.281	4.4	Z. Chen, et al., Adv. Funct. Mater. 2017, 27, 1605785
	Pt_1/N-C	0.011	0.143	13.0	J. Liu et al., Nat. Commun. 2017, 8, 15938
	Pt_1/N-C	0.008	0.319	39.9	R. Kamai et al., Angew. Chem. Int. Ed. 2016, 55, 13184–13188
	Pt_1/N-C	0.022	0.175	8.0	Our work

[a] Density1 = (Metal loading × N_A)/(Molar mass × BET surface area). N_A is Avogadro's constant. [b] Density2 = (the number of bright dots) / (AC HAADF-STEM image area).

Comment 2: *When performing catalysis, especially when the nanoparticles are more active, the authors must present characterization of the catalyst after reaction, to demonstrate that the structure is maintained.*

Response 2: We thank the reviewer for the suggestion. As shown in Fig. R3, no Pt nanoparticles or nanoclusters were found in TEM and STEM images, and the corresponding AC HAADF-STEM images revealed that the Pt species maintained the atomically dispersed states after the hydrogenation reactions. And we have added the discussion in the revised manuscript (Supplementary Fig. 39).

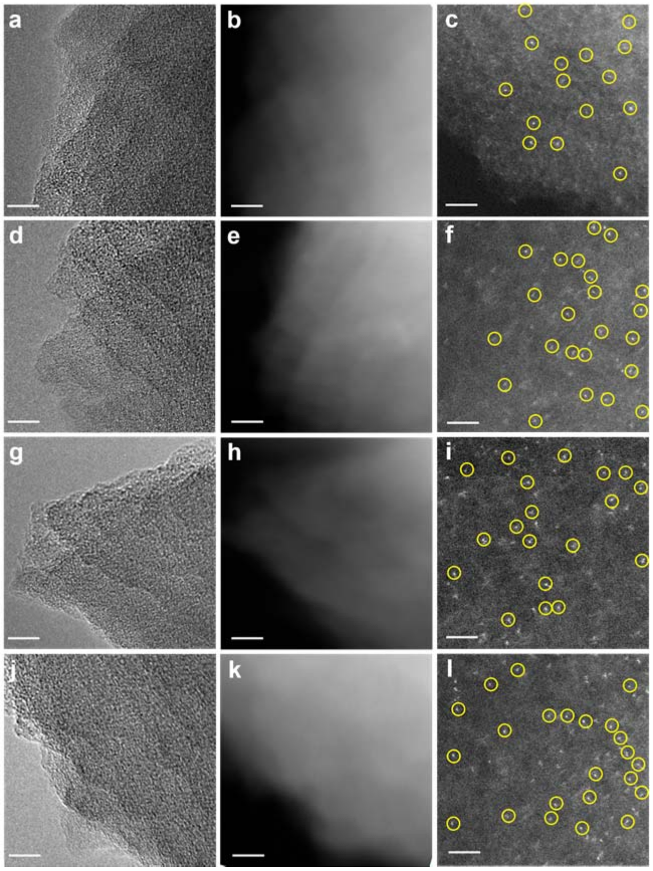

Fig. R3 TEM, STEM, and AC HAADF-STEM images of Pt_1/N-C after 5 catalytic runs of hydrogenation of **a–c** 1-nitro-4-ethynylbenzene, **d–f** 1-ethynyl-4-vinylbenzene, **g–i** 1-ethynyl-4-(phenylethynyl)benzene, and **j–l** 1-(dec-1-yn-1-yl)-3-ethynylbenzene, respectively. Scale bar, 10 nm for TEM/STEM images, 2 nm for AC HAADF-STEM images.

Comment 3: *During the selective hydrogenation, it appears that the single atom catalyst is much less active, but highly selective. It is expected that selectivity for such reactions is*

influenced by conversion, since at higher conversion the molecule has a chance to get hydrogenated at multiple sites. The authors need to present results for selectivity as a function of conversion. It is not enough to state that conversion was less than 20% since that does not tell us the conversion for each sample.

<u>Response 3:</u> Following the suggestion of the reviewer, we did the selective hydrogenation reaction of several substrates on Pt_1/N-C of longer reaction time (Fig. R4). When the conversions increased to ~100%, the selectivities to the target products still maintained >96%, which clearly indicated that Pt_1/N-C was highly selective in these reactions. And we have added the results in the revised manuscript (Supplementary Fig. 36).

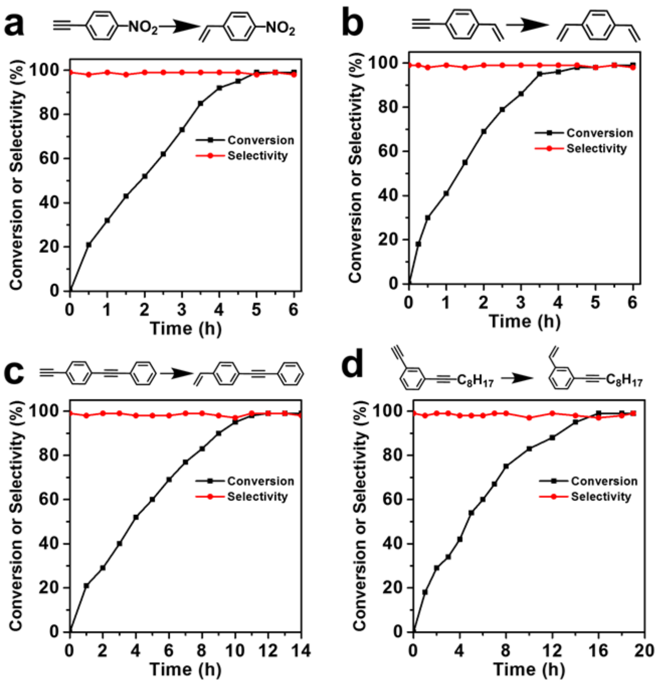

Fig. R4 Selective hydrogenation of **a** 1-nitro-4-ethynylbenzene, **b** 1-ethynyl-4-vinylbenzene, **c** 1-ethynyl-4-(phenylethynyl)benzene, and **d** 1-(dec-1-yn-1-yl)-3-ethynylbenzene on Pt_1/N-C. Reaction condition: substrate (0.5 mmol), Pt_1/N-C (Pt:substrate = 1:1200, mol:mol), methanol (2.0 mL), H_2 (1.0 MPa). Reaction temperature: **a** and **b** 50 °C; **c** and **d** 80 °C.

Reviewer #2:

Comment 1: *In addition to STEM images, the authors need to provide EXAFS data for all the SACs reported in this work. Since the central claim of this work is the synthesis of a library of SACs. The experimental evidence has to be convincing.*

Response 1: We thank the reviewer for the suggestion. Although with very tight beamtime schedule, we still tried out best to measure SACs as many as we can. As shown in Fig. R5, the EXAFs profiles of 17 SACs covering noble metals (Ru, Rh, Pd, Ir, and Pt), transition metals (V, Cr, Mn, Fe, Co, Ni, Cu, Zr, Mo, and W), and main group metals (Ga and Sn) were provided. Gratifyingly all of them revealed the absence of the metal-metal bond, which clearly demonstrated the atomically dispersed nature of the metal species, in good agreement with the results of AC HAADF-STEM. It is very difficult to get the EXAFS results for the other 7 samples (Ti, Ag, Cd, In, Er, Au, and Bi) mainly because the metal loadings were too low to obtain the high quality EXAFS results. And we have added the discussion in the revised manuscript (Supplementary Fig.29).

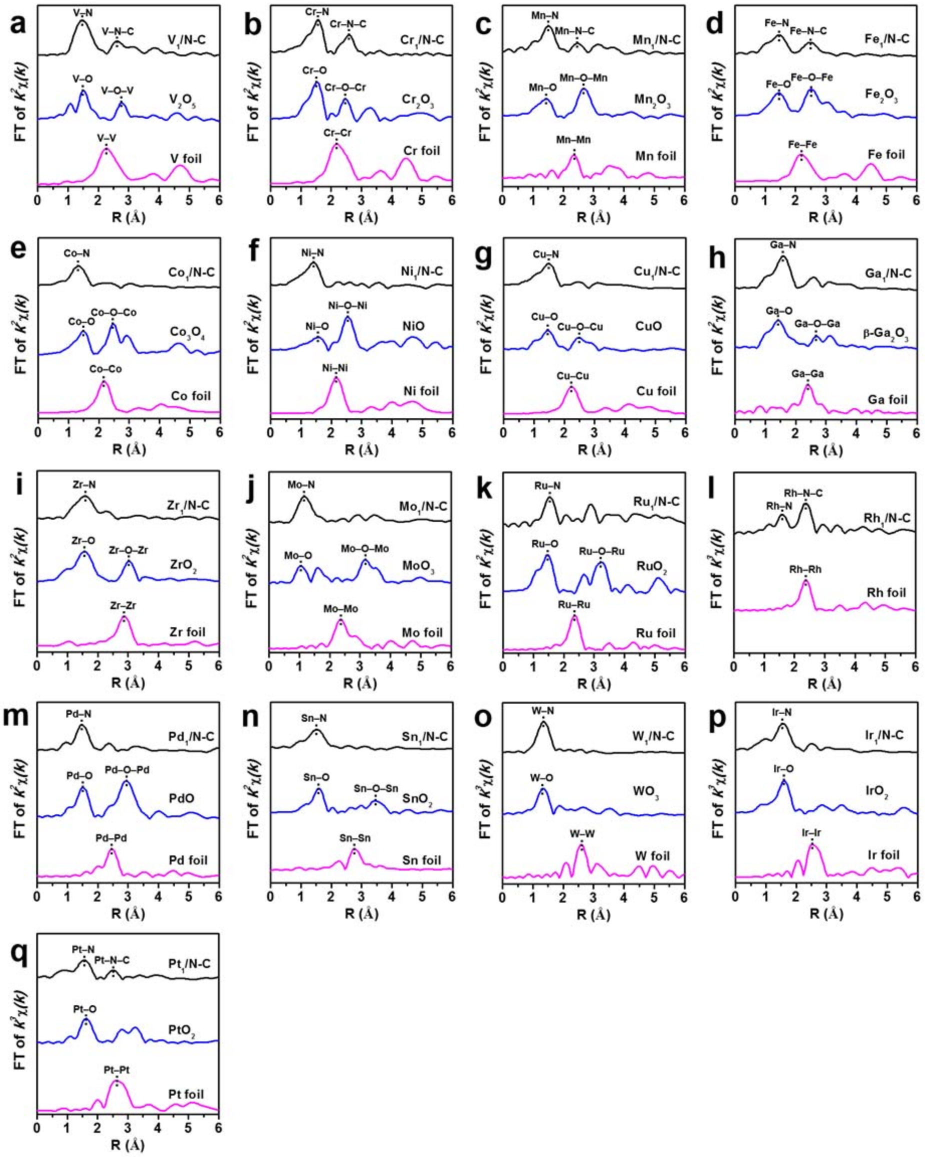

Fig. R5 EXAFS data for M_1/N-C, corresponding metal oxides and metal foils (M= V, Cr, Mn, Fe, Co, Ni, Cu, Ga, Zr, Mo, Ru, Rh, Pd, Sn, W, Ir, and Pt, respectively).

Comment 2: *The authors have to comment the atomic structures of all the SACs reported in this work. Do all the SACs have the same atomic structure? The authors need to provide evidence.*

Response 2: We thank the reviewer's comment. We fitted the EXAFs profiles of 17 SACs. As shown in Fig. R6 and Table R3. The coordination numbers of the metal centers with surrounding N atoms extracted from the EXAFs result was about 4 (Table R3; M = Cr, Mn, Fe, Co, Ni, Cu, Ga, Zr, Rh, Pd, Sn, W, Ir, and Pt), indicating that most of the SACs possessed a MN_4-like structure inherited from the precursors of metalloporphyrins (X. Fang et al., Adv.

Mater. 2018, 30, 1705112; Y. Han et al., J. Am. Chem. Soc. 2017, 139, 17269–17272). However, due to the extremely low metal loadings, the signal-to-noise ratios of some SACs (V, Mo and Ru) were not satisfactory, leading to significant ghost-peaks or peak shifting in R space spectrum so that fitting results became unreliable, making it difficult to determine their atomic structures only based on the EXAFS results. However, combining EXAFS and AC HAADF-STEM results together, it is easy to conclude the atomically dispersed structure of all the samples in the manuscript. We have made this clear in the revised manuscript.

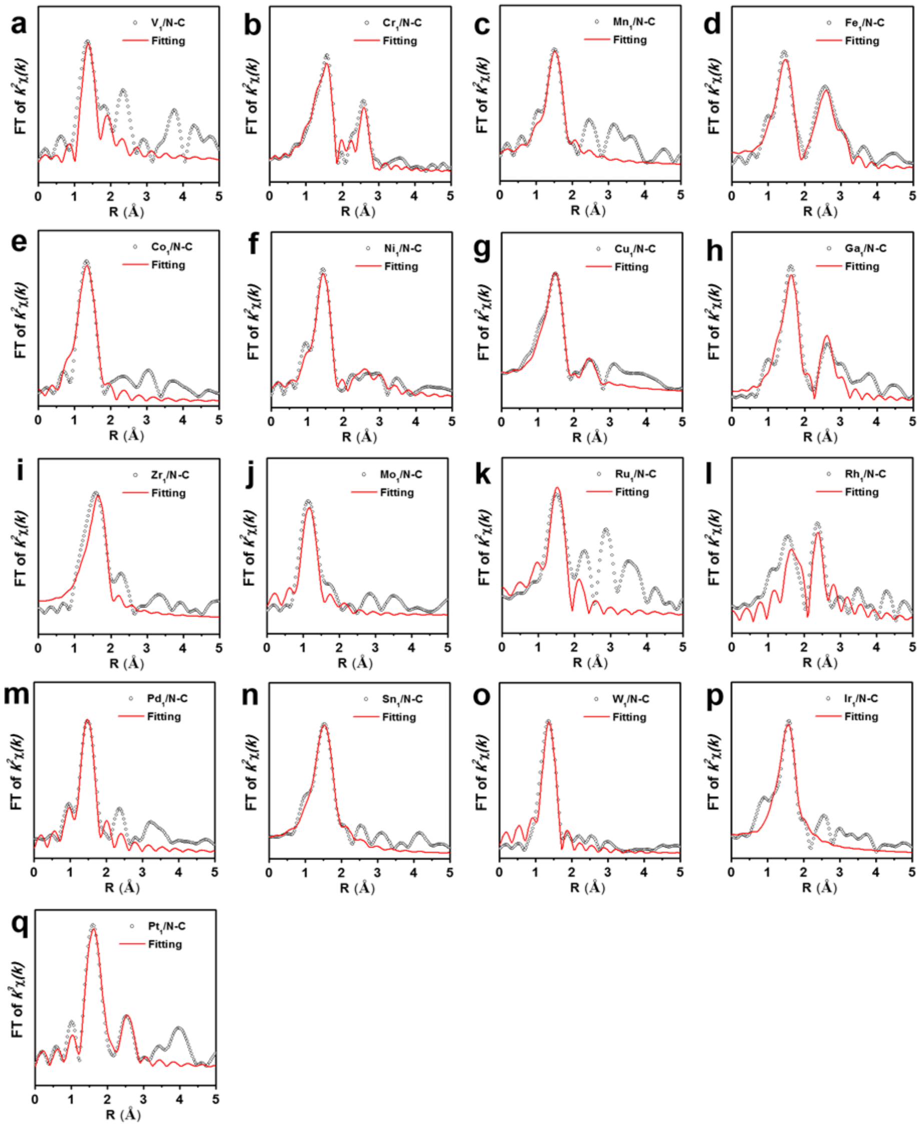

Fig. R6 EXAFS fitting for M_1/N-C (M= V, Cr, Mn, Fe, Co, Ni, Cu, Ga, Zr, Mo, Ru, Rh, Pd, Sn, W, Ir, and Pt, respectively).

Table R3. Structural parameters of EXAFS fitting for the M_1/N-C

Sample	Shell	C.N. [a]	σ^2 ($\times 10^{-3}$ Å^2) [b]	E_0 (eV) [c]	R (Å) [d]
V_1/N-C	V–N	1.8	6.1	30.9	1.63±0.06
Cr_1/N-C	Cr–N	2.7	3.0	-6.2	1.95±0.04
	Cr–N–C	4.1	0.3	-6.2	3.42±0.07
Mn_1/N-C	Mn–N	3.7	3.5S	1.3	2.03±0.08
Fe_1/N-C	Fe–N/O	6.6	13.0	-0.5	2.02±0.13
	Fe–N–C	4.3	6.8	-0.5	2.90±0.15
Co_1/N-C	Co–N/O	5.0	8.6	-17.5	1.89±0.09
Ni_1/N-C	Ni–N	3.0	5.5	-2.0	1.85±0.05
	Ni–N–C	1.0	1.8	-2.0	2.72±0.10
Cu_1/N-C	Cu–N	3.0	5.5	0.4	1.92±0.01
	Cu–N–C	4.0	7.3	6.7	2.90±0.05
Ga_1/N-C	Ga–N	3.3	5.4	4.1	2.00±0.12
	Ga–N–C	2.4	3.4	4.1	3.07±0.14
Zr_1/N-C	Zr–N	3.9	6.5	-1.0	2.18±0.01
Mo_1/N-C	Mo–N	1.4	1.0	1.6	1.82±0.43
Ru_1/N-C	Ru–N	1.1	5.1	6.5	2.10±0.06
Rh_1/N-C	Rh–N	2.4	0.1	12.0	2.16±0.43
	Rh–N–C	5.8	0.1	12.0	3.16±0.35
Pd_1/N-C	Pd–N	2.3	2.8	-1.2	1.96±0.02
Sn_1/N-C	Sn–N	3.8	7.5	8.8	2.03±0.01
W_1/N-C	W–N	3.5	1.2	9.7	1.73±0.03
Ir_1/N-C	Ir–N	3.5	6.9	4.9	1.98±0.02
Pt_1/N-C	Pt–N	3.4	3.0	0.7	2.03±0.02
	Pt–N–C	2.2	2.5	5.4	2.98±0.04

[a] C.N.: coordination number; [b] σ^2: Debye-waller factors; [c] E_0: the inner potential correction. [d] R: bond distance;

Comment 3:*The authors have shown a successful synthesis of bimetallic Pt and Sn SACs site. Does it apply for other metal species?*

Response 3: We thank the reviewer for the suggestion. Our strategy is of great flexibility and here we give another example, the successful synthesis of trimetallic SACs (Pt_1-Sn_1-Cu_1/N-C, Fig. R7). With the molar ratio of PtTPP:SnTPP:CuTPP:TPP (1:1:1:40) and the same synthesis procedure as previously described, the nitrogen-doped porous carbon based materials with 0.45 wt% Pt loading, 0.28 wt% Sn loading and 0.18 wt% Cu loading were obtained. This ratio of the metal loading for Pt, Sn and Cu (2.5:1.6:1) was in good agreement with the nominal ratio (3.0:1.8:1), based on the molar ratio of PtTPP:SnTPP:CuTPP (1:1:1) and atomic weight ratio of Pt:Sn:Cu (195.1:118.7:63.5). No nanoparticles were observed in TEM/STEM images and no corresponding XRD peaks emerged. Additionally, in the AC

HAADF-STEM image, the homogeneously distributed bright dots tagged by yellow circles can be attributed to Pt, or Sn or Cu atoms. Corresponding element mapping analysis of Pt_1-Sn_1-Cu_1/N-C revealed that Pt, Sn and Cu species were homogeneously distributed. Combined all the results above, it can be concluded that all three metal species were atomically dispersed on the porous carbon matrix, i.e. the synthesis of Pt_1-Sn_1-Cu_1/N-C was achieved. Surely, we will report the strategy for multi-metal SACs construction in our future publication.

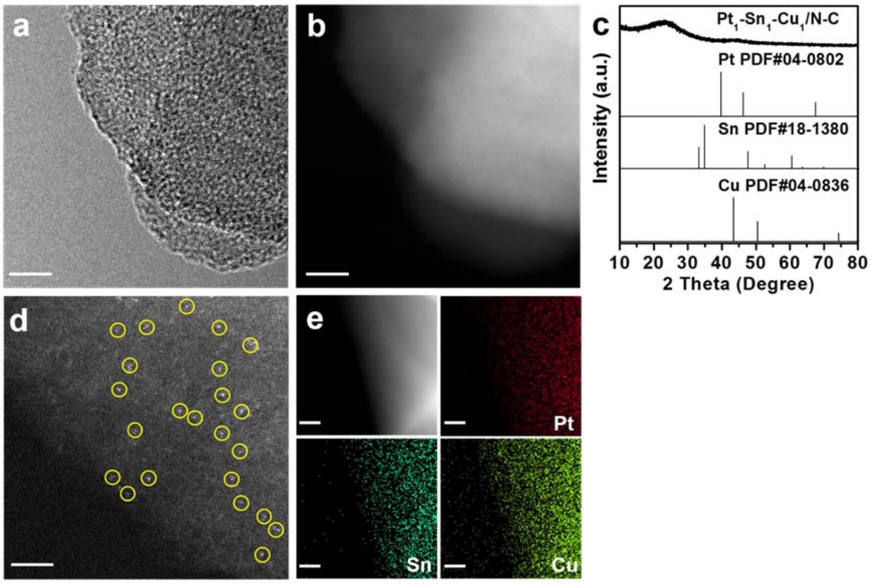

Fig. R7 **a** TEM and **b** STEM of Pt_1-Sn_1-Cu_1/N-C. Scale bar, 10 nm. **c** XRD pattern of Pt_1-Sn_1-Cu_1/N-C. **d** AC HAADF-STEM of Pt_1-Sn_1-Cu_1/N-C. Scale bar, 2 nm. **e** element mapping of Pt_1-Sn_1-Cu_1/N-C. Scale bar, 100 nm.

<u>Comment 4:</u> *The stability of Pt_1/N-C SAC for the reaction shall be evaluated.*

<u>Response 4:</u> We thank the reviewer for the nice suggestion. The recycling experiments of Pt_1/N-C catalysts in four representative hydrogenation reactions were conducted for five runs (Fig. R8). Clearly, no obvious decline in catalytic activity and selectivity were observed. Combined with the results that the structure of the catalysts were well maintained after reactions (see Response 2 for Reviewer 1), we concluded that Pt_1/N-C catalyst is stable under our reaction conditions. We have added the result in the revised manuscript (Supplementary Fig. 38).

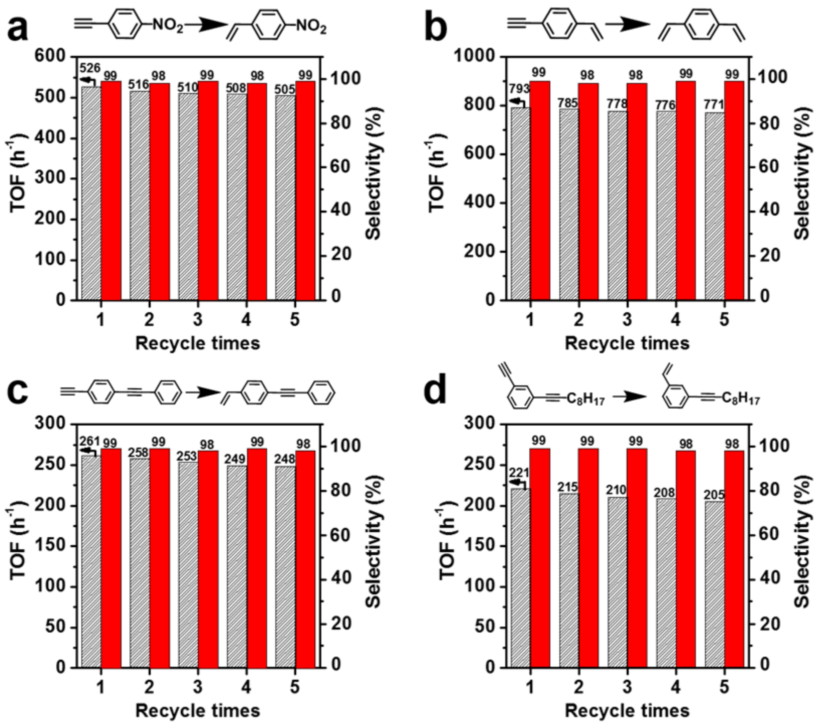

Fig. R8 Catalytic performance of Pt_1/N-C in hydrogenation of **a** 1-nitro-4-ethynylbenzene, **b** 1-ethynyl-4-vinylbenzene, **c** 1-ethynyl-4-(phenylethynyl)benzene, and **d** 1-(dec-1-yn-1-yl)-3-ethynylbenzene for 5 catalytic runs. Reaction condition: substrate (0.5 mmol), catalyst (Pt:substrate = 1:1200, mol:mol), methanol (2.0 mL), H_2 (1.0 MPa). Reaction temperature: **a** and **b** 50 °C; **c** and **d** 80 °C. All the conversions were maintained at ~20%.

Response to Referees' Comments

We thank the referees for their valuable comments and positive endorsement to our manuscript. We have carefully considered the referees' comments and revised the manuscript. Our responses and corresponding revisions are as follows:

Response to the Reviewer #1 Comments:

Reviewer #1:

The authors have addressed all the concerns expressed by the reviewers. The manuscript makes an important contribution and is suitable for publication, but the authors do need to address my comments # 1–3 which I will discuss below:

Response: We appreciate the reviewer's comment. And all the comments and suggestions were considered carefully and incorporated completely in the revision (see below for a point-to-point response).

Comment 1: *The authors question my assertion that the limited depth of focus makes it possible to consider each image as representing a plane, allowing counting of the number of atoms per unit area. They state that the depth of focus is ~10 nm, which they suggest allows use of this method for oxide samples but not for carbon samples, since the interplanar distance is 0.34 nm. However, this argument is not valid since their computation of density1 considers the number of atoms per unit area based on BET surface area and total metal loading. Therefore, the schematic they have drawn in Figure R2 is not valid since the layers of graphite shown in this diagram would not be accessible to* N_2 *for BET surface area measurement. This schematic would be reasonable if this was an intercalated structure which it is not.*

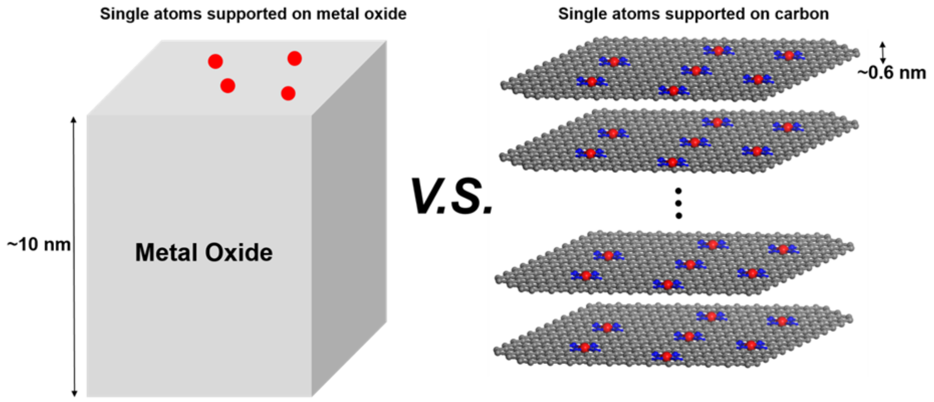

Fig. R9 Illustration of single atoms catalyst supported on metal oxides or carbon.

AC‐STEM imaging in the HAADF imaging relies on the fact that only a thin region of sample is being imaged due to the limited depth of focus. Typically the images of single atoms are very sensitive to defocus. Small changes in focus influence the contrast of single atoms, which is why I stated that the depth of focus is very small. Otherwise the contrast from a single atom of Pt would not be brighter than the contrast from 10 nm of CeO_2 as seen in the schematic above.

While it is true that this imaging is localized and may not fully represent the average, but if this is the case, more representative images should be presented. The images are important for the study of single atom catalysts.

Response 1: We thank the reviewer's comment. First, we think our samples were mainly composed of the loosely packing N-doped carbon, so even at the margin of the sample, there were possibly more than one layer (see Fig. R10). Also, we agree with the reviewer that the images of single atoms are very sensitive to defocus. However, according to the reference (P. D. Nellist et al., Microsc. Microanal. 2008, 14, 82–88), the depth of focus of the ARM 200F is about 10 nm. Up to now, we do not understand what the reason is, and we withdraw the schematic of the imaging of single atoms supported on oxide supports. It is not the key point in this work.

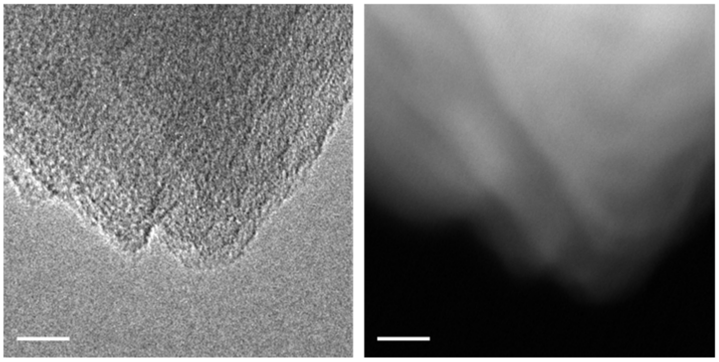

Fig. R10 TEM and STEM images of Pt_1/N-C. Scale bar, 10 nm.

In addition, as we previously claimed, the TEM imaging was the localized observation method and the images selected cannot be regarded as statistic of the overall situation. So we took more than one images for every sample, and here we showed other two images of the catalyst of Pt_1/N-C (Fig. R11). Furthermore, EXAFS measurements were also performed to reveal the dispersion state of the metal species, and all of them indicated the dominant

presence of the isolated atoms (see Supplementary Fig. 29 in the revised manuscript), in agreement with the AC HAAFD-STM observation.

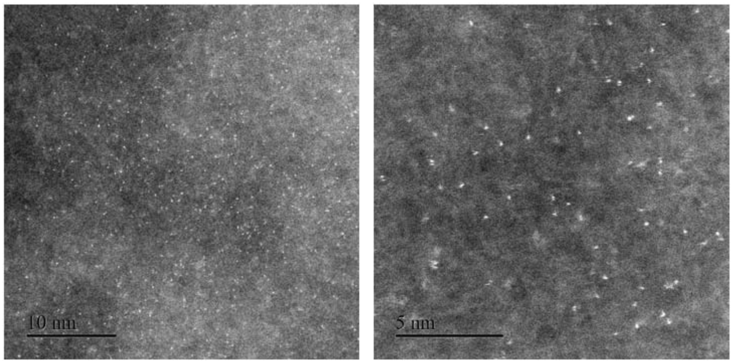

Fig. R11 AC HAADF-STEM images of Pt_1/N-C.

<u>Comment 2:</u> *The authors present Figure R1 to show that the blank carbon does not show any bright single atom contrast. This is because of the size of the image and the contrast the authors used. I magnified this image and reproduced it below. You can see how very similar contrast can be seen, but this is speckle caused by the carbon atoms. This is the reason why I questioned the way the authors circled some bright dots and not others.*

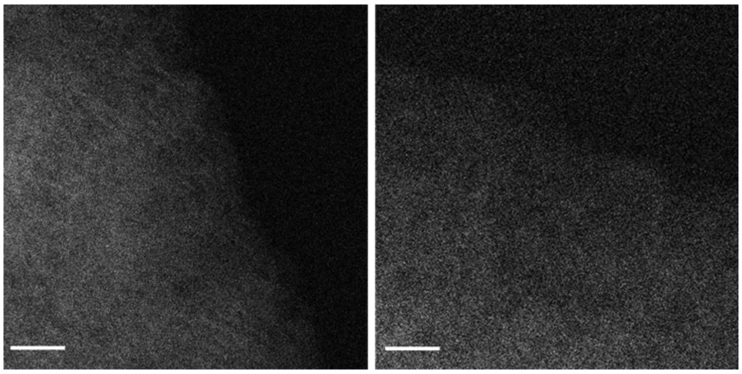

Fig. R12 AC HAADF-STEM of N-C. Scale bar, 2 nm.

<u>Response 2:</u> We thank the reviewer's comment. First, in order to evaluate the brightness of the circled dots, the Intensity-Distance tool of the Digitalmicrograph software was used and the results were shown below (Fig. R13). For the image of N-C, there was only chaos. For the images of Ti_1/N-C and Mo_1/N-C, there were obvious corresponding peaks, respectively, which indicated the isolated metal atoms. These results above clearly confirmed the rationality of our method. Additionally, following the reviewer's suggestion, we checked the circled bright dots in our images, and then improved the results (e.g. added some circled

bright dots in the images, such as Ir_1/N-C, Au_1/N-C).

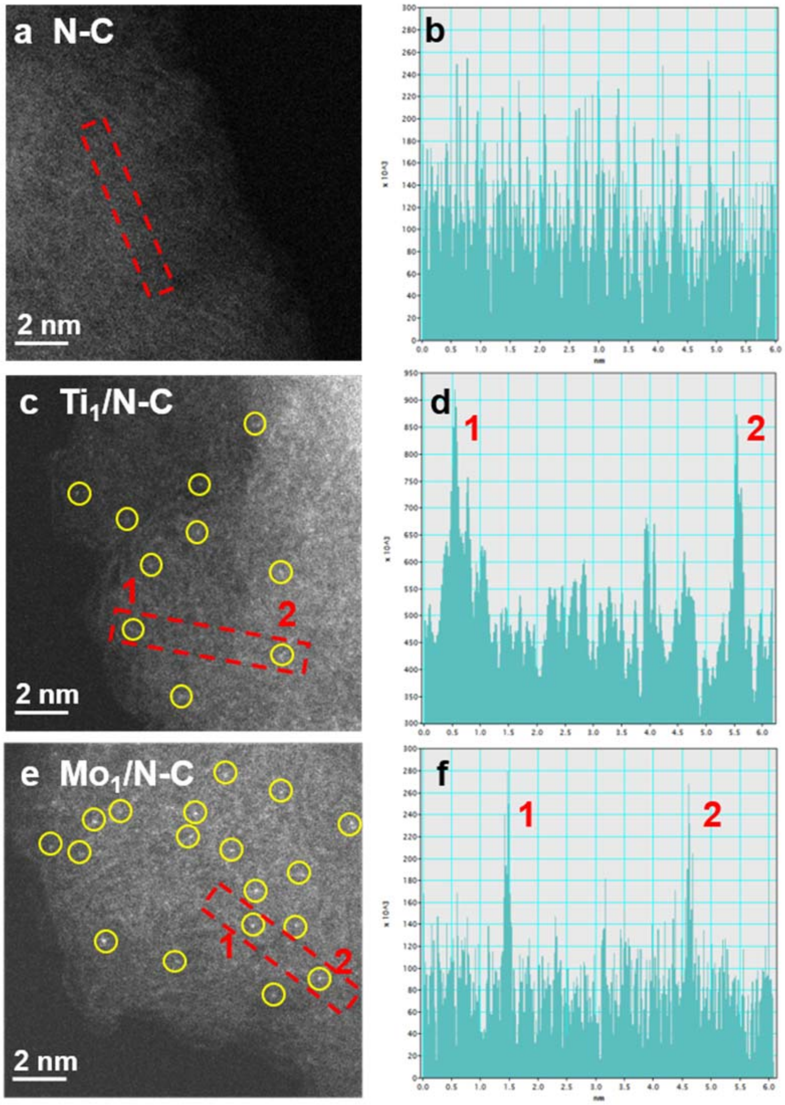

Fig. R13 AC HAADF-STEM (left) and intensity profile (right) along the X-Y in the HAADF-STEM images. a–b for N-C, c–d for Ti_1/N-C, and e–f for Mo_1/N-C.

Comment 3: *I am not sure how the authors are counting atoms. If I examine Figure 1d below, I count 38 atoms of Pt. Assuming the scale bar is 10 nm, the field of view is 65 nm across. So, I estimate the density2 to be 38/(65)^2 = 0.00899 atoms/nm^2. I am not sure how the authors are deriving the numbers in their table R1.*

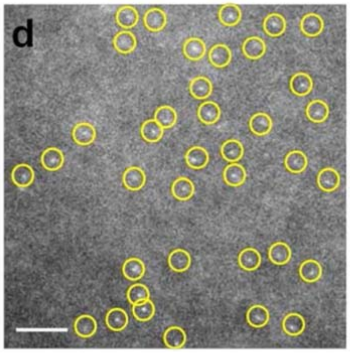

Fig. R14 AC HAADF-STEM image of Pt_1/N-C. Scale bar, 2 nm.

Likewise, I come up with very different numbers for their other atom densities. I selected one of the images from Figure 2. First, the expanded view of this images shows the difficulty I have when the authors circle a few of the bright dots when there are so many others of similar brightness. And then, their counting does not match what I am coming up with. For this image, I see 17 circled atoms and the area covered is ~ 13.5 nm high and 9 nm across, so the area is 121 nm^2. Hence density2 should be 17/121 = 0.14 atoms/nm^2.

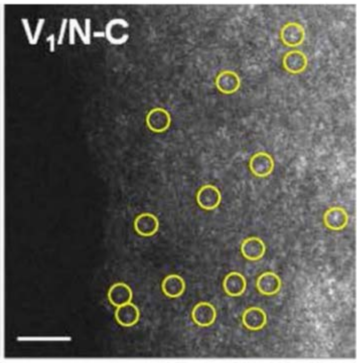

Fig. R15 AC HAADF-STEM images of V_1/N-C. Scale bar, 2 nm.

I would encourage the authors to be careful in circling these atoms and then stating whether or not the surface concentration is consistent, ie is density1 close to density 2?

Response 3: We thank the reviewer's comment. First, the scale bar of Figure 1d in manuscriptwas 2 nm (given in the figure legend), not 10 nm. So the field of view is about 14 nm across (14*14 = 196 nm^2). Therefore, Density2 of Figure 1d in manuscript was 38/196 = 0.19 atoms/nm^2. Second, thank the reviewer for pointing out our mistake that we did not consider some samples did not occupy the whole image, and we calculated Density2 based on the areas of 196 nm^2. Therefore, we re-calculated Density2 based on the actual areas, and the results were shown in Table R4. Seen from Table R1, Density2 was still several times Density1, and the further investigation will be performed to figure out the inherent reason.

Table R4. Metal contents, BET surface areas, Density1, Density2, and their ratios for M_1/N-C catalysts

Sample	M (wt%)	BET (m^2/g)	Density1 (atom/nm^2) [c]	The number of bright dots [a]	Actual area(nm^2)[b]	Density2 (atom/nm^2) [d]	Density2/ Density1
Pt_1/N-C(1:20)	0.73	656	0.034	68	196	0.347	10.2
Pt_1/N-C	0.43	595	0.022	38	196	0.194	8.8
Pt_1/N-C(1:80)	0.21	632	0.010	17	196	0.087	8.7
Pt_1/N-C(1:320)	0.06	644	0.003	5	160	0.031	10.3
Ti_1/N-C	0.12	587	0.026	10	170	0.059	2.3
V_1/N-C	0.19	656	0.034	32	160	0.200	5.9
Cr_1/N-C	0.10	792	0.015	18	145	0.124	8.3
Mn_1/N-C	0.07	790	0.010	12	145	0.083	8.3
Fe_1/N-C	0.18	784	0.025	12	120	0.100	4.0
Co_1/N-C	0.20	834	0.024	9	180	0.050	2.1
Ni_1/N-C	0.22	677	0.033	17	180	0.094	2.8
Cu_1/N-C	0.21	746	0.027	9	170	0.053	2.0
Ga_1/N-C	0.06	841	0.006	8	170	0.047	7.8
Zr_1/N-C	0.05	524	0.006	10	160	0.063	10.5
Mo_1/N-C	0.15	567	0.017	18	150	0.120	7.1
Ru_1/N-C	0.10	877	0.007	12	190	0.063	9.0
Rh_1/N-C	0.08	869	0.005	18	160	0.113	22.6
Pd_1/N-C	0.30	682	0.025	20	150	0.133	5.3
Ag_1/N-C	0.06	799	0.004	11	150	0.073	18.3
Cd_1/N-C	0.32	629	0.027	32	160	0.200	7.4
In_1/N-C	0.09	683	0.007	9	150	0.060	8.6
Sn_1/N-C	0.43	426	0.051	8	160	0.050	1.0
Er_1/N-C	0.06	793	0.003	16	160	0.100	33.3
W_1/N-C	0.27	443	0.020	22	196	0.112	5.6
Ir_1/N-C	0.38	837	0.014	42	180	0.233	16.6
Au_1/N-C	0.19	783	0.007	52	180	0.289	41.3
Bi_1/N-C	0.08	743	0.003	10	180	0.056	18.7
Pt_1-Sn_1/N-C	0.48-0.35	724	0.045	28	150	0.187	4.2

[a] Bright dots is based on the Fig. 2–4 in manuscript. [b] HAADF-STEM image area, the total area minus the blank area (approximate). [c] Density1 = (Metal loading × N_A)/(Molar mass × BET surface area). N_A is Avogadro's constant (6.02×10^{23}). [d] Density2 = (the number of bright dots) / (AC HAADF-STEM image area).

Response to the Reviewer #2 Comments:

Reviewer #2:

The authors have addressed my comments and made the revisions accordingly. Therefore, I recommend the publication of this paper.

Response: We appreciate the reviewer's recommendation.
